# FMNL2 suppresses cell migration and invasion of breast cancer: a reduction of cytoplasmic p27 via RhoA/LIMK/Cofilin pathway

**DOI:** 10.1038/s41420-022-00964-z

**Published:** 2022-04-04

**Authors:** Xinyan Jiao, Bo Wang, Lu Yang, Qingbin Zhao, Miao Zhang, Xiaoxu Liu, Can Zhou, Ruiqi Wang, He Chen, Jichang Wang, Yu Ren, Peijun Liu

**Affiliations:** 1 Center for Translational Medicine, the First Affiliated Hospital of Xi’an Jiaotong University, Xi’an, 710061 Shaanxi P.R. China; 2 Key Laboratory for Tumor Precision Medicine of Shaanxi Province, the First Affiliated Hospital of Xi’an Jiaotong University, Xi’an, 710061 Shaanxi P.R. China; 3 Department of Geratology, the First Affiliated Hospital of Xi’an Jiaotong University, Xi’an, 710061 Shaanxi P.R. China; 4 Department of Breast Surgery, the First Affiliated Hospital of Xi’an Jiaotong University, Xi’an, 710061 Shaanxi P.R. China; 5 Department of Vascular Surgery, the First Affiliated Hospital of Xi’an Jiaotong University, Xi’an, 710061 Shaanxi P.R. China

**Keywords:** Breast cancer, Cell invasion

## Abstract

Formin-like protein 2 (FMNL2) belongs to a highly conserved family of cytoskeletal remodeling proteins that have been reported to be implicated in various actin-dependent physiological and cancer-associated processes. In this study, we mainly investigated the effects of FMNL2 on breast cancer cell migration and invasion, and the underlying mechanisms involved. We found that FMNL2 reduced cell migration and invasion of breast cancer in vitro and in vivo. Further, FMNL2 disrupted actin cytoskeleton rearrangement and hampered the RhoA/LIMK/Cofilin pathway in breast cancer cells. Critically, both Rho inhibitor ZOL and LIMK inhibitor BMS3 significantly abrogated these migration-promoting effects in FMNL2-silencing MDA-MB-231 and BT549 cells. RhoA/LIMK/Cofilin pathway was involved in FMNL2 silencing-induced actin cytoskeleton rearrangement in MDA-MB-231 and BT549 cells. More importantly, cytoplasmic p27 promoted FMNL2-mediated cell migration and invasion through RhoA/LIMK/Cofilin pathway in MCF7 and MDA-MB-231 cells. In addition, the expression and prognosis of FMNL2 were associated with ER in breast cancer. Furthermore, ERα overexpression reduced the protein levels of FMNL2 in breast cancer cells, which were reversed by MG132. In conclusion, FMNL2 suppressed cell migration and invasion of breast cancer by inhibiting RhoA/LIMK**/**Cofilin pathway through a reduction of cytoplasmic p27. This finding implies that the interference of FMNL2-mediated RhoA/LIMK/Cofilin pathway involving the cytoplasmic p27 may be a promising strategy for ameliorating breast cancer metastasis and prognosis.

## Introduction

Currently, breast cancer is unquestionably the leading cause of cancer-related deaths in women aged under 45 years [1, 2]. Accumulating evidence indicates that tumor aggressiveness and metastatic feature are the key determinants of breast cancer outcomes. Importantly, distant site metastasis has been the main cause of mortality in breast cancer patients [3]. Hence, there is a pressing need for searching new therapeutic targets and getting a better understanding of cancer metastasis underlying tumor progression to alleviate breast cancer outcomes.

Formins belong to a highly conserved family of cytoskeletal remodeling proteins that have been reported to be implicated in various actin-dependent physiological and cancer-associated cellular processes [4]. Formins mainly control cell shape, mobility, adhesion, morphogenesis, and cytokinesis by actin cytoskeleton reorganization [5]. Current evidence further indicates a crucial role for formins in cancer progression [6]. In mammals, there are 15 formins identified and grouped into 8 different sub-families based on their sequences and domain architectures. FMNL subfamily has been profoundly involved in specific tumor progression and metastasis [7–9]. However, to our knowledge, the role of formin-like protein 2 (FMNL2) in the progression of various types of cancers was still under debate.

A growing body of evidence suggests that there is a discrepancy in FMNL2 expression in multiple cancers, for instance, upregulated in colorectal cancer [10], melanoma [7], and gastric cancer [11], but downregulated in hepatocellular carcinoma [12]. Studies have shown that FMNL2 is associated with cellular polarity, and loss of cell polarity leads to tumorigenesis and metastasis. Especially, aberrant expression and localization of polarity proteins are closely related with epithelial tumors such as breast cancer. Moreover, FMNL2 has been reported to be required in breast epithelialization [13]. In our previous study, FMNL2 is inconsistently expressed in the cytoplasm and nucleus of non-cancerous breast epithelial MCF10A cells and breast cancer cells, and further, nuclear p27 levels play a crucial role in FMNL2-mediated breast cancer cell proliferation [14]. Considering cytoplasmic 27 has been reported to be implicated in invasion and metastasis of cancer progression [15], we speculated whether cytoplasmic 27 was involved in FMNL2-mediated cell migration and invasion of breast cancer. Thus, in this study we mainly explored the role of FMNL2 in cell migration and invasion of breast cancer, and the possible underlying mechanisms involved.

## Results

### FMNL2 reduced cell migration and invasion of breast cancer in vitro and in vivo

**Fig. 1:**
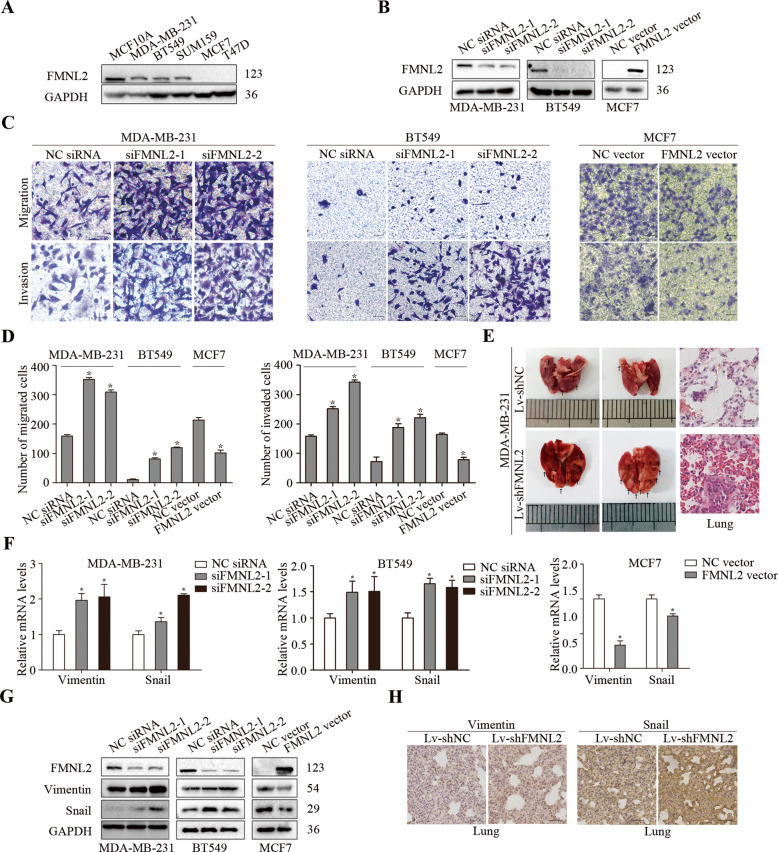
Effects of FMNL2 on cell migration and invasion of breast cancer in vitro and in vivo. After transfection for 48 h, cultured cells were processed for indicated assays. **A** The protein levels of FMNL2 were measured by western blotting in human breast cancer cells. **B** The silencing efficiency and overexpression of FMNL2 were measured by western blotting in human breast cancer cells. **C**, **D** Representative images and quantified data of transwell assay were shown. Scale bar, 50 μm. **E** Representative lung metastasis in mice and HE staining of lung sections. Scale bar, 35 μm. **F**, **G** The levels of Vimentin and Snail were examined using qRT-PCR and western blotting. **H** The IHC staining for Vimentin and Snail in lung tissues was shown. Scale bar, 50 μm. *P < 0.05 *versus* corresponding control group.

Firstly, we found that FMNL2 protein was highly expressed in non-cancerous MCF10A cells when compared to breast cancer cells. Intriguingly, FMNL2 was weakly expressed in MCF7 and T47D cells, but positively expressed in MDA-MB-231, BT549, and SUM159 cells (Fig. 1A). Thus, FMNL2 was silenced in MDA-MB-231 and BT549 cells, and overexpressed in MCF7 cells for functional experiments (Fig. 1B and S1A). Live cell imaging and wound-healing analysis indicated that FMNL2 silencing apparently accelerated cell motility in MDA-MB-231 cells (Fig. S1B–E). Transwell assay further showed that the migratory and invasive abilities were strongly enhanced by FMNL2 silencing in MDA-MB-231 and BT549 cells, and reduced by FMNL2 overexpression in MCF7 cells (Fig. 1C, D). Furthermore, mice injected with Lv-shFMNL2/MDA-MB-231 cells significantly increased lung metastasis (Fig. 1E). Additionally, the mRNA and protein levels of Vimentin and Snail were distinctly elevated in FMNL2-silencing MDA-MB-231 and BT549 cells, and inhibited in FMNL2-overexpressing MCF7 cells (Fig. 1F, G). FMNL2 knockdown greatly increased Vimentin and Snail levels in lung tissues of mice (Fig. 1H). These results proved that FMNL2 reduced breast cancer cell migration and invasion in vitro and in vivo.

### FMNL2 disrupted the rearrangement of actin cytoskeleton and hampered the RhoA/LIMK/Cofilin pathway in breast cancer cells

**Fig. 2:**
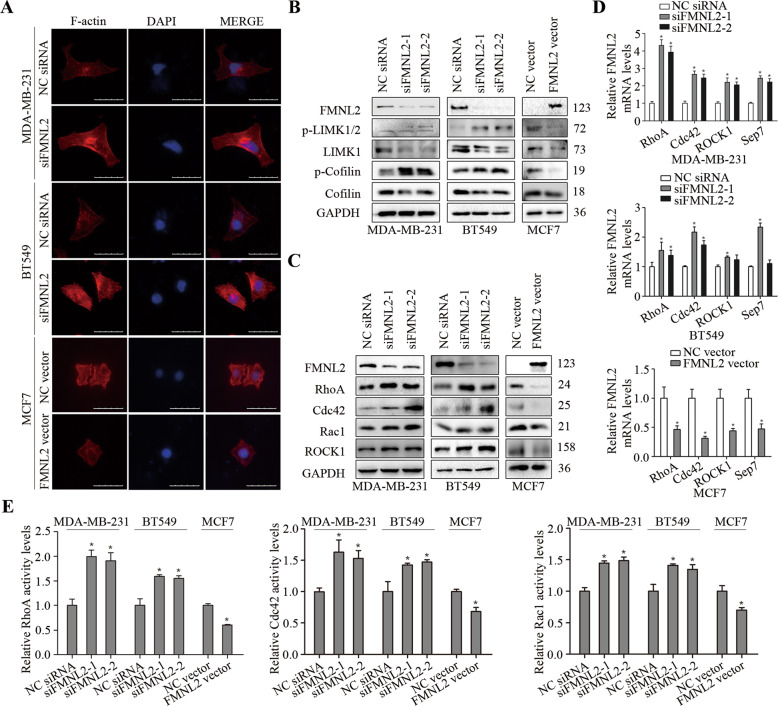
Effects of FMNL2 on cytoskeleton architecture and RhoA/LIMK/Cofilin pathway in breast cancer cells. After transfection for 48 h, cultured cells were processed for indicated assays. **A** Representative fluorescent images of F-actin cytoskeleton were displayed. Scale bar, 50 μm. **B** The levels of LIMK/Cofilin-related molecules were determined by western blotting. **C** The levels of RhoA/ROCK1-related molecules were determined by western blotting. **D** The levels of RhoA, Cdc42, ROCK1 and Sep7 mRNA were measured by qRT-PCR. **E** The activity levels of RhoA, Cdc42 and Rac1 were measured. *P < 0.05 *versus* corresponding control group.

It is generally accepted that the disruption of the actin cytoskeleton inhibits cell migration in cancer progression [16]. Compared with the control, more actin stress fibers were confined to the cell border and became strengthened in FMNL2-silencing MDA-MB-231 and BT549 cells. Meanwhile, the actin fibers at the cell edges seemed less obvious, and F-actin distribution was nearly disappeared in FMNL2-overexpressing MCF7 cells (Fig. 2A). Functionally, F-actin depolymerization and severing factor Cofilin acts as a critical mediator that disassembles actin filaments to control actin cytoskeleton reorganization [17], and LIMK is a downstream effector of Rho/ROCK pathway [18]. Therefore, it was next investigated whether LIMK/Cofilin pathway and Rho-associated factors was involved in FMNL2-mediated breast cancer cell migration and invasion. FMNL2 silencing evidently upregulated the levels of phosphorylated LIMK and Cofilin, and further resulted in an accumulation of RhoA, Cdc42, Rac1, ROCK1, and Sep7 in MDA-MB-231 and BT549 cells, and FMNL2 overexpression resulted in opposite effects in MCF7 cells (Fig. 2B–D). Especially, the activity levels of crucial members of Rho family, including RhoA, Cdc42 and Rac1 were elevated in FMNL2-silencing MDA-MB-231 and BT549 cells, and reduced in FMNL2-overexpressing MCF7 cells (Fig. 2E). In all, these data suggest that the effects of FMNL2 on breast cancer metastasis may be contributed by the regulation of RhoA/LIMK/Cofilin pathway.

### RhoA/LIMK/Cofilin pathway was involved in FMNL2 silencing-induced actin cytoskeleton rearrangement in MDA-MB-231 and BT549 cells

**Fig. 3:**
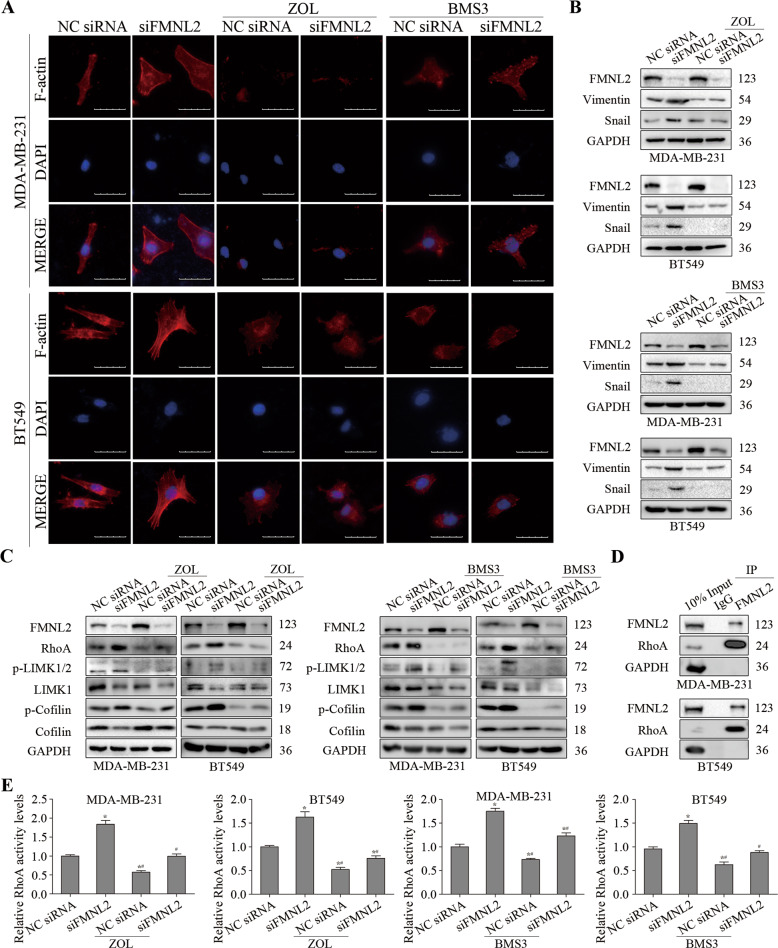
Effects of Rho and LIMK inhibition on cytoskeleton rearrangement and RhoA/LIMK/Cofilin pathway in FMNL2-silencing breast cancer cells. After transfection, MDA-MB-231 and BT549 cells were incubated with ZOL (100 or 70 μM) or BMS3 (20 or 10 μM) for 24 h, respectively, and then performed for indicated assays. **A** Representative fluorescent images of F-actin cytoskeleton were displayed. Scale bar, 50 μm. **B** The levels of FMNL2, Vimentin and Snail were measured by western blotting. **C** The levels of RhoA/LIMK/Cofilin-related molecules were examined by western blotting. **D** Co-IP analysis was used to determine the relationship between FMNL2 and RhoA. **E** The activity levels of RhoA were measured. *P < 0.05 *versus* NC siRNA group, ^#^*P* < 0.05 *versus* siFMNL2 group.

Zoledronic acid (ZOL), a potent aminobisphosphonate targeting the mevalonate pathway, was used to block Rho pathway [19]. BMS3 was a potent LIMK inhibitor for LIMK1 and LIMK2 [20]. Indeed, ZOL and BMS3 treatment obviously induced a spotty and scattered distribution pattern of actin filaments. It is noteworthy that enhancement of the organization of actin fibers into bundles and elevation of Vimentin and Snail was interfered by ZOL and BMS3 treatment in FMNL2-silencing MDA-MB-231 and BT549 cells, indicating that Rho inhibition and LIMK inhibition had the potential to impede cell migration and invasion promoted by FMNL2 silencing in breast cancer (Fig. 3A, B). After incubation with ZOL or BMS3, FMNL2 expression was profoundly improved in MDA-MB-231 and BT549 controls, implying that FMNL2 elevation may be involved in ZOL or BMS3-induced suppressive role in breast cancer cell migration. Moreover, treatment with ZOL and BMS3 blocked the augmented RhoA protein amount and activity levels, as well as the elevated phosphorylation of LIMK and Cofilin in FMNL2-silencing MDA-MB-231 and BT549 cells (Fig. 3C, E). Besides, the interaction between FMNL2 and RhoA was confirmed (Fig. 3D). Overall, our results manifested that RhoA/LIMK/Cofilin pathway was involved in FMNL2 silencing-induced actin cytoskeleton rearrangement in breast cancer cells.

### Cytoplasmic p27 promoted FMNL2-mediated cell migration and invasion through RhoA/LIMK/Cofilin pathway in breast cancer cells

**Fig. 4:**
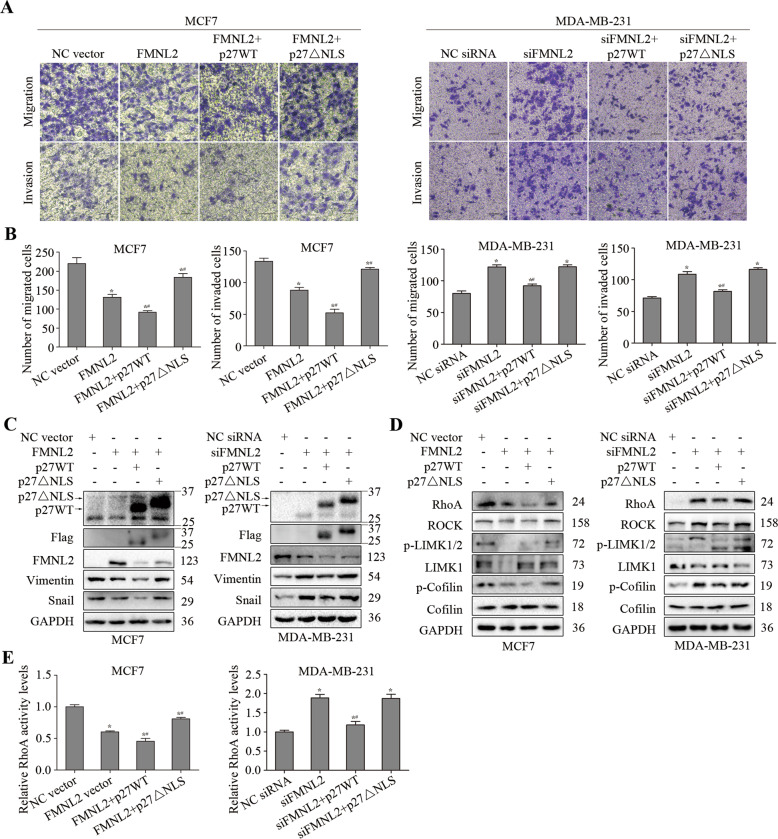
Effects of cytoplasmic p27 on FMNL2-mediated cell migration and invasion in breast cancer cells. After transfection for 48 h, cultured cells were processed for indicated assays. **A, B** Representative images and quantified data of transwell assay were shown. Scale bar, 50 μm. **C** The levels of p27, Flag, FMNL2 and invasion-related molecules were examined by western blotting. **D** The levels of RhoA/LIMK/Cofilin-related molecules were examined by western blotting. **E** The activity levels of RhoA were measured. *P < 0.05 *versus* NC vector or NC siRNA group, ^#^*P* < 0.05 *versus* FMNL2 vector or siFMNL2 group.

It’s well known that p27 is a negative regulator of cell proliferation; whereas p27 can positively control other cellular processes including cell migration and invasion through cytoplasmic localization [21]. Here we clearly observed that p27 wild-type (p27WT) overexpression reduced cell migration and invasion, while the overexpression of p27 lacking the nuclear localization signal (NLS) sequence promoted cell migration and invasion, and elevated Cofilin phosphorylation in MCF7 cells (Fig. S2A–C). There is no interaction between FMNL2 and Rac1 in breast cancer cells (Fig. S2D). In the previous study, we confirmed that FMNL2 reduced the levels of cytoplasmic p27 in breast cancer cells. Therefore, we further investigated whether cytoplasmic p27 was involved in FMNL2-mediated breast cancer cell migration and invasion. Compared to p27WT group, the cell migration and invasion, the Vimentin and Snail levels, and the RhoA/LIMK/Cofilin pathway were dramatically enhanced by p27△NLS overexpression in both FMNL2-overexpressing MCF7 and FMNL2-silencing MDA-MB-231 cells (Fig. 4A–E). These results demonstrated that cytoplasmic p27 could promote FMNL2-mediated cell migration and invasion through RhoA/LIMK/Cofilin pathway in breast cancer cells.

### The expression and prognosis of FMNL2 were associated with ER in breast cancer

**Fig. 5:**
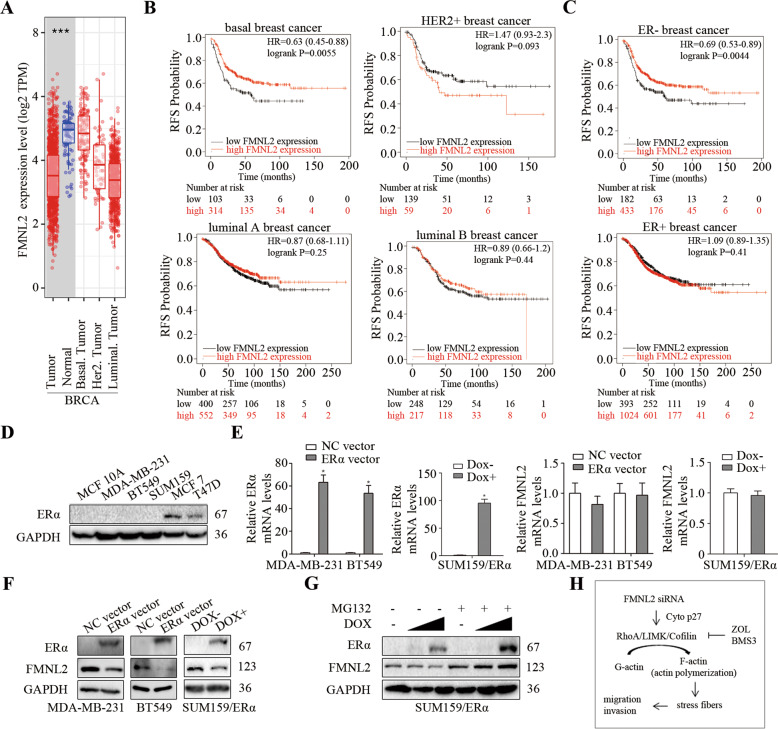
The expression and prognosis of FMNL2 were associated with ER in breast cancer. After transfection for 48 h, cultured cells were processed for indicated assays. **A** The expression of FMNL2 in different subtyping of breast cancer using the TIMER database. **B**, **C** The RFS probability analysis of FMNL2 was conducted in different subtyping of breast cancer using the Kaplan-Meier Plotter database. **D** The levels of ERα protein were measured using western blotting in MCF10A and human breast cancer cells; **E**, **F** The mRNA and protein levels of FMNL2 were examined in ERα-overexpressing MDA-MB-231, BT549 and SUM159 cells using qRT-PCR and western blotting. **G** Cultured cells were incubated with MG132 (5 μM) for 12 h, or DOX (10 ng/mL, 100 ng/mL) for 24 h as indicated, then the levels of FMNL2 and ERα were examined using western blotting. **H** Model showing FMNL2-mediated cell migration and invasion via a role of cytoplasmic p27 through the RhoA/LIMK/Cofilin pathway. *P < 0.05 *versus* corresponding control group.

TIMER analysis indicated that FMNL2 expression was negatively associated with ER-related molecular subtyping in breast cancer; FMNL2 was low-expressed in luminal subtyping, but highly expressed in basal subtyping of breast cancer tissues (Fig. 5A). Kaplan-Meier plotter analysis showed that the prognosis of FMNL2 was only statistically significant in ER-negative subtyping of breast cancer, and the low FMNL2 expression was significantly associated with poor recurrence-free survival (RFS) probability in ER-negative and basal subtyping of breast cancer (Fig. 5B, C). Additionally, FMNL2 was positively expressed in ER-negative MDA-MB-231, BT549, and SUM159 cells, and weakly expressed in ER-positive MCF7 and T47D cells (Fig. 5D). Moreover, ERα overexpression induced no changes on FMNL2 mRNA levels, but caused an evident decrease of FMNL2 protein in MDA-MB-231, BT549, and SUM159 cells (Fig. 5E, F). To further corroborate the above findings, we introduced the treatment with proteasome inhibitor MG132, and found that the degradation of FMNL2 protein caused by ERα overexpression could be effectively reversed in SUM159 cells (Fig. 5G). However, ERα downregulation could not be reversed by MG132 in FMNL2-overexpressing MCF7 cells (Fig. S3). Thus, these data indicated that the expression and prognosis of FMNL2 were associated with ER in breast cancer.

## Discussion

Previous studies have reported that FMNL2 overexpression is related to invasion and lymphatic metastasis in colorectal cancer [10, 22, 23]. FMNL2 upregulation is associated with poor outcome in term of RFS or disease-specific survival in melanoma [7]. However, despite that, the effects of FMNL2 on breast cancer cell migration and invasion have not been reported yet. In the current study, FMNL2 was downregulated in breast cancer cells when compared to non-cancerous MCF10A cells. Unlike those studies reporting the positive role of FMNL2 in several cancers, our results illustrated that FMNL2 silencing promoted cell migration and invasion of breast cancer in vitro and in vivo, suggesting that FMNL2 may function as a negative regulator of breast cancer migration and invasion. Critically, our findings are validated by the fact that decreased FMNL2 expression has been regarded as a sign of poor outcome in hepatocellular carcinoma [12]. DIAPH3 is decreased in breast cancer tissues, and DIAPH3 overexpression inhibits cell migration and invasion of triple-negative breast cancer by inhibiting RhoA-GTP expression [24]. Moreover, FMNL2 depletion resulted in decreased polarization and significantly higher incidence of impaired lumen formation in MCF10A cells [13]. We further demonstrated that FMNL2 silencing facilitated the actin cytoskeleton rearrangement in breast cancer cells that was necessary for cancer cell motility.

During cell mobility, remodeling actin undergoes dynamic assembly and disassembly and then mediates contractile filament and polarity changes, leading to promoted metastasis. It has been well established that once LIMK phosphorylation is occurred, its activation induces Cofilin phosphorylation on Ser3, resulting in Cofilin inactivation by blocking the actin-binding ability of Cofilin [25, 26]. Cofilin inactivation subsequently suppressed actin depolymerization activity and F-actin cleavage, and augmented actin stress fiber formation, therefore promoting tumor metastasis [16, 27]. Our data demonstrated that FMNL2 silencing promoted the phosphorylation of LIMK/Cofilin pathway, thus contributing to elevated F-actin polymerization and increased breast cancer cell migration and invasion. It is worth noting that Rho family plays a pivotal role in the formation of actin stress fiber and actin cytoskeleton remodeling of cell migration and tumor metastasis [28]. Moreover, formins act as effector proteins of Rho GTPases [29]. RhoA-directed formin mDia2 activity is essential for tight spheroid organization, and the mDia2/ROCK signaling mediates invasive egress from epithelial ovarian cancer spheroids [30]. Specifically, RhoA activation stimulates downstream effectors and mediates Cofilin-dependent actin cytoskeleton reorganization in lung cancer cell migration [31]. Indeed, the migration-promoting effects and the enhanced RhoA/LIMK/Cofilin pathway induced by FMNL2 silencing could be abrogated by ZOL or BMS3, highlighting the involvement of RhoA/LIMK/Cofilin pathway in FMNL2-mediated inhibition of breast cancer cell migration and invasion.

In our previous work, FMNL2 promoted cell proliferation by reducing p27 nuclear localization and p27 protein stability in breast cancer cells. Interestingly, without nucleocytoplasmic transport of p27, FMNL2 also decreased cytoplasmic p27 levels in breast cancer cells [14]. Nuclear p27 acts as tumor suppressor critical to enforce cell cycle arrest and inhibit cell proliferation. However, cytoplasmic p27 can act as an oncogene, and has been reported to be involved in Rho signaling, thereby inducing cytoskeletal rearrangement and cell motility [32]. CacyBP/SIP inhibits glioma cell migration and invasion through promoting the degradation of cytoplasmic p27 [33]. Here, our findings further indicated that cytoplasmic p27 promoted FMNL2-mediated cell migration and invasion through RhoA/LIMK/Cofilin pathway in MCF7 and MDA-MB-231 cells. Cytoplasmic p27 interacts directly or indirectly with actin-remodeling proteins and p27-dependent cell migration requires Ser10 phosphorylation and the p27 scatter domain (118-158 aa); Rac GTPase was also necessary for p27-dependent migration but alone was insufficient for HepG2 cell migration [34]. However, our data indicated that FMNL2 interacted with RhoA, other than Rac1 in breast cancer cells. Meanwhile, FMNL2 not only reduced the protein amount and activity levels of Cdc42, Rac1 and RhoA, but also repressed the RhoA/LIMK/Cofilin pathway in breast cancer cells, implying that FMNL2 regulated Rho activities and the downstream LIMK/Cofilin pathway in the process of breast cancer cell migration and invasion. Using Rho inhibitor ZOL and LIMK inhibitor BMS3, our data further confirmed that FMNL2 suppressed breast cancer cell migration and invasion by inhibiting RhoA/LIMK/Cofilin pathway through a reduction of cytoplasmic p27 (Fig. 5H). More study is needed to explore the underlying mechanisms involved in how cytoplasmic p27 mediates Rho activities, and how Cdc42 and Rac1 activities participate in FMNL2-mediated RhoA/LIMK/Cofilin pathway. We also speculated that p27 phosphorylation might be involved in the regulation of the cytoplasmic distribution of p27 in FMNL2-mediated breast cancer cell migration and invasion. Furthermore, consistent with previous studies [35, 36], transfection of Flag-p27△NLS vector induced the expression of a variant p27, which could migrate more slowly than the protein of Flag-p27WT and endogenous p27. It is speculated that this may be because p27△NLS protein cannot enter the nucleus and almost remains in the cytoplasm, resulting in certain post-translational modifications and higher molecular weight when compared to the p27WT protein. Further experiments are needed to explore the deep specific mechanisms. Additionally, as the targeted sequence of p27 small interfering RNA (siRNA) is located just in the 153-169 aa of NLS sequence of p27, p27 siRNA has no targeted silencing efficiency against p27△NLS. It may be due to the high-efficiency silencing of p27, the endogenous p27 protein was almost not detectable after sip27 transfection, and the overexpression efficiency of p27WT is quite weak in MCF7 cells.

Although the role of FMNL2 in various cancer metastasis and progression is still controversial, herein, our results demonstrated that FMNL2 was the potential of suppressing breast cancer metastasis, at least. Particularly, breast cancer is a heterogeneous disease that possesses diverse prognostic outcomes and involves the expression of hormone receptor including ER. Our previous clinical data has revealed that FMNL2 expression is negatively correlated with ER in breast cancer. It has been mentioned that FMNL2 has a stronger correlation with ER in the co-expression networking via an integrated systematic approach [37]. We further found that ERα might be a negative regulator for FMNL2 at the posttranslational level in breast cancer, implying that FMNL2 expression partially depended on ER status. These findings may account for why FMNL2 expressed differently in various breast cancer cell types; at least, compared to ER-positive MCF7 and T47D cells, FMNL2 expression was relatively high in ER-negative MDA-MB-231, BT549, and SUM159 cells. Moreover, ERα negatively regulated FMNL2 protein levels via a proteasome-related pathway. Further thorough and expanded research are needed to deeply explore the concrete molecular regulatory mechanisms concerning how ER affects FMNL2 expression in breast cancer.

Combined with the analysis from TCGA and Kaplan-Meier plotter databases, it was reasonable that as mentioned earlier, FMNL2 silencing promoted migration and invasion of ER-negative MDA-MB-231 and BT549 cells, FMNL2 downregulation was significantly associated with poorer RFS probability in ER-negative and basal subtyping of breast cancer. This was probably because compared with cell proliferation, metastasis is the most important factor determining the prognosis of advanced breast cancer patients. Moreover, RhoA/ROCK1/LIMK/Cofilin signaling pathway has been reported to induce motility-related changes in the actin cytoskeleton and cell migration of endometrial cancer [38]. ROCK/LIMK/Cofilin signaling proteins could be good candidates to develop cancer prevention strategies or therapeutics [39]. Taken together, we may conclude that FMNL2 suppressed migration and invasion of breast cancer cells by inhibiting RhoA/LIMK/Cofilin pathway through a reduction of cytoplasmic p27. This finding implies that the interference of FMNL2-mediated RhoA/LIMK/Cofilin pathway involving the cytoplasmic p27 could be a promising therapeutic strategy for ameliorating breast cancer metastasis and prognosis.

## Materials and methods

### Cell culture

All human breast cancer cell lines were purchased from Shanghai Institute of Biochemistry and Cell Biology (Chinese Academy of Sciences, Shanghai, China), and authenticated by short tandem repeat (STR) DNA fingerprinting and tested for mycoplasma contamination. The MDA-MB-231 and MCF7 cells were cultured in DMEM Medium (Hyclone, UT, USA), T47D and BT549 cells were cultured in RPMI-1640 Medium (Hyclone), MCF10A and SUM159 cells were cultured in DMEM/F12 medium (Hyclone), with all recommended supplements, respectively. All cultures were maintained at 37 °C in a humidified incubator with 5% CO_2_.

### Reagents

ZOL was kindly provided by Novartis Pharmaceuticals (East Hanover, NJ, USA). ZOL as Rho inhibitor, BMS3 (MedChemExpress, Monmouth, NJ, USA) as LIMK inhibitor, MG132 (Sigma, St Louis, MO, USA) as proteasome inhibitor, and doxycycline (DOX; Yeasen Biotechnology, Shanghai, China) as gene expression inducer were used in cell treatment.

### siRNA, plasmids, and lentiviral infection

Cultured cells were transfected with corresponding siRNAs and plasmids using Lipofectamine 2000 (Invitrogen, Carlsbad, CA, USA). The siRNA sequences for human FMNL2 (siFMNL2-1 and siFMNL2-2), p27 (sip27), and the negative control siRNA (NC siRNA) were synthesized (GenePharma Biotechnology, Shanghai, China) and shown in Table S1. ERα plasmids were purchased from GeneChem (Shanghai, China). The pCMV-N-Flag-p27WT and pCMV-N-Flag-p27△NLS plasmids were constructed as described previously [14]. The lentiviral shRNA-negative control (Lv-shNC) and shRNA-FMNL2 (Lv-shFMNL2) viruses were constructed using the above siRNA sequences. MDA-MB-231 cells were infected with these viruses and then selected by G418.

### Quantitative real-time reverse transcription-polymerase chain reaction (qRT-PCR) assay

Total RNA was extracted using the RNAfast 200 kit (Fastagen Biotechnology, Shanghai, China), and cDNA was synthesized with PrimeScript RT Master Mix (Takara Biotechnology, Dalian, China). The qRT-PCR was conducted with TB Green Premix Ex Taq II (Takara Biotechnology). The primers were synthesized (Sangon Biotechnology, Shanghai, China) and listed in Table S2.

### Western blotting

The co-immunoprecipitation (Co-IP) assay was conducted by a Dynabeads protein G immunoprecipitation kit (Invitrogen). The primary antibodies used were anti-FMNL2 (390298), anti-Cdc42 (8401), anti-p27 (528), anti-ERα (543) and anti-RhoA (418) antibodies (Santa Cruz Biotechnology, Santa Cruz, CA, USA); anti-Rac1 (2465), anti-phosphor-LIMK1/2 (3841) and anti-phosphor-Cofilin (3313) antibodies (Cell signaling, Beverly, MA, USA); anti-Flag (F1804) antibody (Sigma, St Louis, MO, USA); anti-Snail (53519) and anti-ERα (32063) antibodies (Abcam, Cambridge, MA, USA); anti-Snail (27293) antibody (Novus Biologicals, Littleton, CO, USA); anti-Vimentin (10366-1), anti-LIMK1 (19699-1), anti-Cofilin (66057-1), anti-ROCK1 (21850-1) and anti-GAPDH (60004) antibodies (Proteintech, Wuhan, China). The immunoreactive bands were visualized by an enhanced chemiluminescence system.

### RhoA, Cdc42 and Rac1 activity assay

After transfection for 48 h, cells were processed for RhoA, Cdc42 and Rac1 activity according to the manufacturer’s instructions (Shanghai yiyan Biotechnology, China). The absorbance was determined at 450 nm using a Benchmark microplate reader (Bio-Rad, Hercules, CA, USA).

### Data processing

We retrieved data from Tumor Immune Estimation Resource (TIMER) (https://cistrome.shinyapps.io/timer/) and Kaplan-Meier plotter (http://www.kmplot.com/analysis/) to analyze the expression and prognosis of FMNL2 in breast cancer.

### Immunofluorescence assay

Briefly, cells were fixed, permeabilized and then blocked with 5% bovine serum albumin. Tetramethylrhodamine (TRITC)-phalloidin labelled F-actin was used for primary antibody incubation. Incubation with secondary antibodies was followed by 4’,6-diamidino-2-phenylindole (DAPI) staining. Images were taken by a Leica DMi8 Microscope (Wetzlar, Germany).

### Transwell assay

A transwell system with an 8 μm pore size (Corning, Lowell, MA, USA) was introduced to determine cell migration and invasion. For invasion assay, the transwell membrane was pre-coated with 100 μL matrigel (BD Biosciences, San Jose, CA, USA) in advance. For migration assay, cells were directly inoculated onto the upper chamber without FBS, while medium containing 10% FBS was added to the lower chamber.

### In vivo lung metastasis assay

Animals were allocated to the experimental and control group according to a random number table and no blinding was done. All animal experimental procedures were approved by the Committee of Institutional Animal Care and Use of Xi’an Jiaotong University. The female 4-week-old SCID/beige mice were purchased from Laboratory Animal Center of Xi’an Jiaotong University. Cultured cells (1 × 10^6^) were injected into the tail vein of mice (*n* = 6 per group). After scarification, lung tissues were fixed in 10% formalin, dehydrated, transparent, embedded in paraffin, and then stained following a standard hematoxylin and eosin staining protocol.

### Immunohistochemistry (IHC) assay

IHC staining was conducted using a Biotin-Streptavidin HRP Detection Systems (ZSGB-BIO, Beijing, China). Primary antibodies against Vimentin (Proteintech), and Snail (Abcam) were applied. Images were taken by a Leica SCN400 slide scanner (Germany).

### Statistical analysis

The sample size was chosen according to previous observations, which perform similar experiments to see significant results, or the results from our preliminary experiments. Each experiment was repeated three times. For animal studies, the sample size was estimated to be no <5 in seeding or hematogenous metastasis models. Statistical analysis was performed using GraphPad Prism version 5.0 software (GraphPad Software Inc., La Jolla, CA, USA) and SPSS version 20.0 (SPSS, Chicago, IL, USA). Data were expressed as mean ± standard deviation (SD). Comparison between two independent groups was made by the Student’s *t*-test, whereas the differences among multiple groups were analyzed by one-way analysis of variance (ANOVA) and post-hoc analysis. Multiple comparisons were done after homogeneity test for variance. The variance was similar between the groups that are being statistically compared. *P* < 0.05 was considered as statistically significant.

## Supplementary information

Original Data Files

Supplementary information

Figure S1

Figure S2

Figure S3

Table S1

Table S2

aj-checklist

## Data Availability

The datasets used and/or analyzed in this study are available from the corresponding author on reasonable request.
