# Supplementary material for: FMNL2 suppresses cell migration and invasion of breast cancer: a reduction of cytoplasmic p27 via RhoA/LIMK/Cofilin pathway
Source: Cell Death Discov. 2022 Apr 4;8:155. doi: 10.1038/s41420-022-00964-z (PMC8980084; doi:10.1038/s41420-022-00964-z)
Supplement: Supplementary file 1 — Original Data Files [file 41420_2022_964_MOESM1_ESM.pdf]

Below you will see the original images:

Fig. 1A

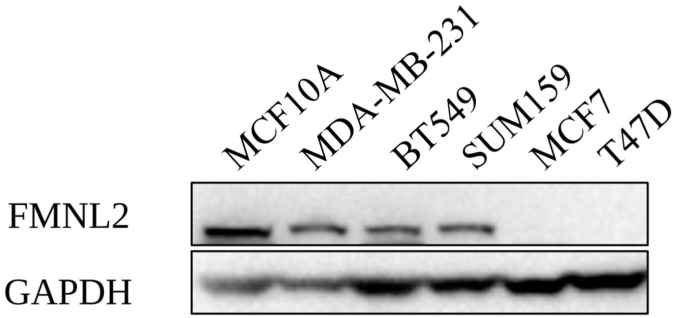

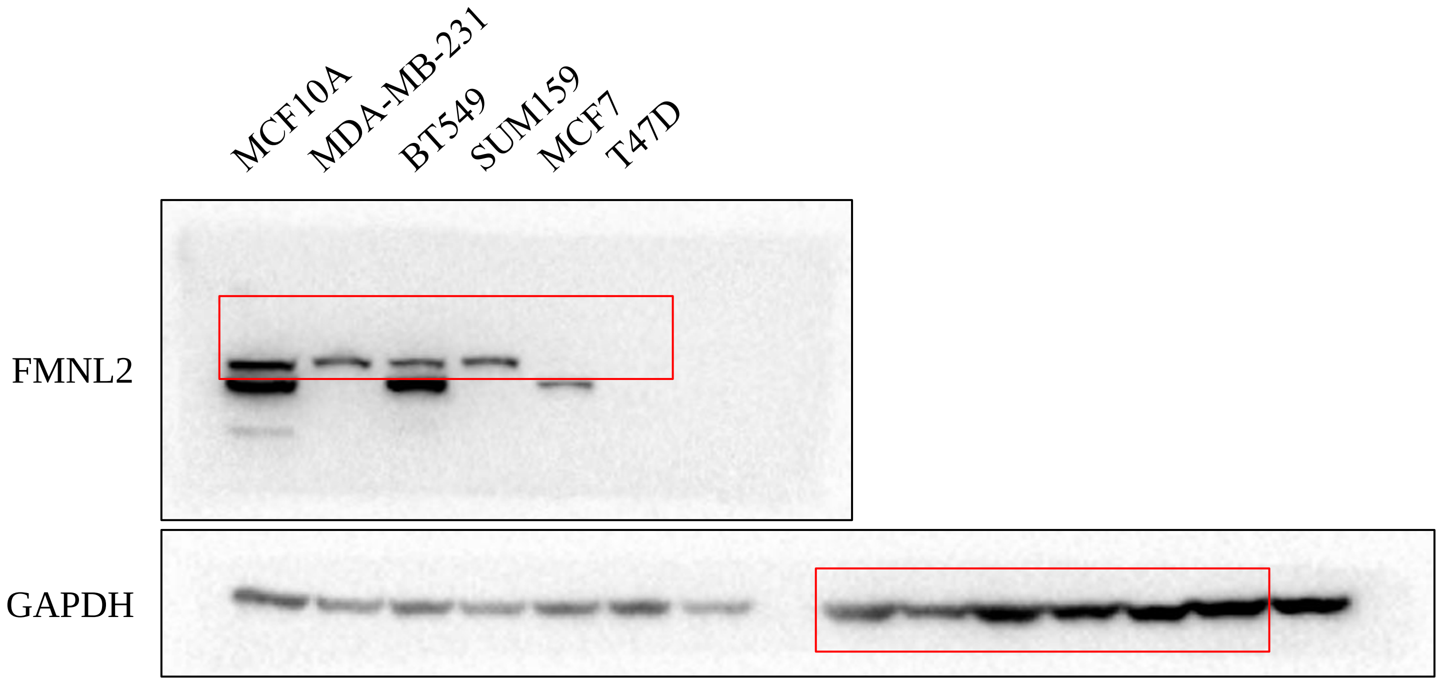

Fig. 1B

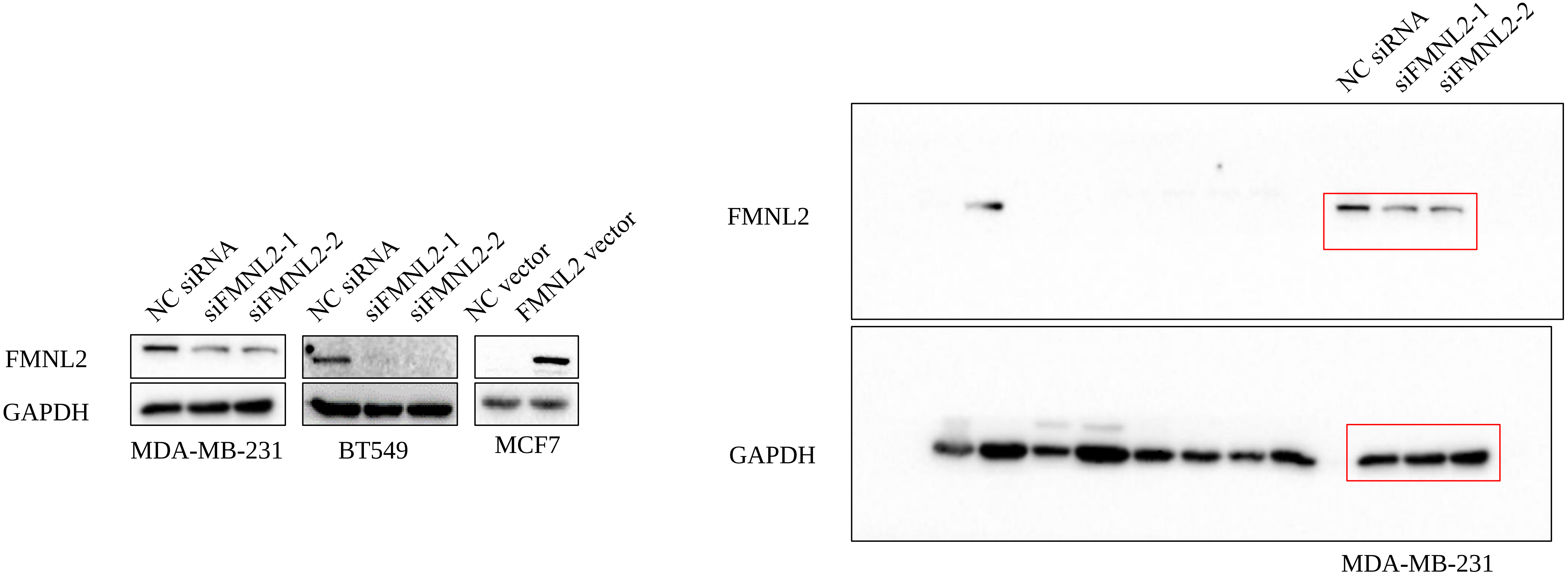
NC siRNA
siFMNL2-1
siFMNL2-2
NC siRNA
siFMNL2-1
siFMNL2-2
NC vector
FMNL2 vector
FMNL2
GAPDH
MDA-MB-231
BT549
MCF7
NC siRNA
siFMNL2-1
siFMNL2-2
FMNL2
GAPDH
MDA-MB-231

Fig. 1B

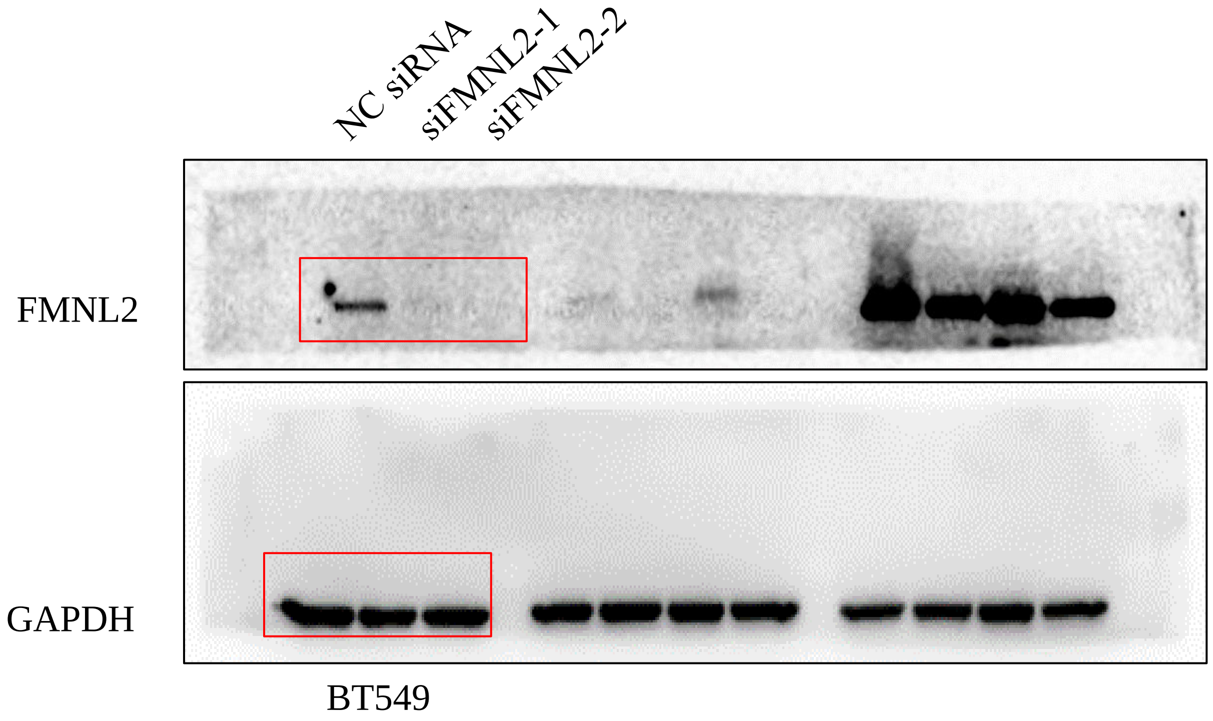
NC siRNA
siFMNL2-1
siFMNL2-2
FMNL2
GAPDH
BT549

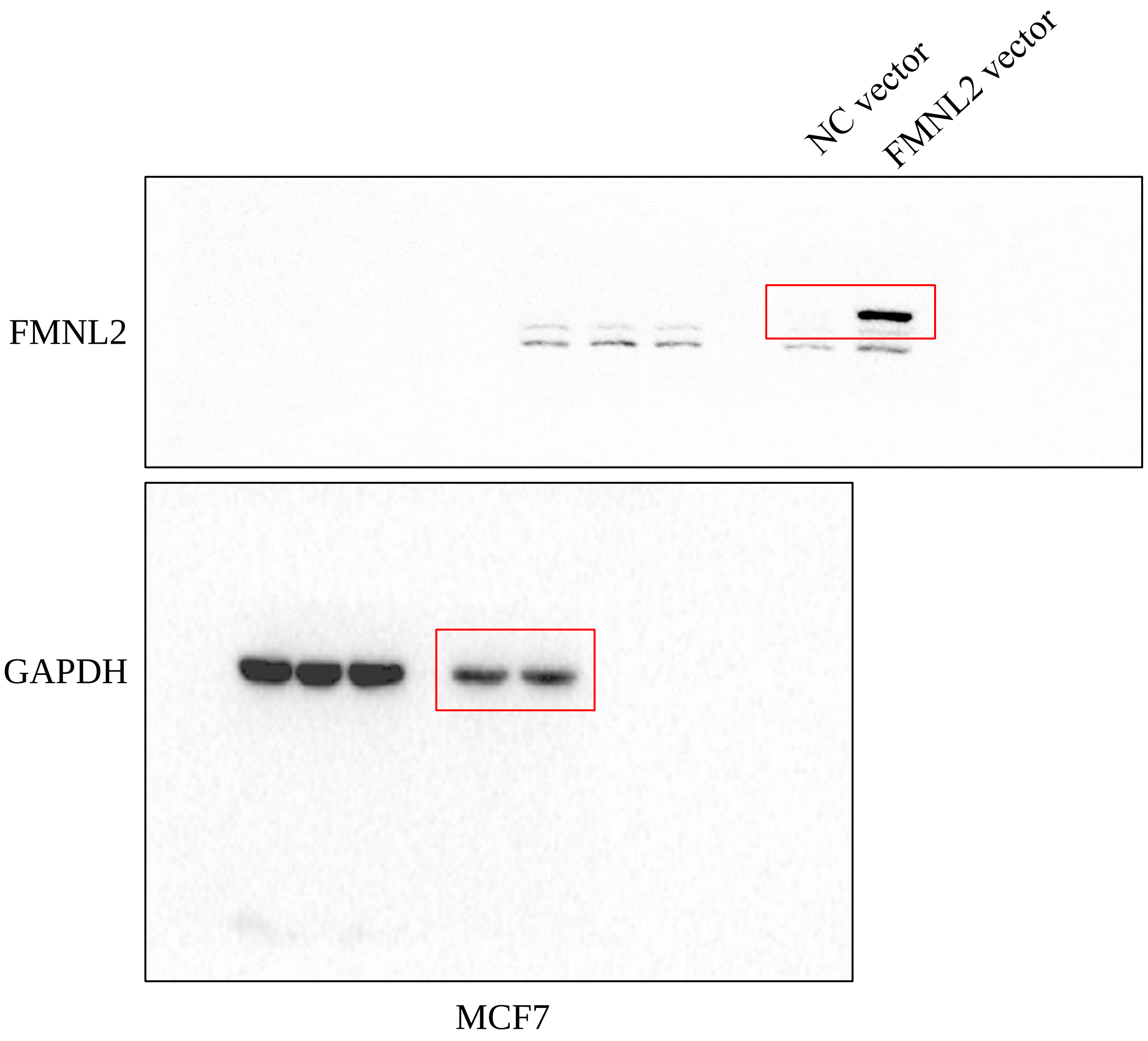
NC vector
FMNL2 vector
FMNL2
GAPDH
MCF7

Fig. 1C

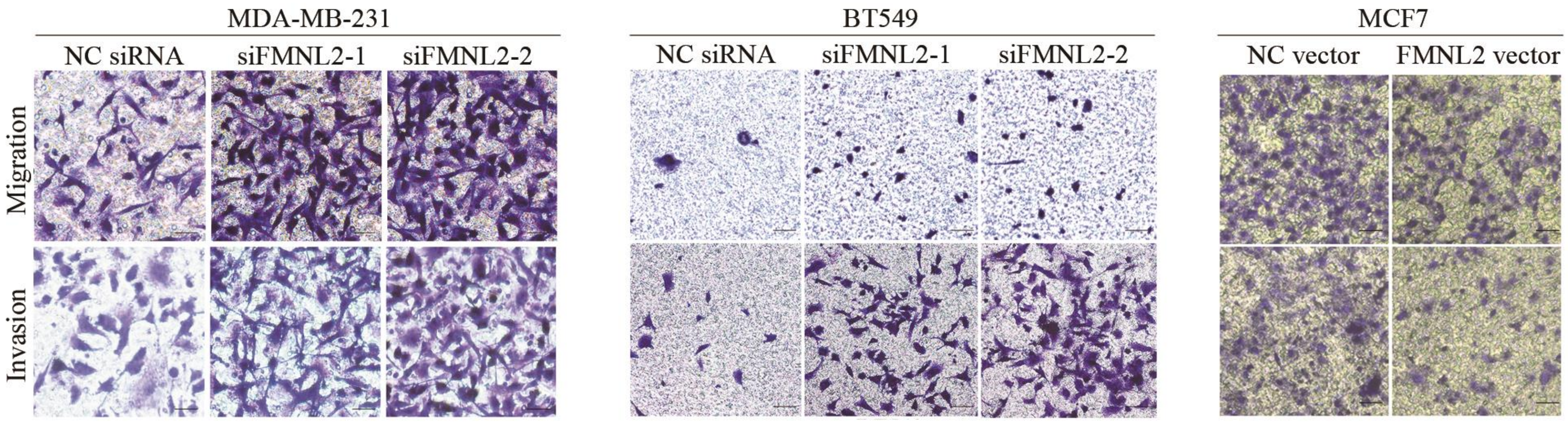
MDA-MB-231
NC siRNA
siFMNL2-1
siFMNL2-2
BT549
NC siRNA
siFMNL2-1
siFMNL2-2
MCF7
NC vector
FMNL2 vector
Migration
Invasion

Fig. 1C

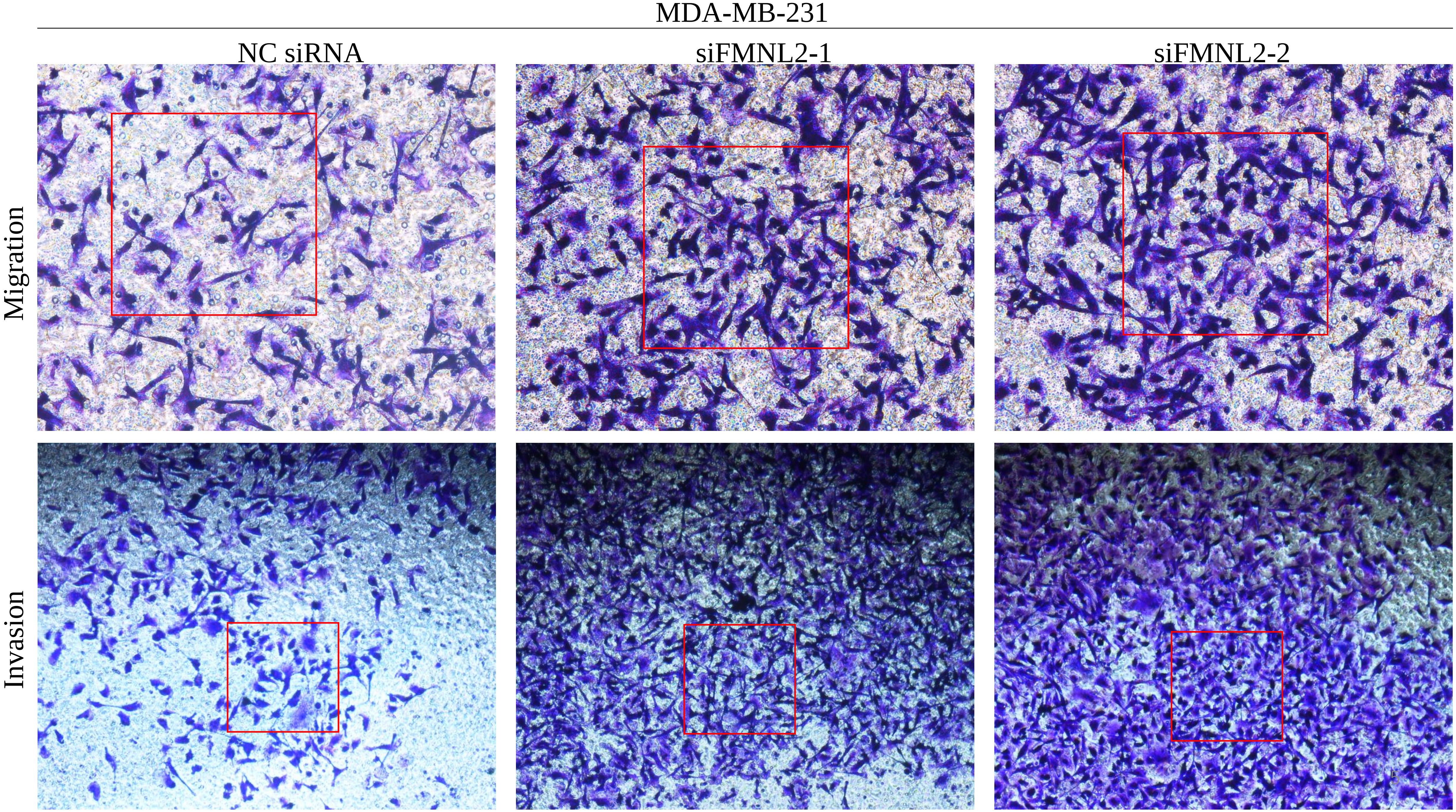

Fig. 1C

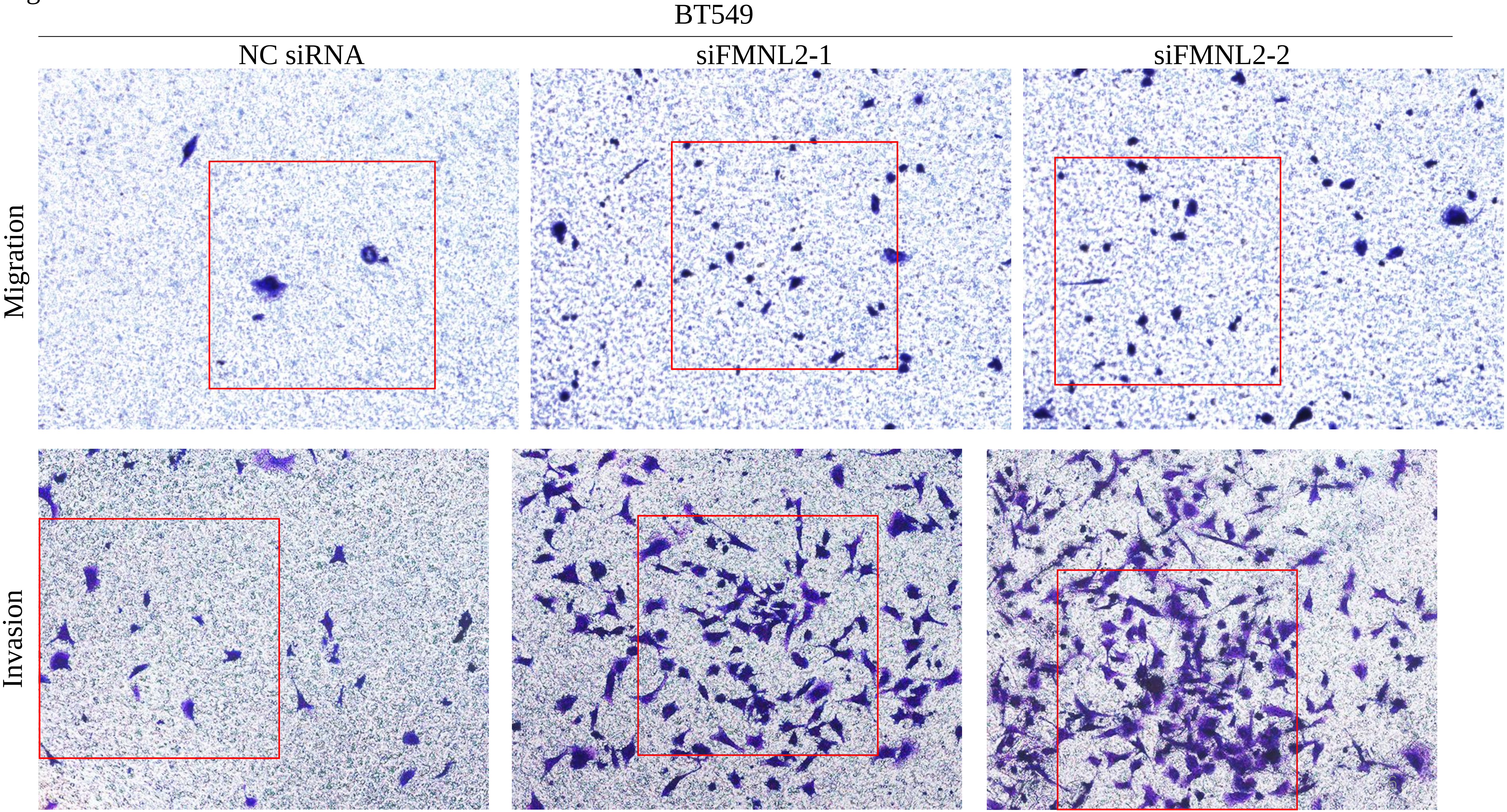

Fig. 1C

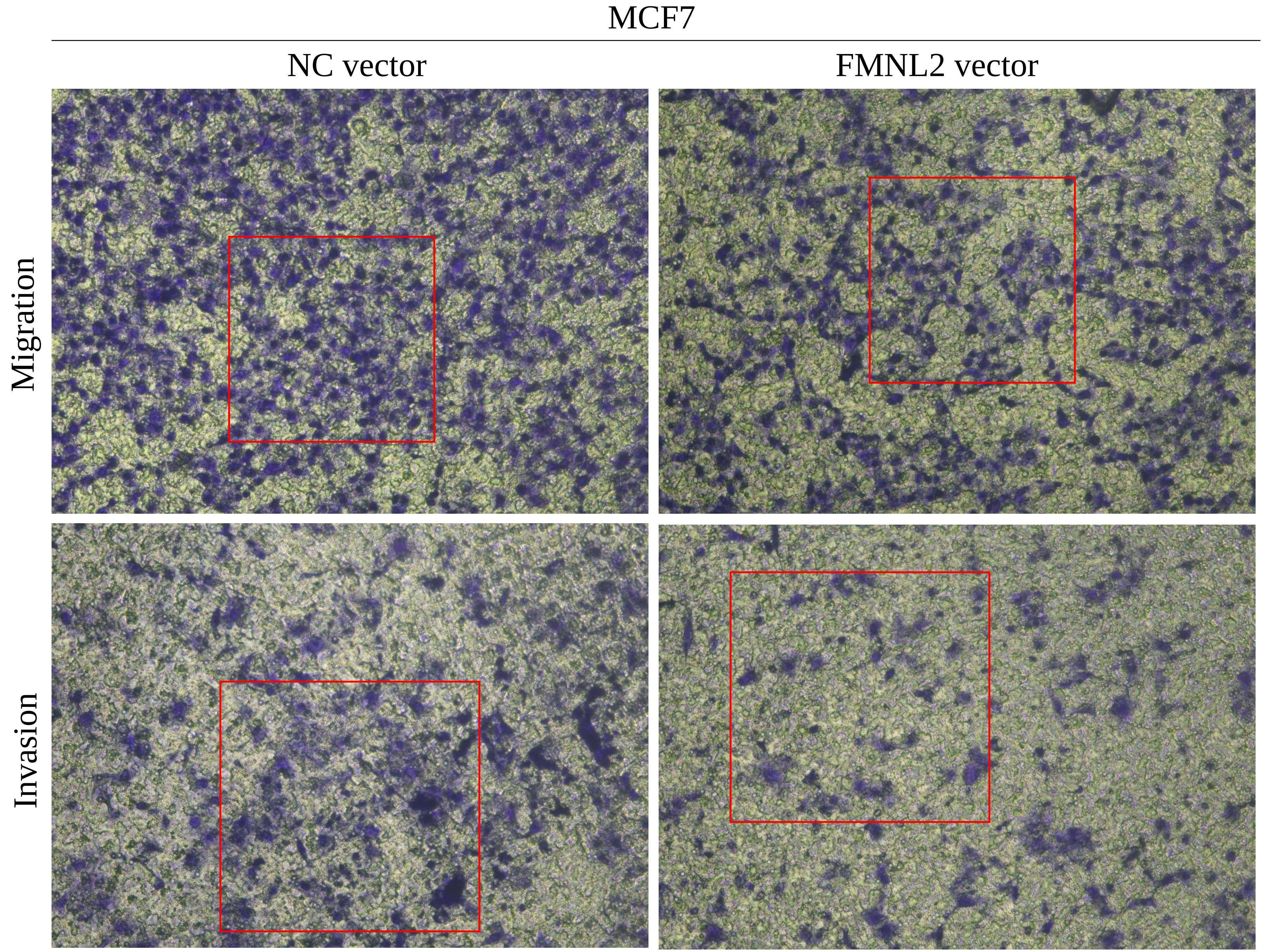

Fig. 1E

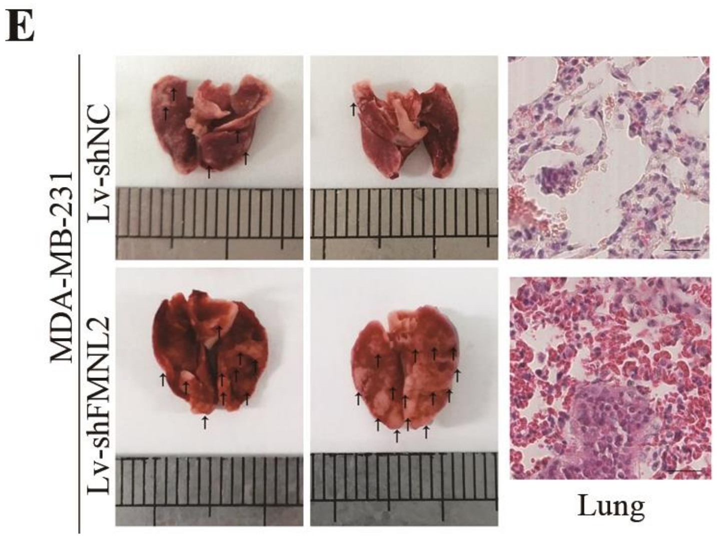

Fig. 1E

HE, Lv-shNC

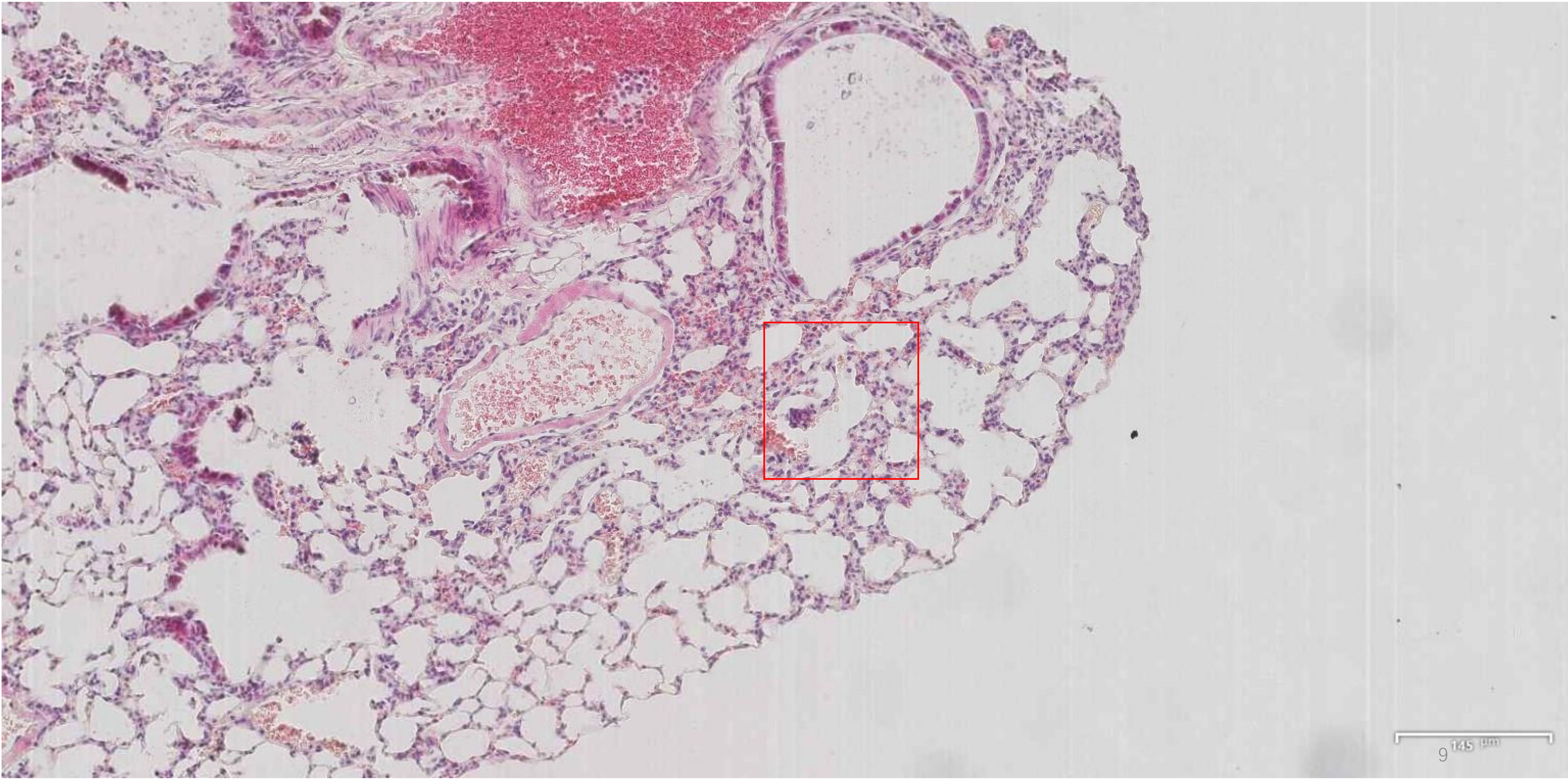

Fig. 1E

HE, Lv-shFMNL2

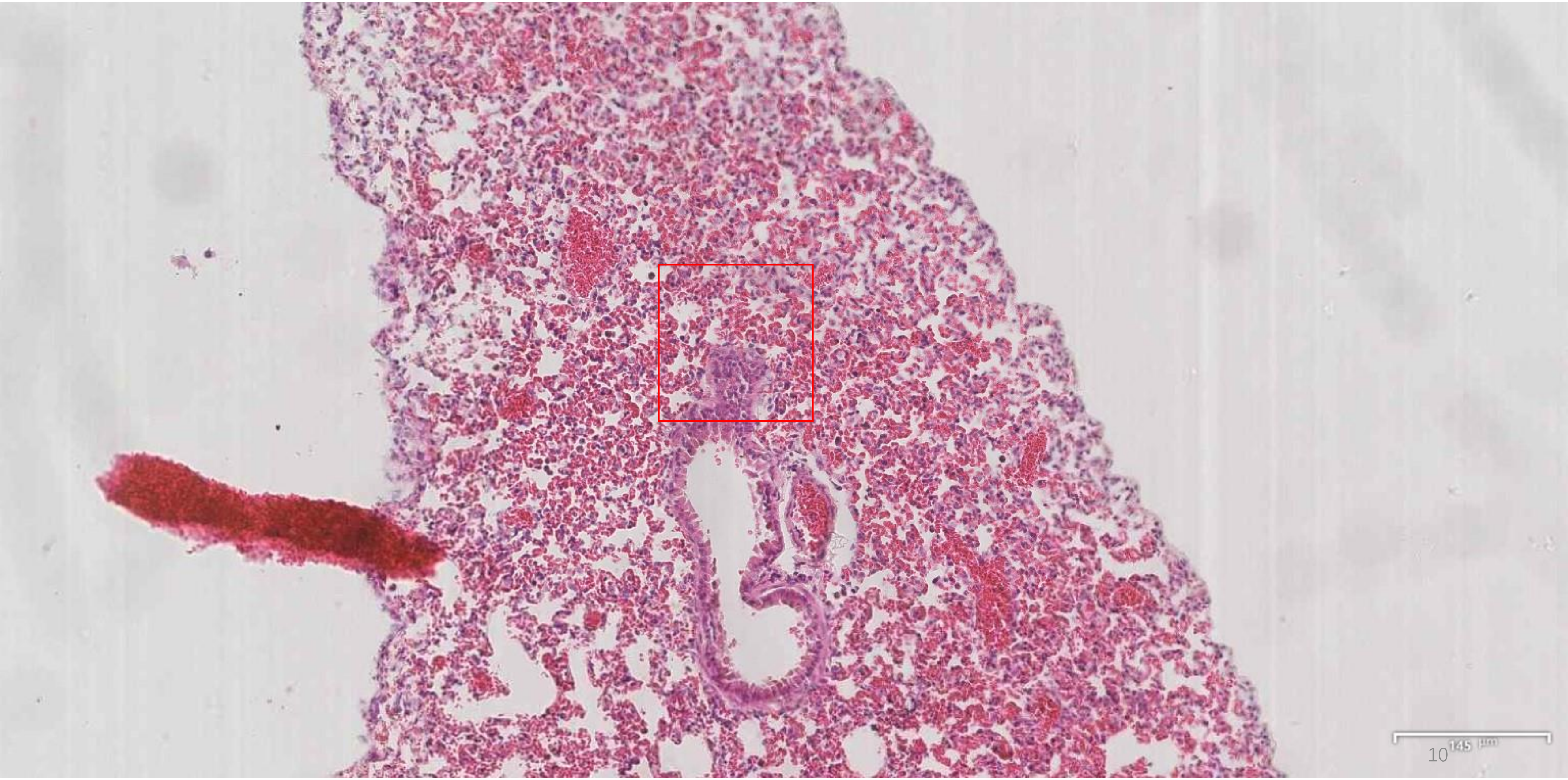

Fig. 1G

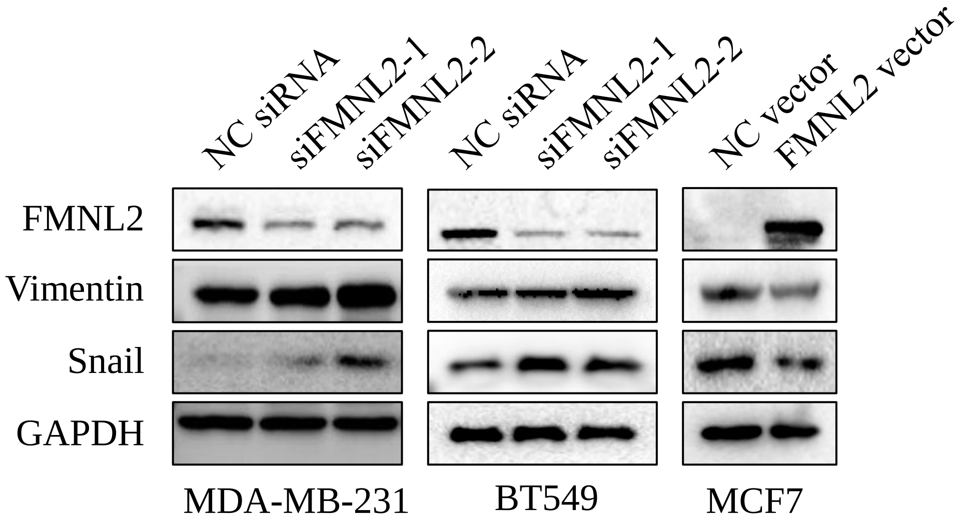
NC siRNA
siFMNL2-1
siFMNL2-2
NC siRNA
siFMNL2-1
siFMNL2-2
NC vector
FMNL2 vector
FMNL2
Vimentin
Snail
GAPDH
MDA-MB-231
BT549
MCF7

Fig. 1G

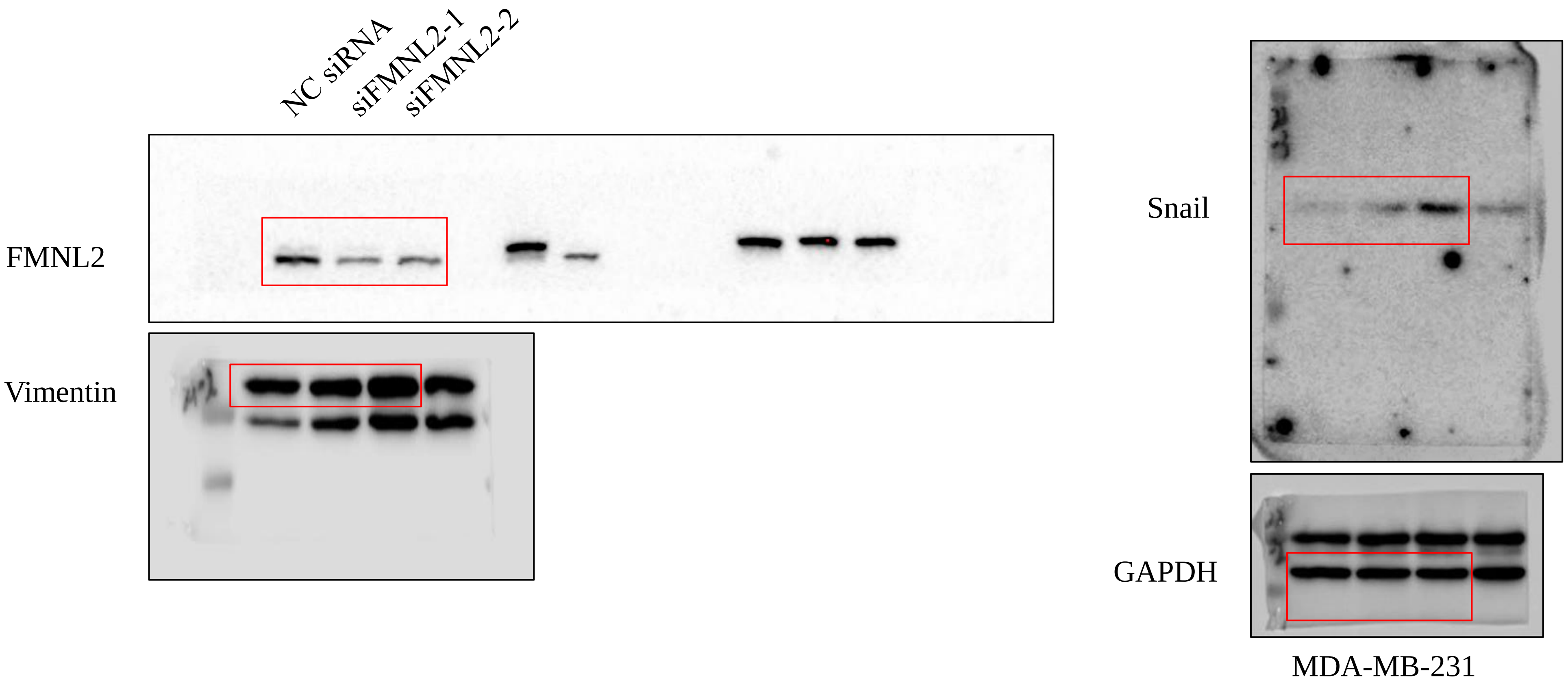
NC siRNA
siFMNL2-1
siFMNL2-2
FMNL2
Vimentin
Snail
GAPDH
MDA-MB-231

Fig. 1G

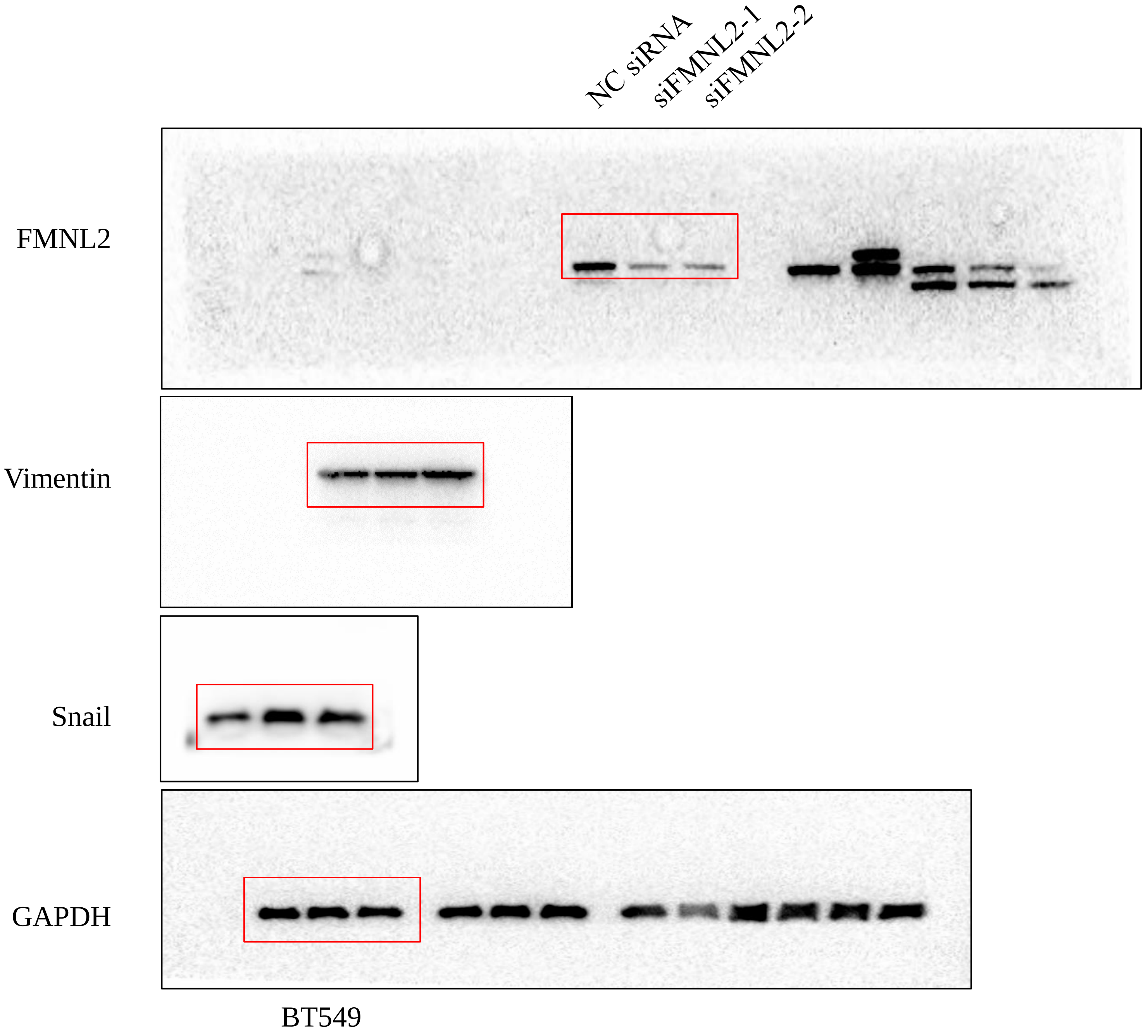
NC siRNA
siFMNL2-1
siFMNL2-2
FMNL2
Vimentin
Snail
GAPDH
BT549

Fig. 1G

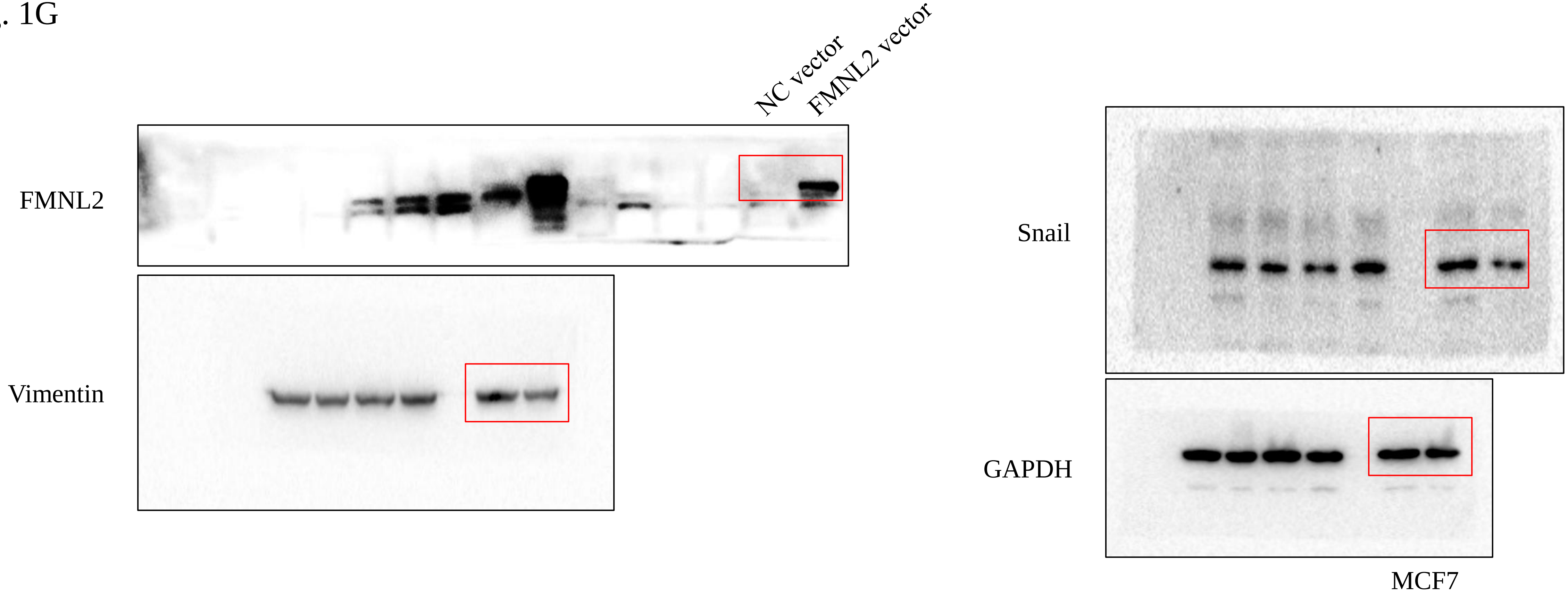

Fig. 1H

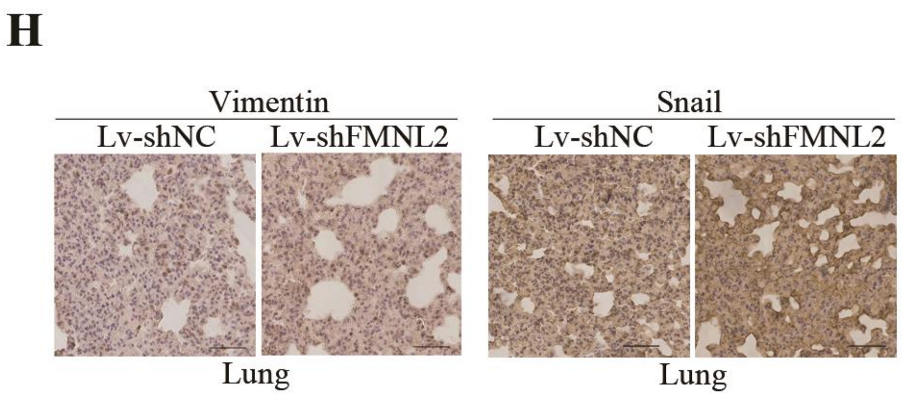
H
Vimentin
Lv-shNC
Lv-shFMNL2
Lung
Snail
Lv-shNC
Lv-shFMNL2
Lung

Fig. 1H

Vimentin, Lv-shNC

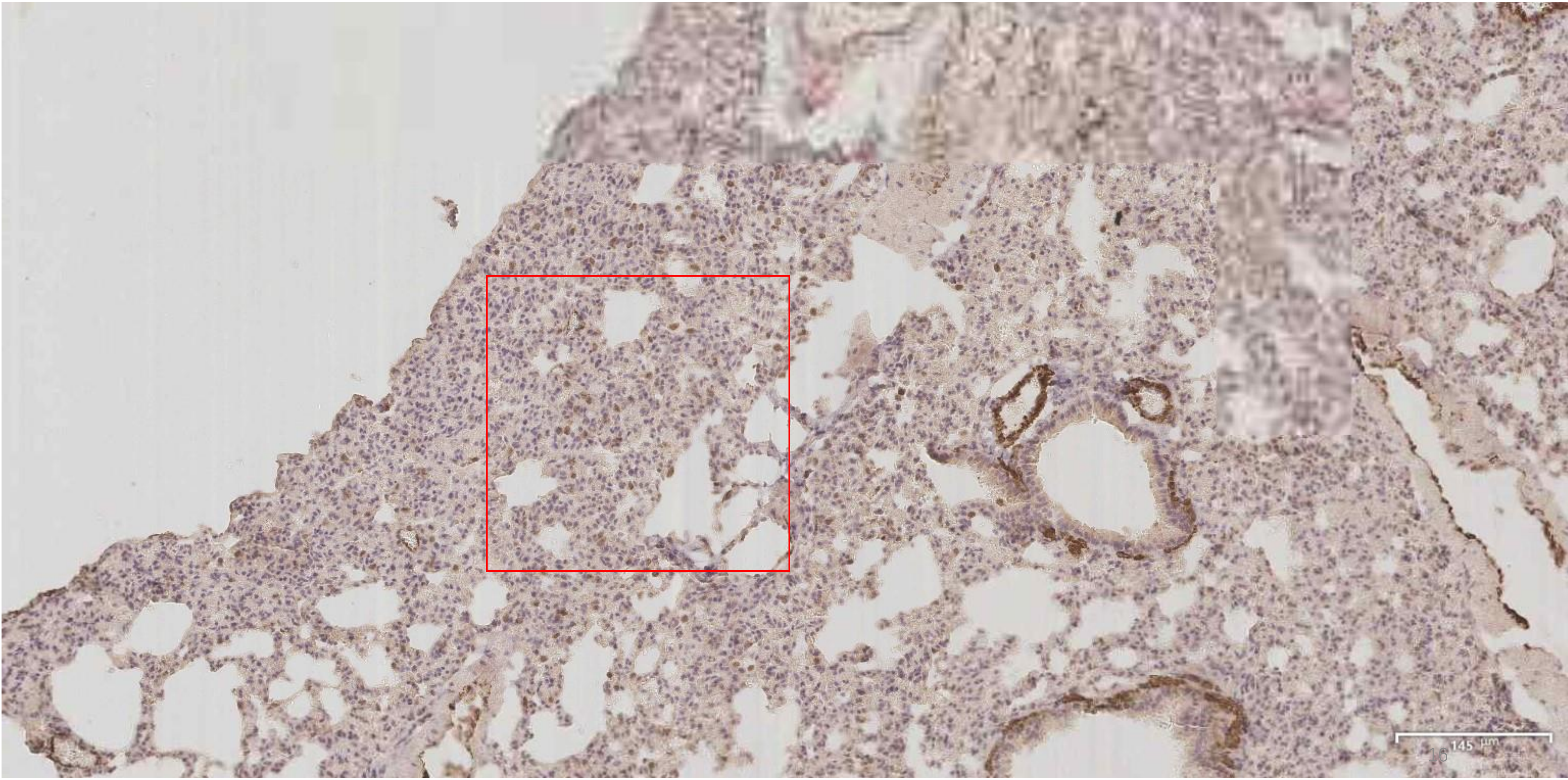

Fig. 1H

Vimentin, Lv-shFMNL2

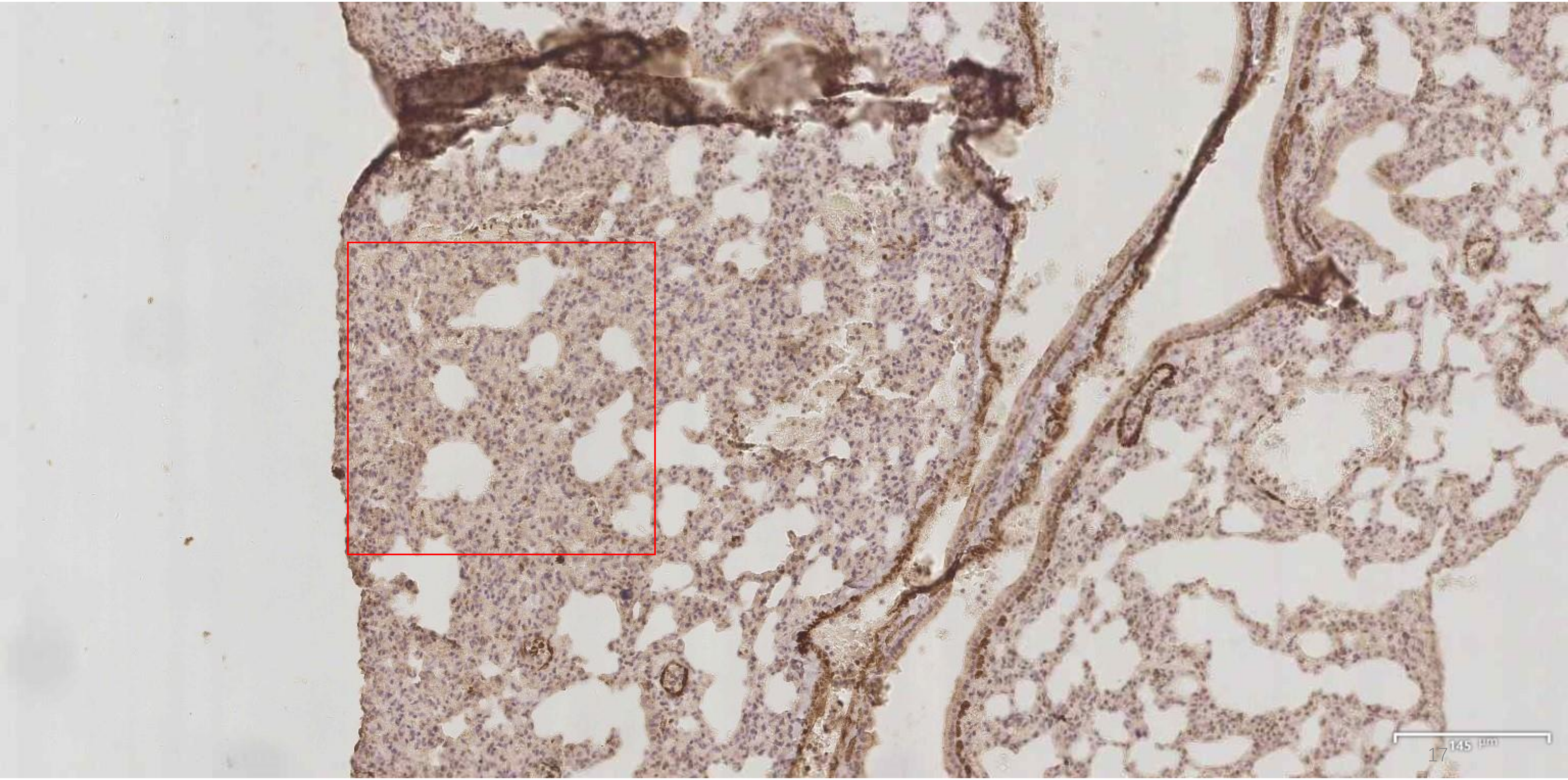

Fig. 1H

Snail, Lv-shNC

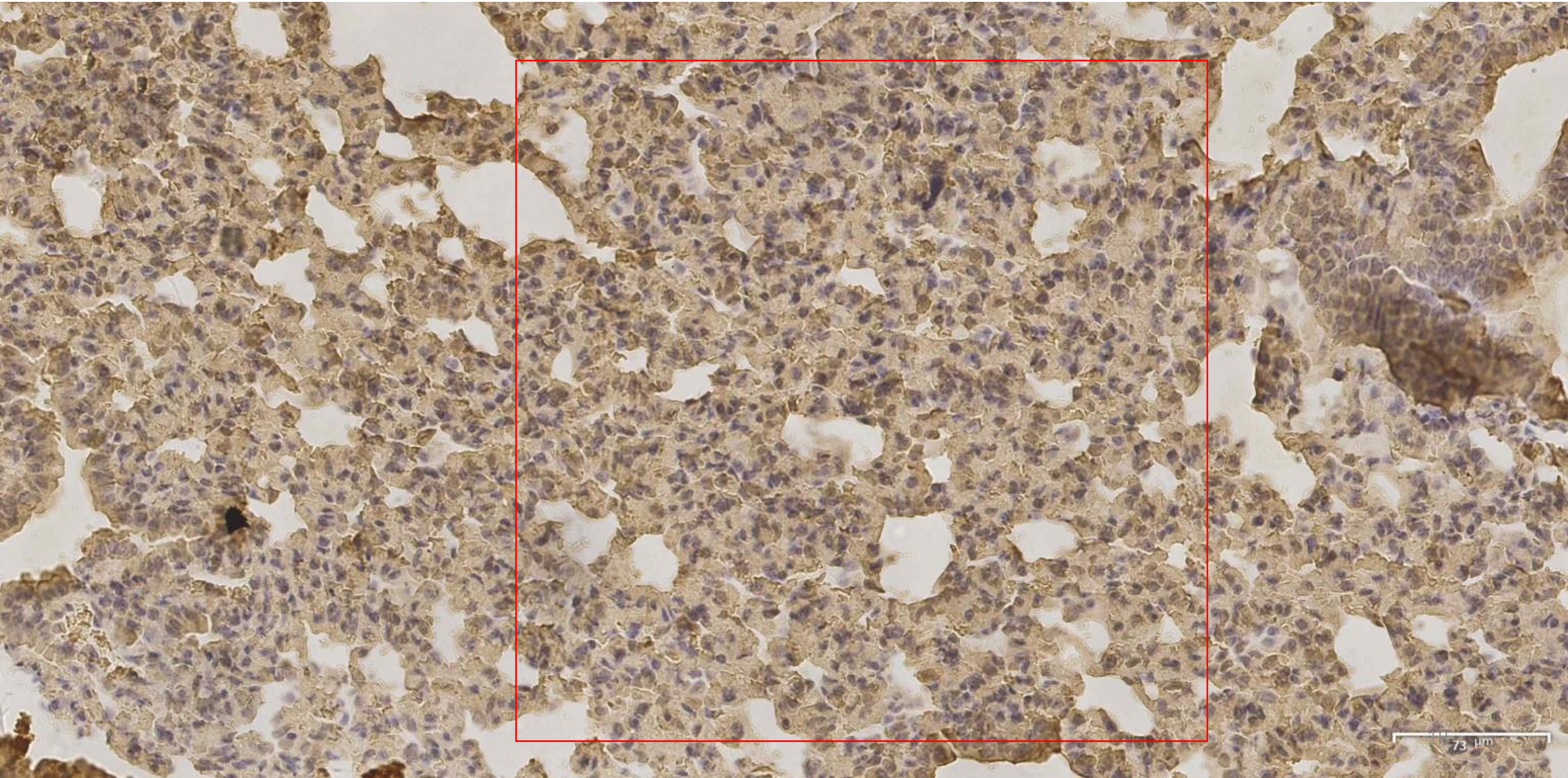

Fig. 1H

Snail, Lv-shFMNL2

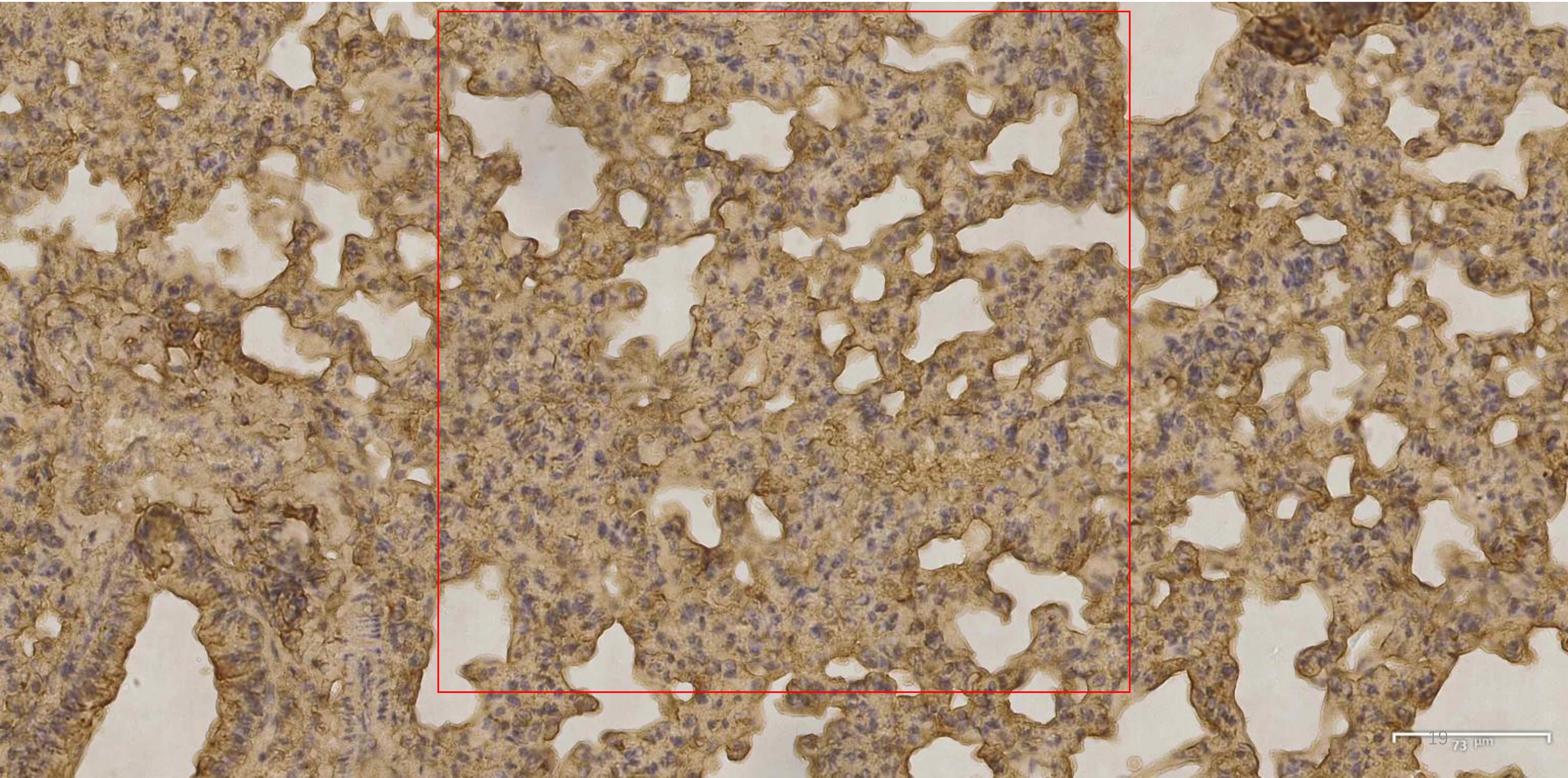

Fig. 2A

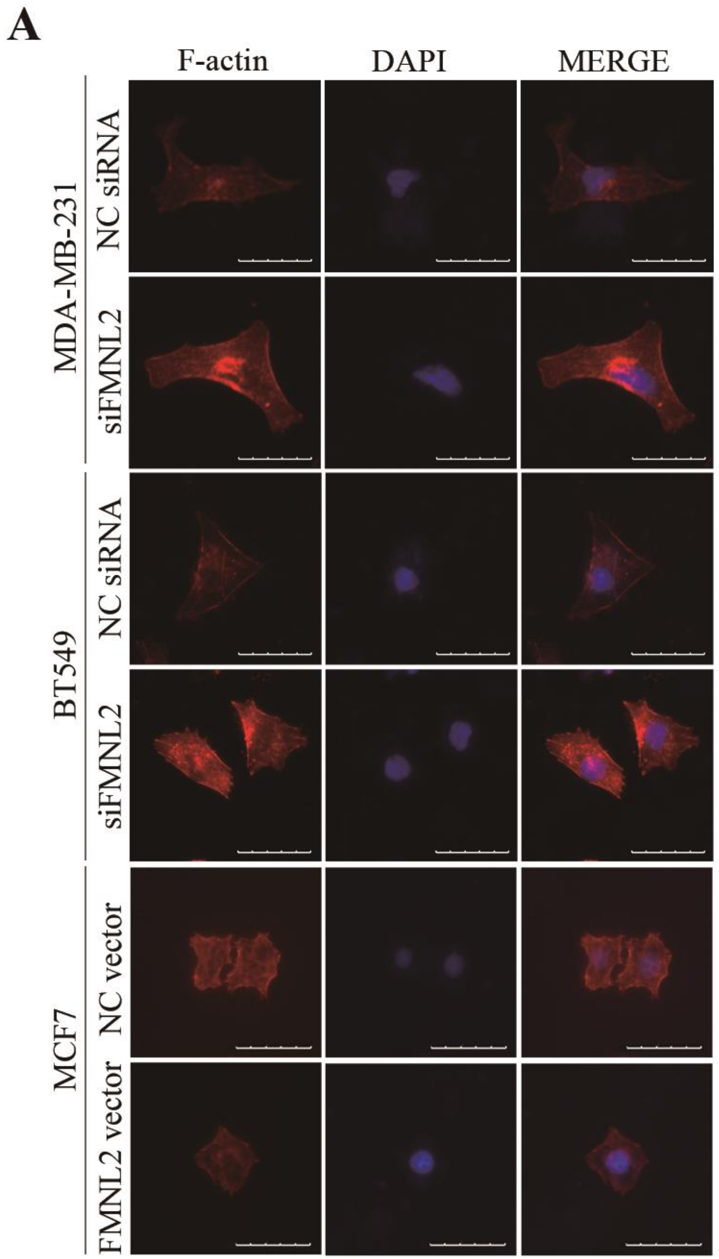
A
F-actin
DAPI
MERGE
MDA-MB-231
NC siRNA
siFMNL2
BT549
NC siRNA
siFMNL2
MCF7
NC vector
FMNL2 vector

Fig. 2A

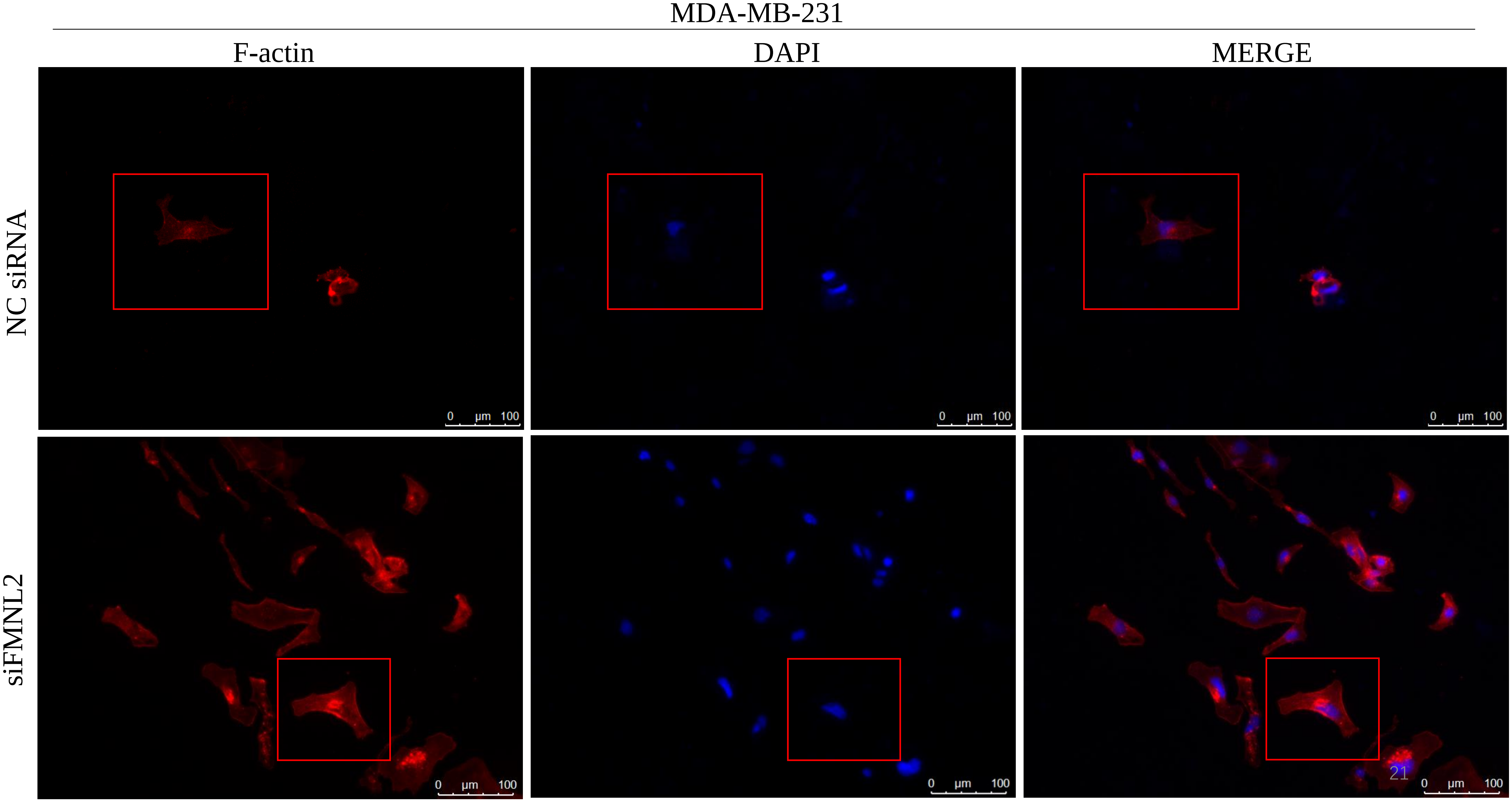

Fig. 2A

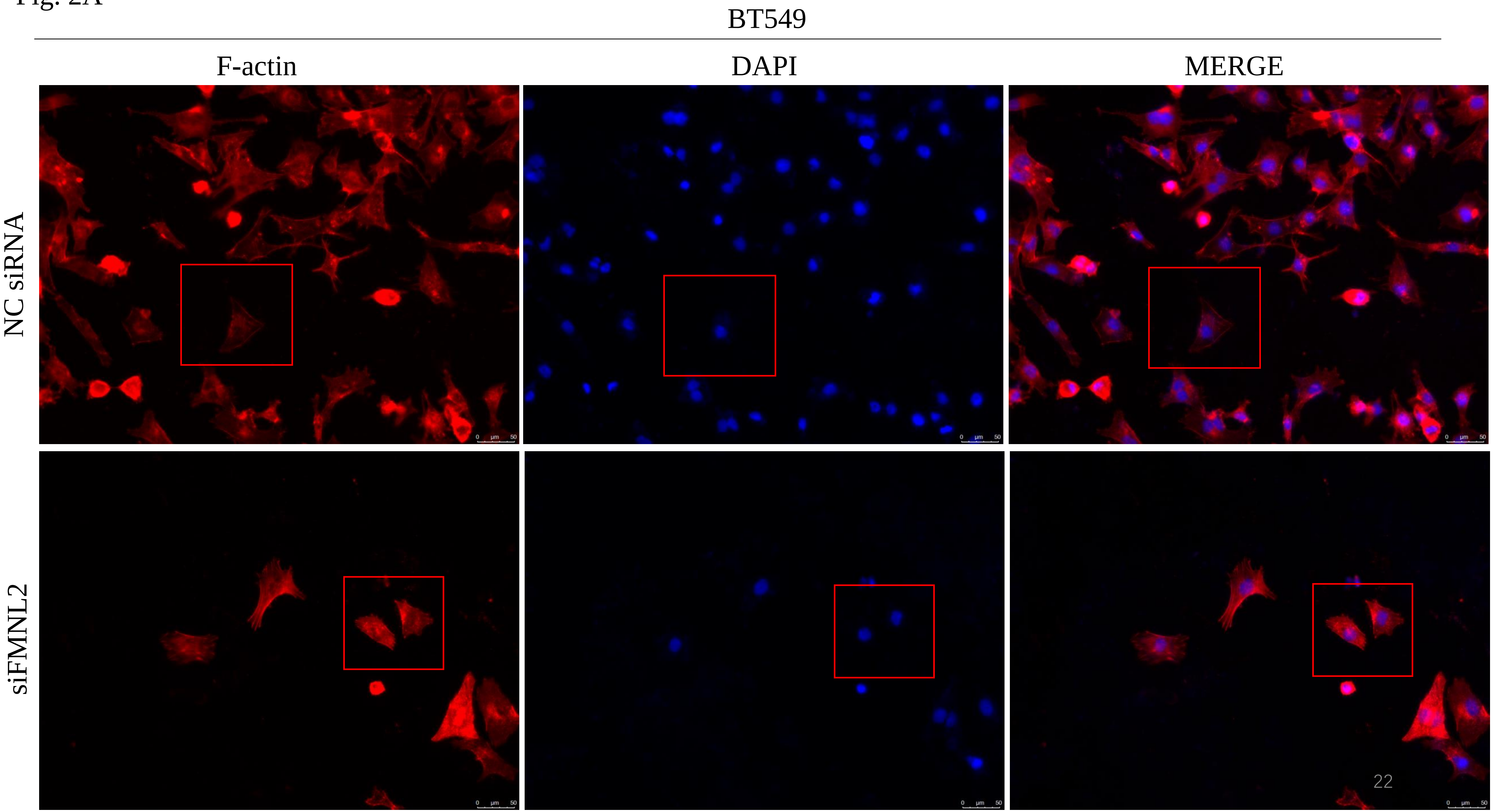

Fig. 2A

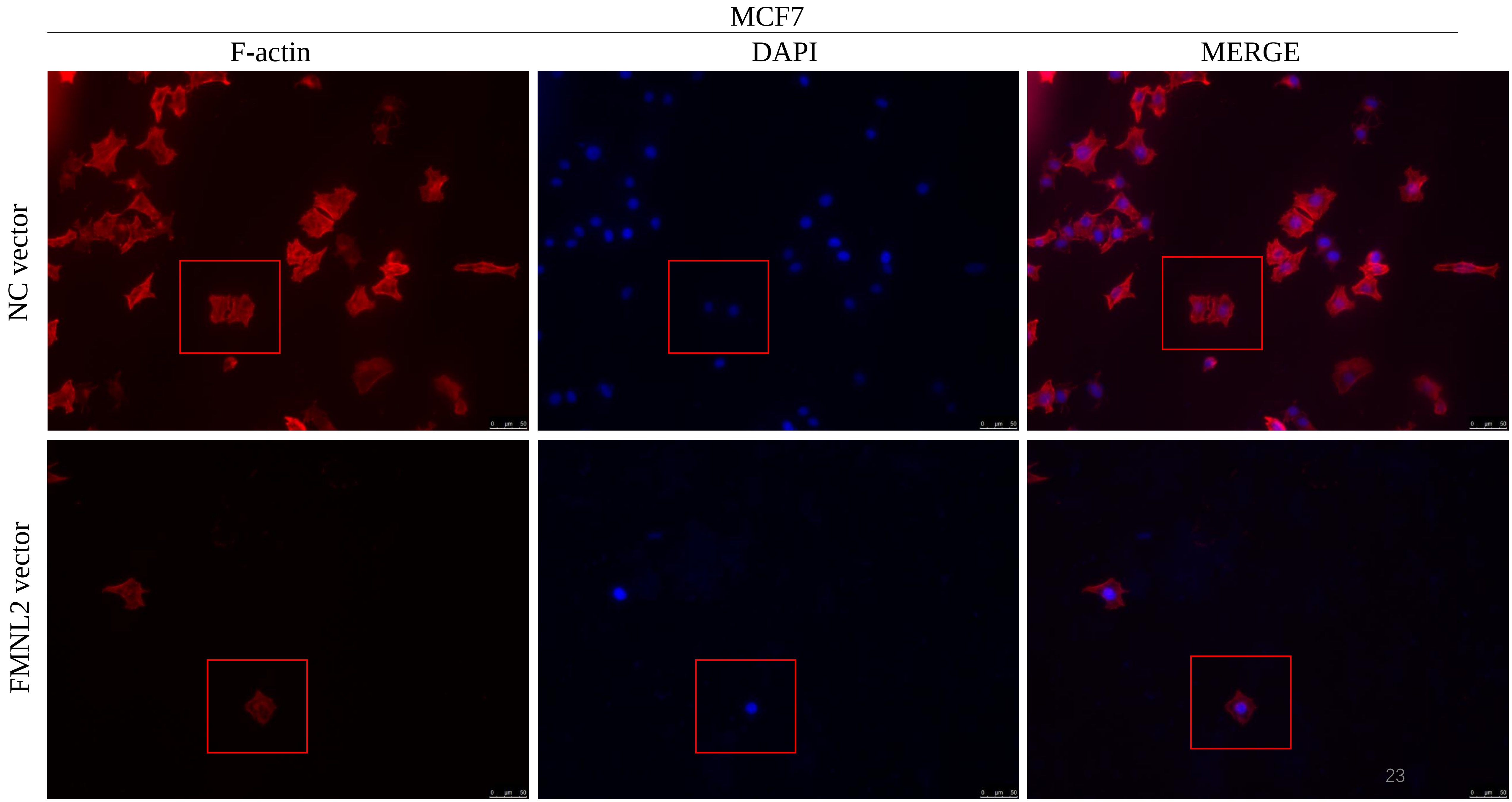

Fig. 2B

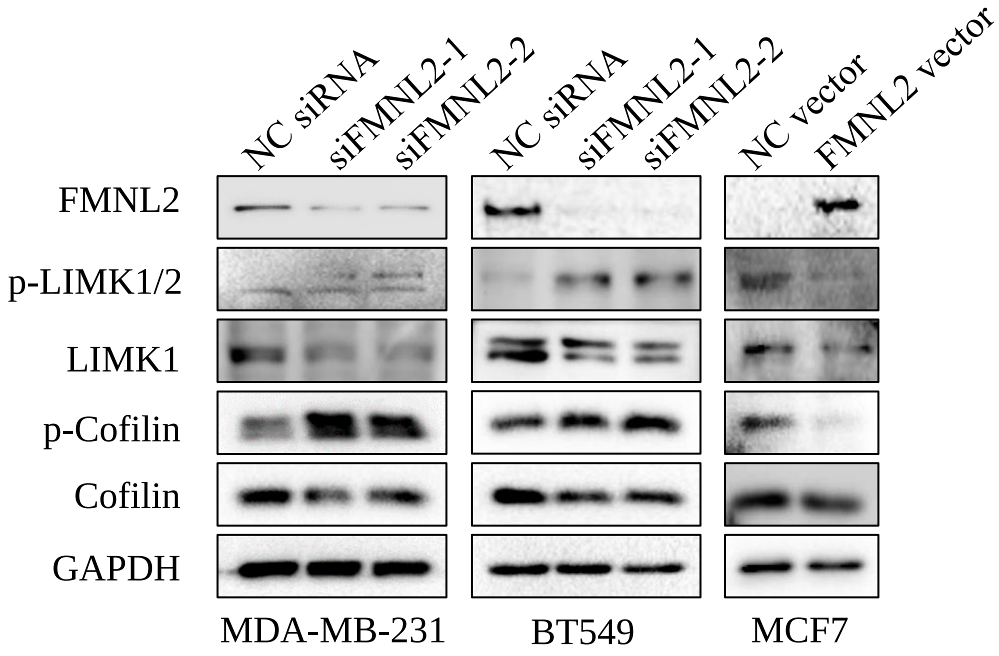
NC siRNA
siFMNL2-1
siFMNL2-2
NC siRNA
siFMNL2-1
siFMNL2-2
NC vector
FMNL2 vector
FMNL2
p-LIMK1/2
LIMK1
p-Cofilin
Cofilin
GAPDH
MDA-MB-231
BT549
MCF7

Fig. 2B

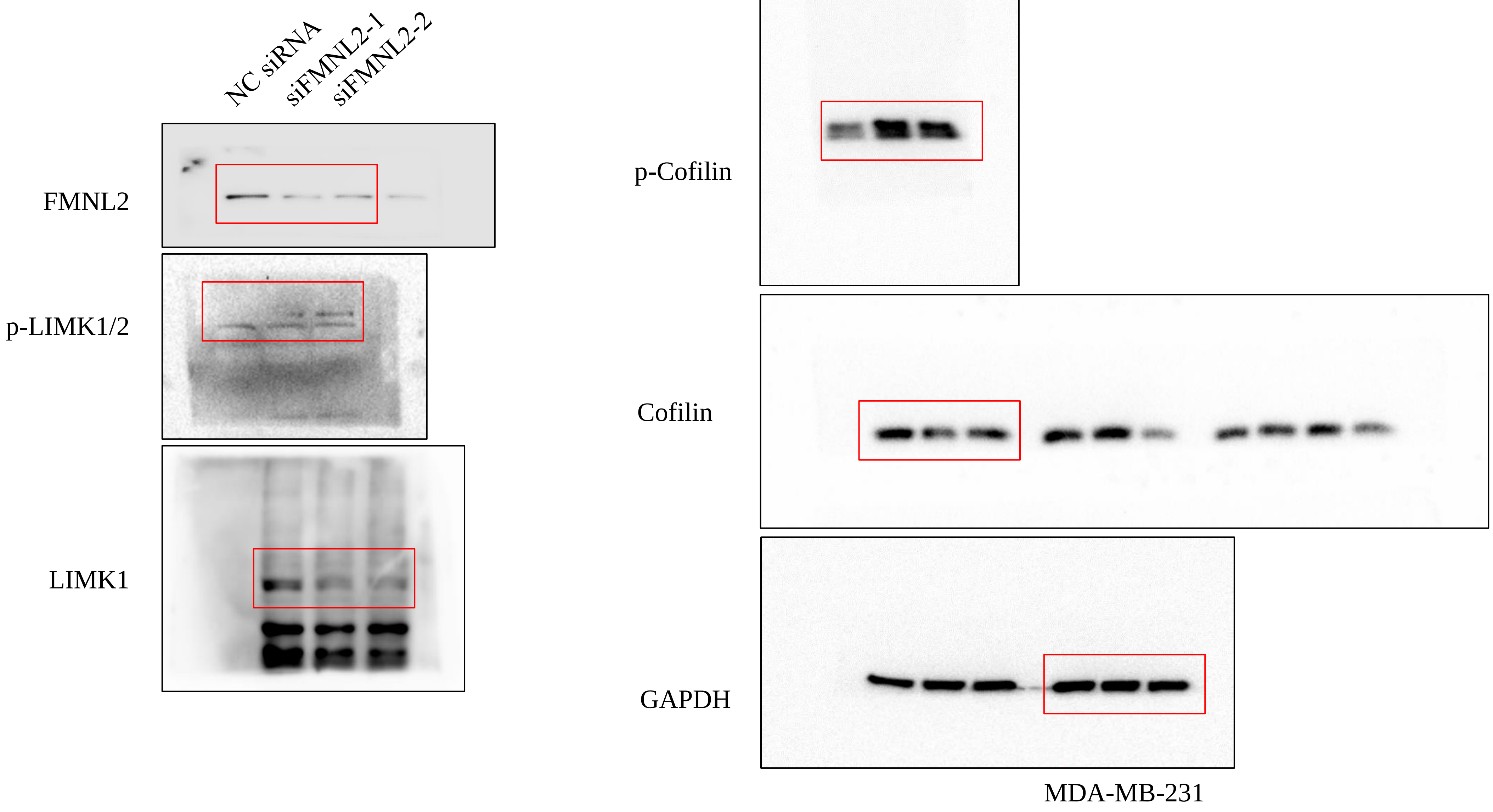
NC siRNA
siFMNL2-1
siFMNL2-2
FMNL2
p-LIMK1/2
LIMK1
p-Cofilin
Cofilin
GAPDH
MDA-MB-231

Fig. 2B

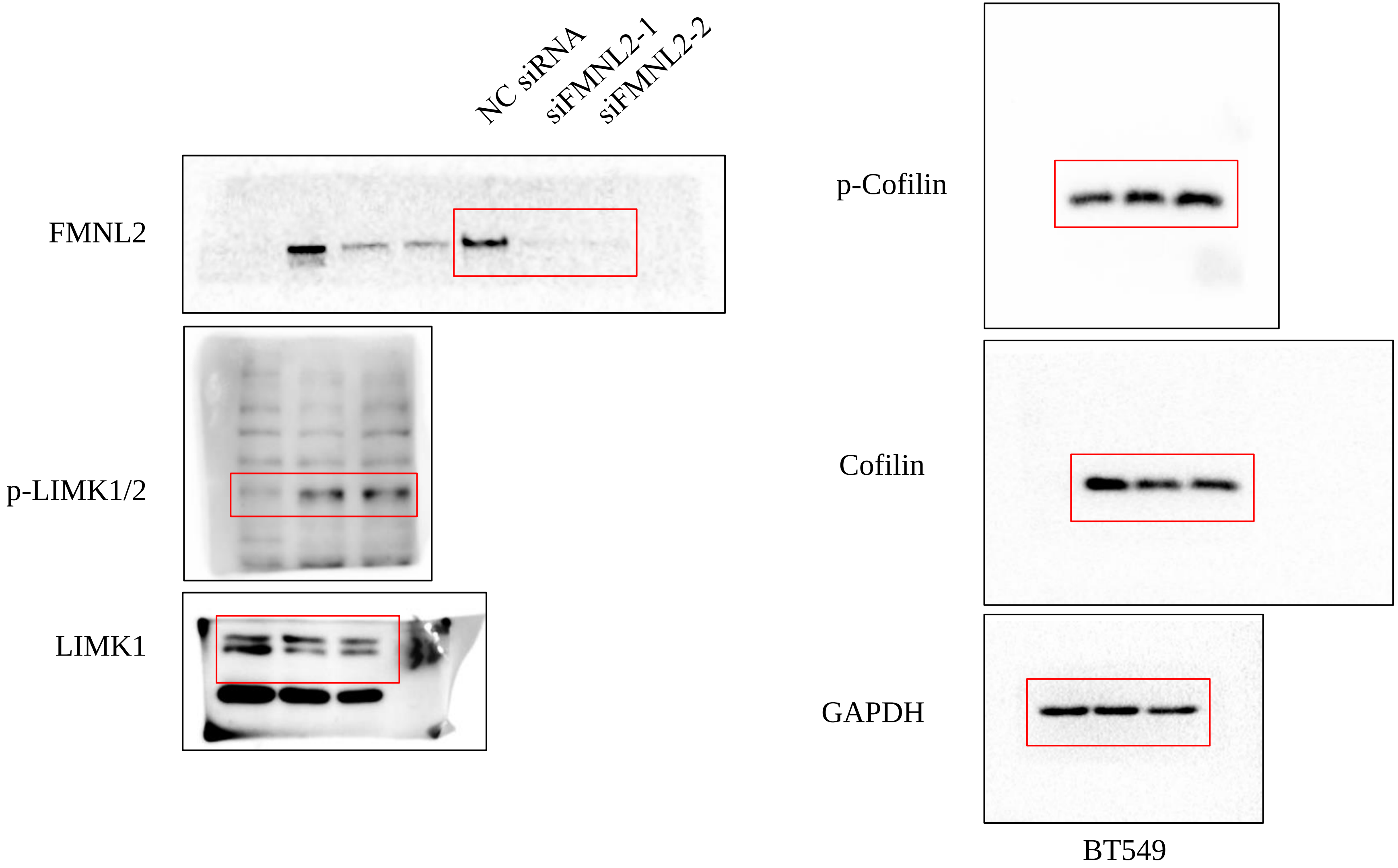
NC siRNA
siFMNL2-1
siFMNL2-2
FMNL2
p-LIMK1/2
LIMK1
p-Cofilin
Cofilin
GAPDH
BT549

Fig. 2B

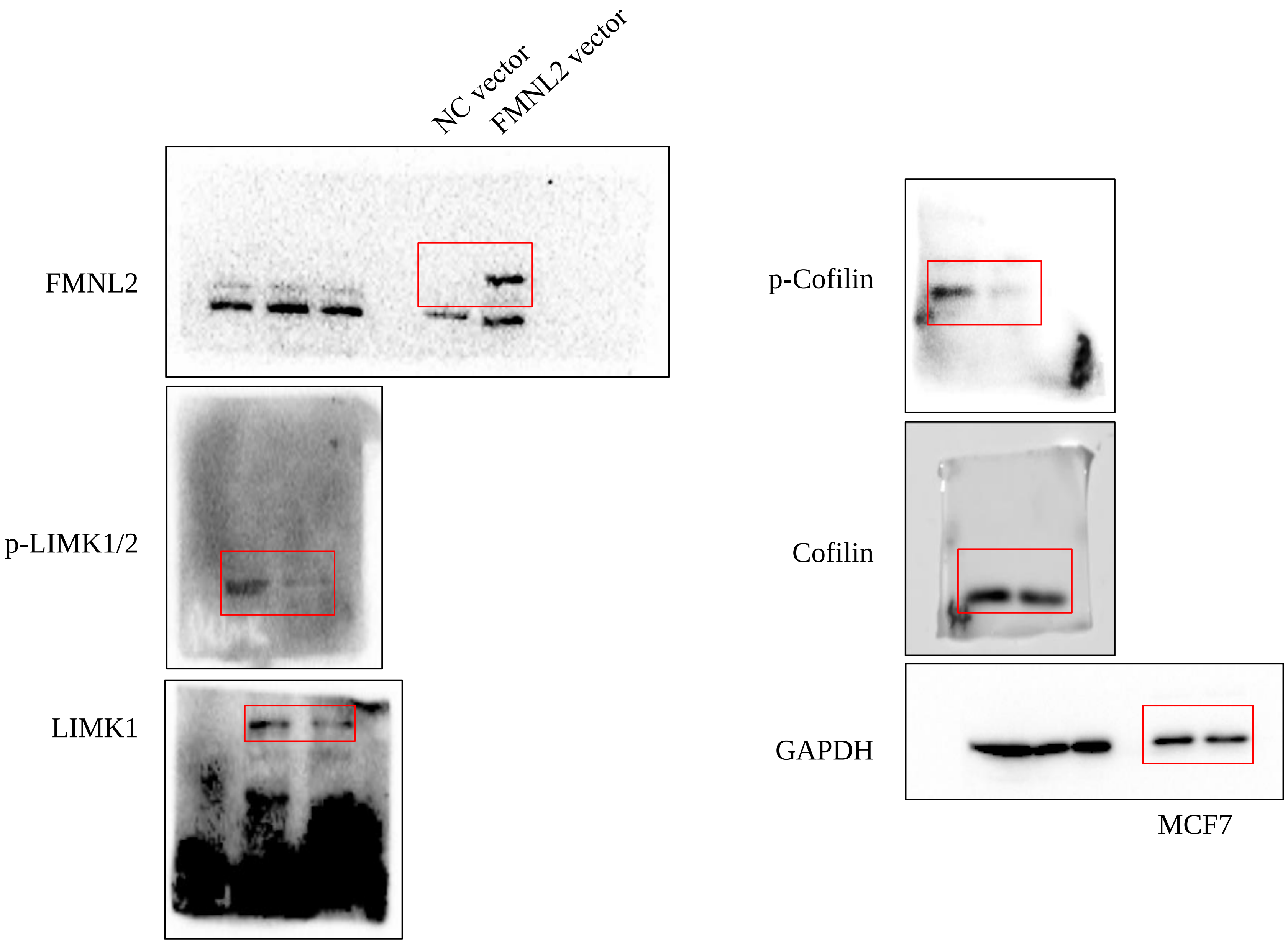
NC vector
FMNL2 vector
FMNL2
p-LIMK1/2
LIMK1
p-Cofilin
Cofilin
GAPDH
MCF7

Fig. 2C

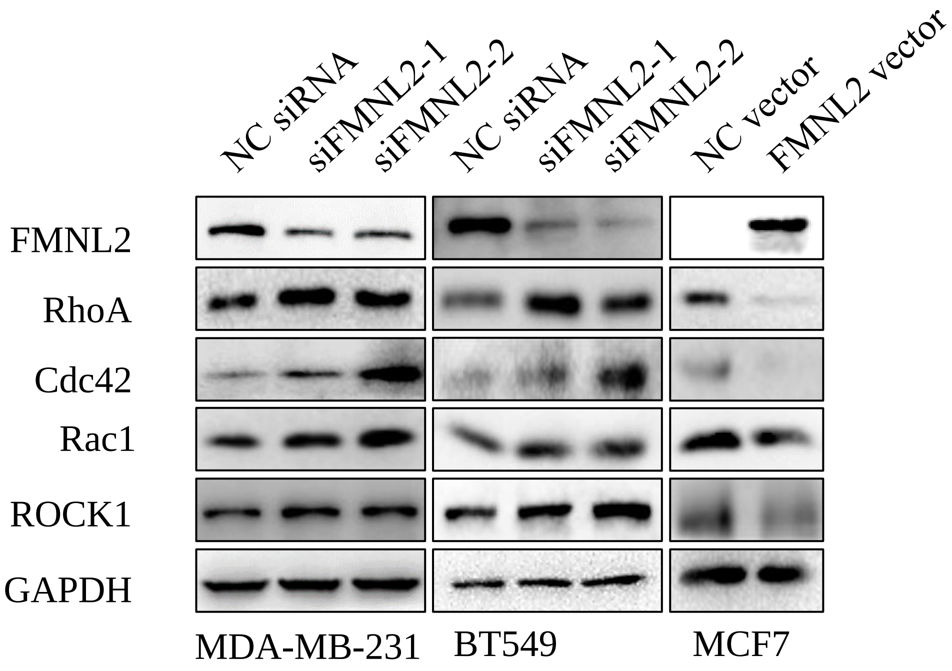
NC siRNA
siFMNL2-1
siFMNL2-2
NC siRNA
siFMNL2-1
siFMNL2-2
NC vector
FMNL2 vector
FMNL2
RhoA
Cdc42
Rac1
ROCK1
GAPDH
MDA-MB-231
BT549
MCF7

Fig. 2C

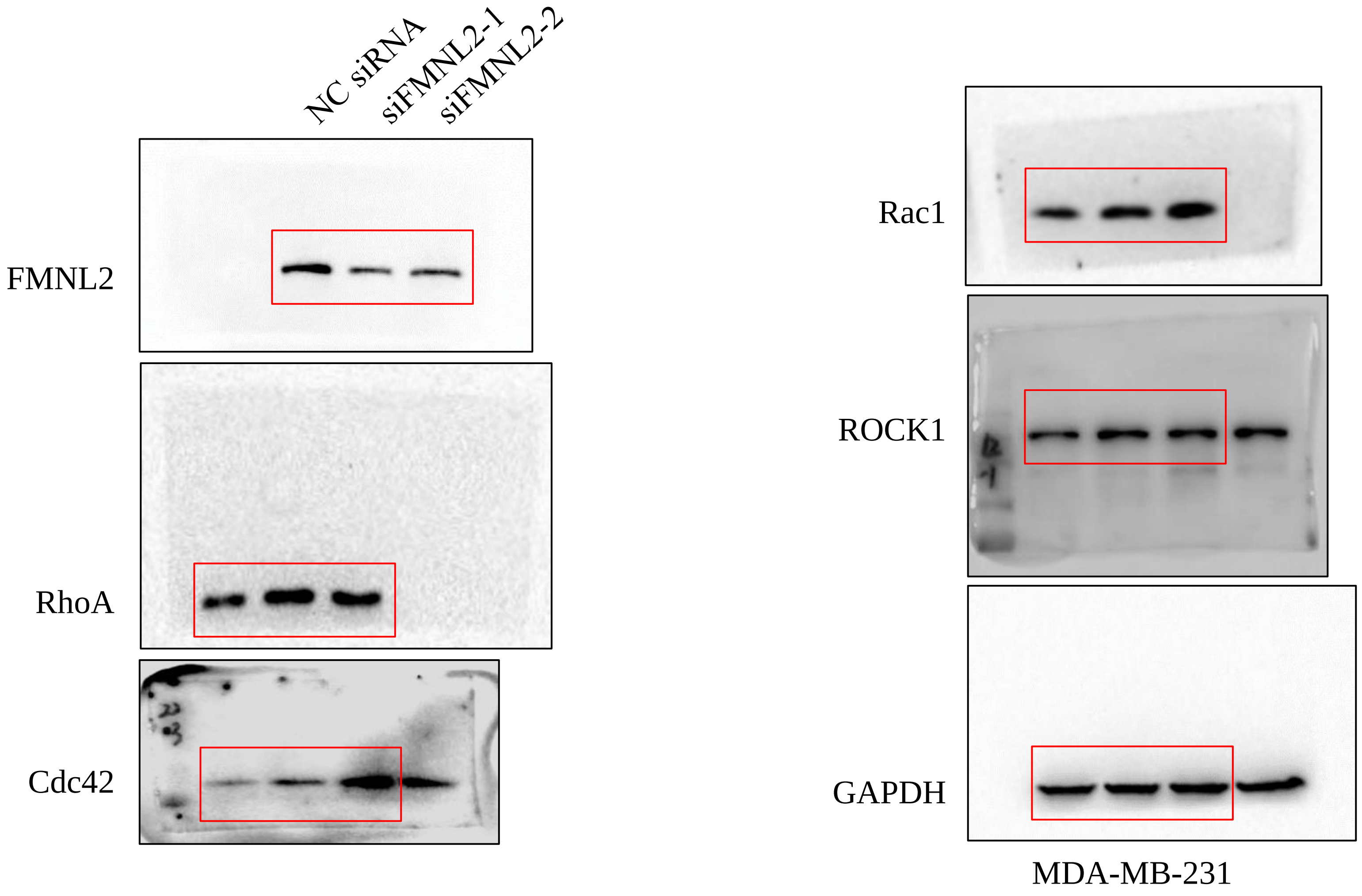
NC siRNA
siFMNL2-1
siFMNL2-2
FMNL2
RhoA
Cdc42
Rac1
ROCK1
GAPDH
MDA-MB-231

Fig. 2C

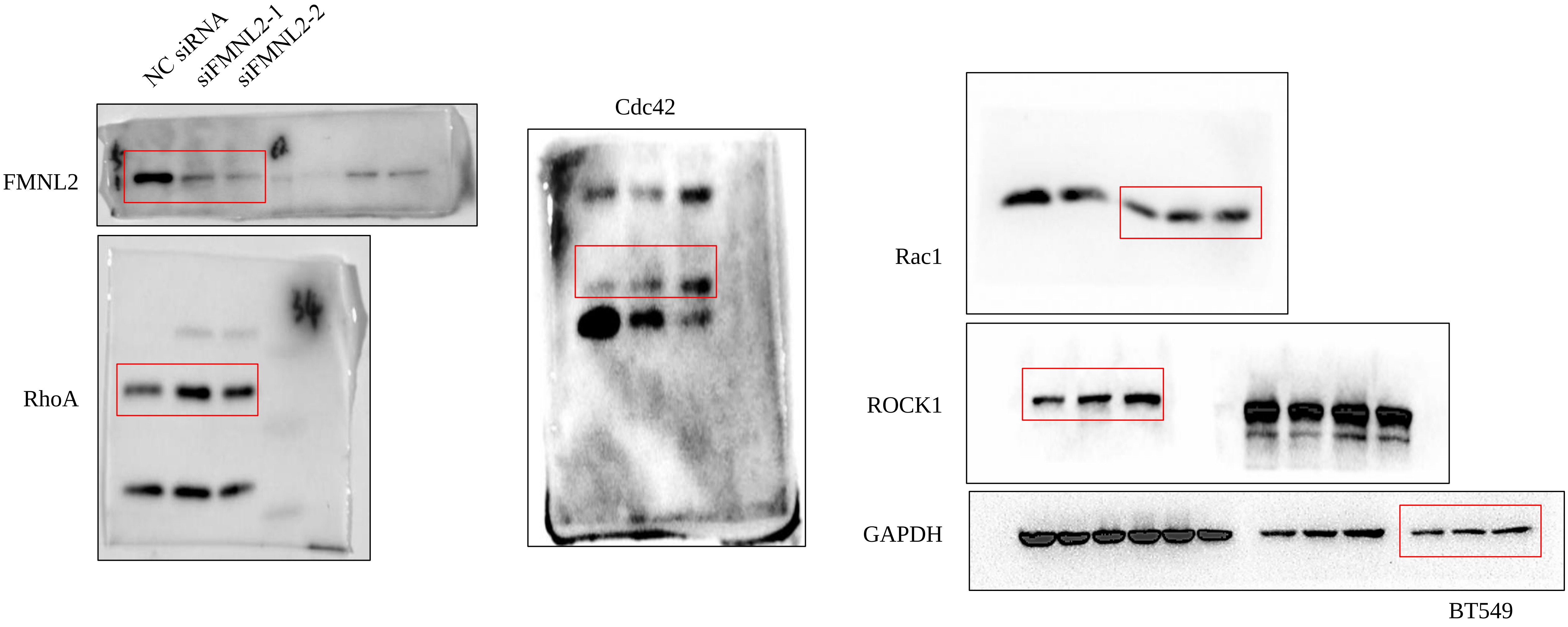
NC siRNA
siFMNL2-1
siFMNL2-2
FMNL2
RhoA
Cdc42
Rac1
ROCK1
GAPDH
BT549

Fig. 2C

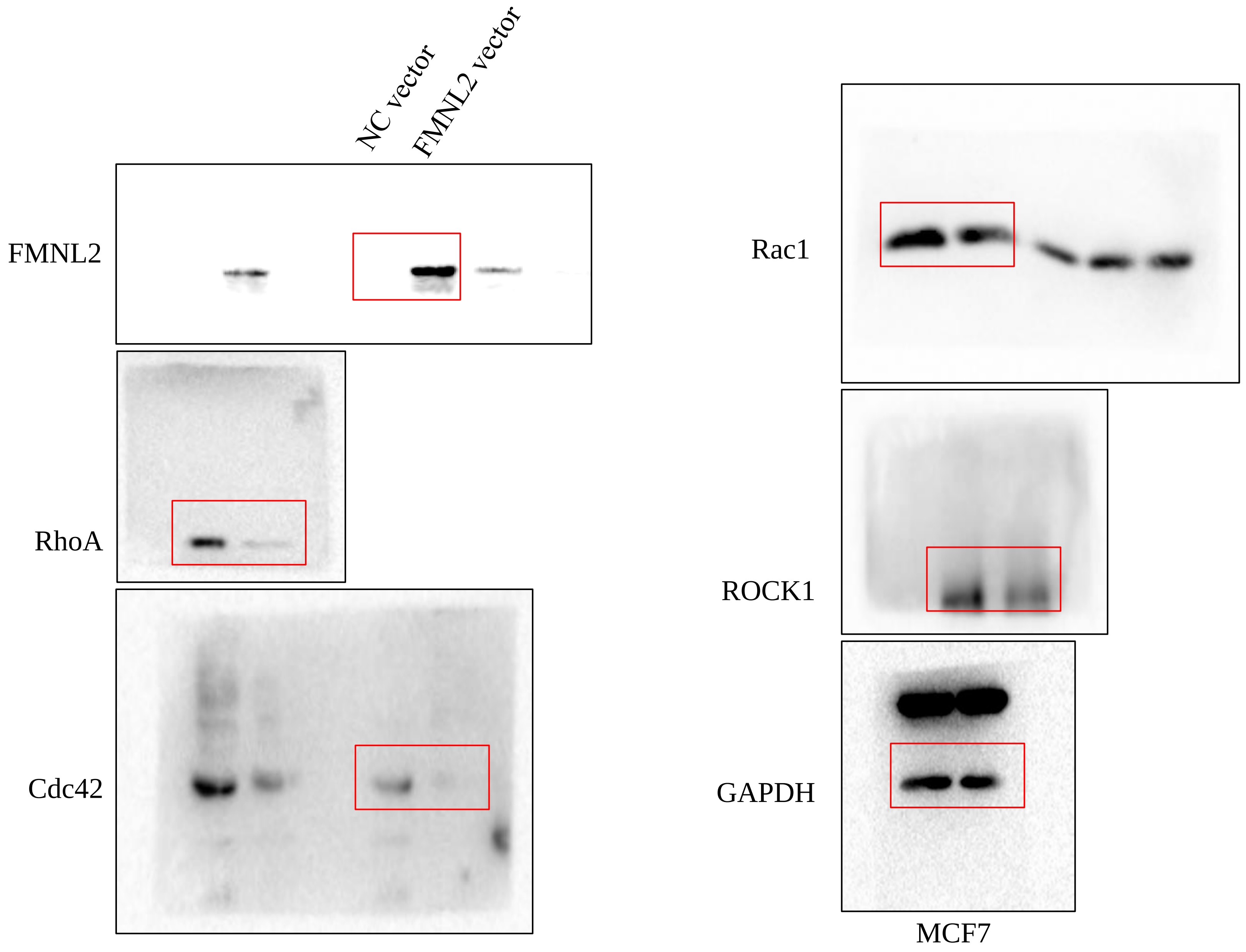
NC vector
FMNL2 vector
FMNL2
RhoA
Cdc42
Rac1
ROCK1
GAPDH
MCF7

Fig. 3A

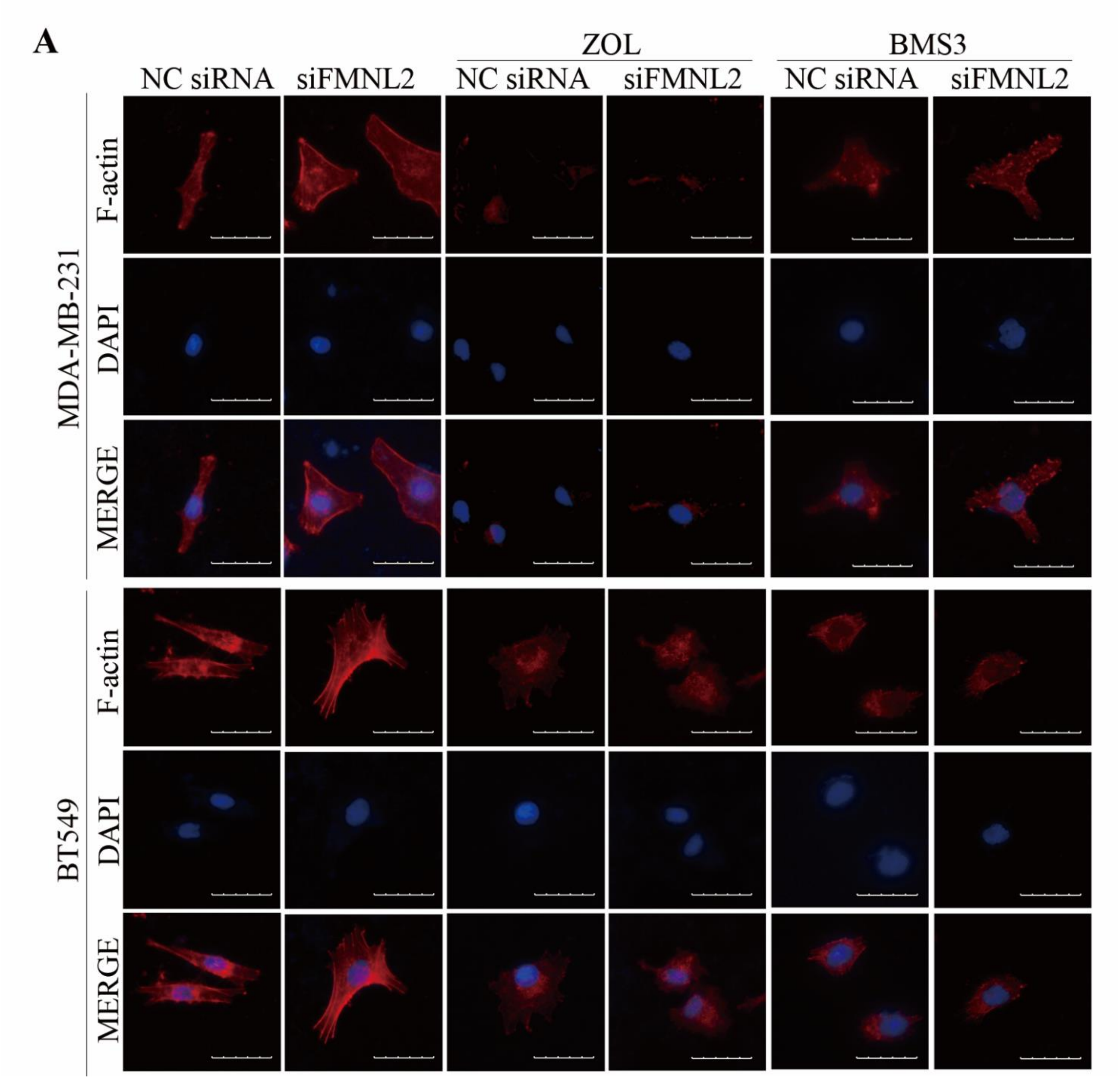
A
ZOL
BMS3
NC siRNA
siFMNL2
NC siRNA
siFMNL2
NC siRNA
siFMNL2
MDA-MB-231
F-actin
DAPI
MERGE
BT549
F-actin
DAPI
MERGE

Fig. 3A

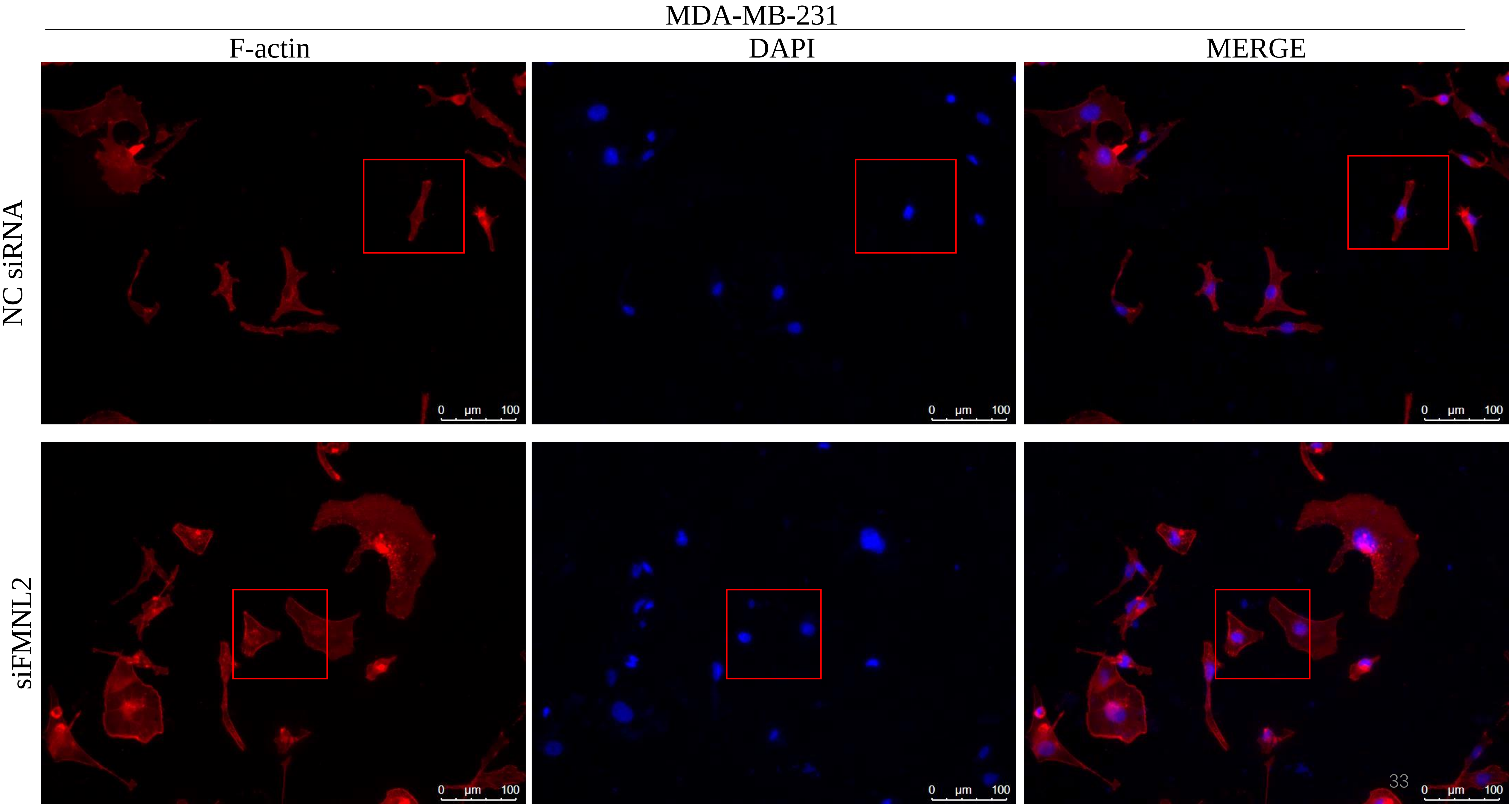

Fig. 3A

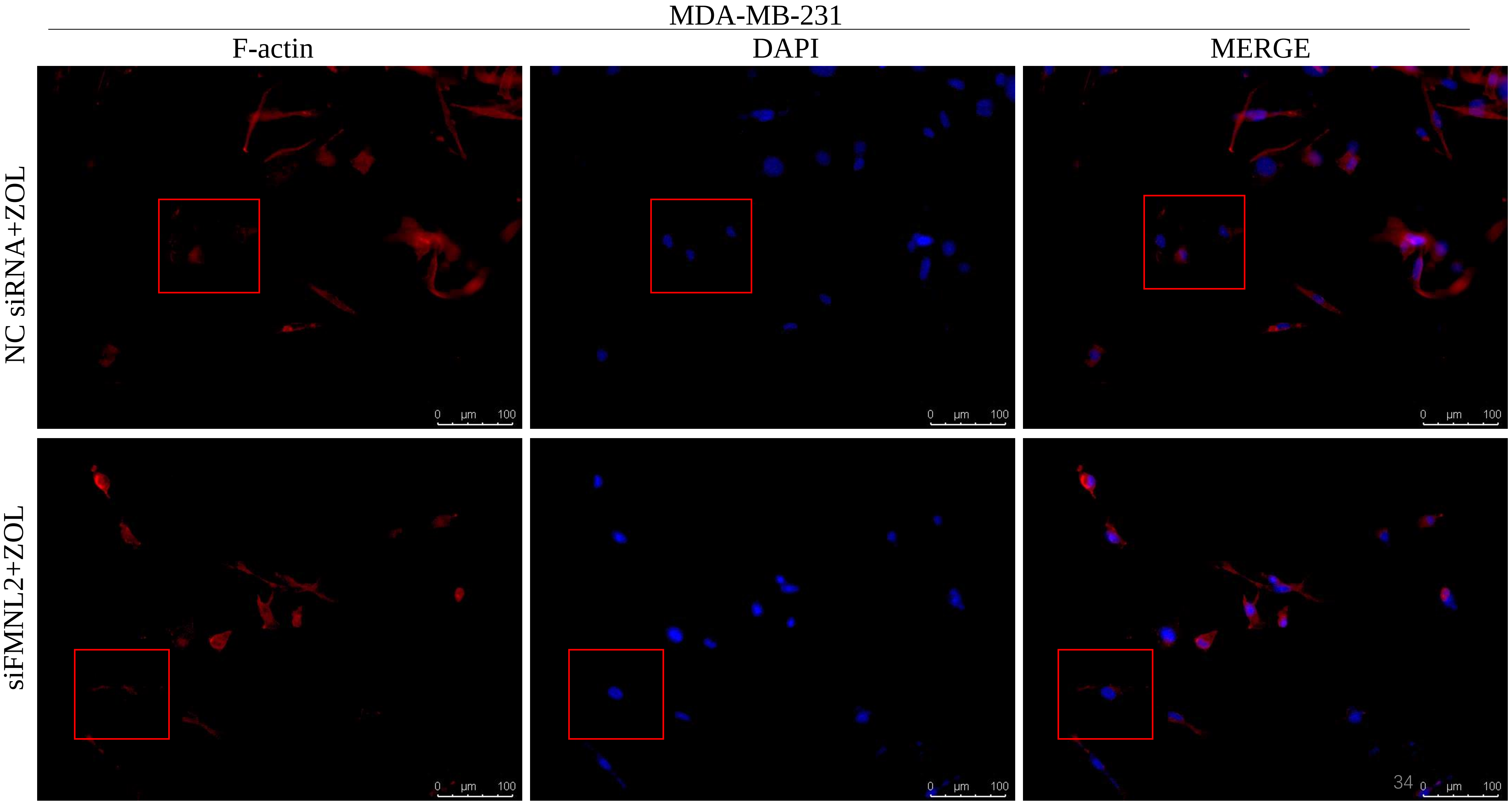

Fig. 3A

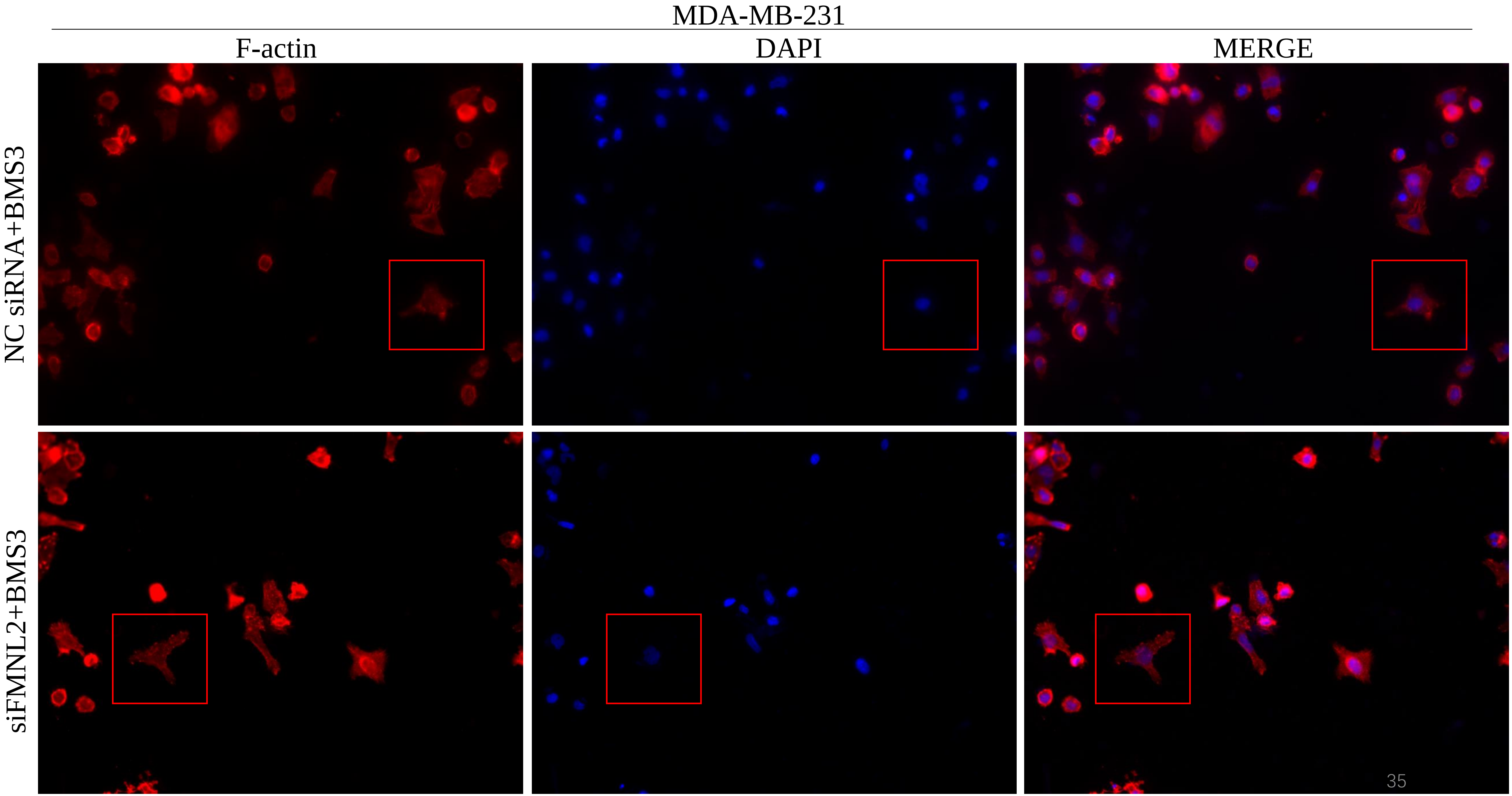

Fig. 3A

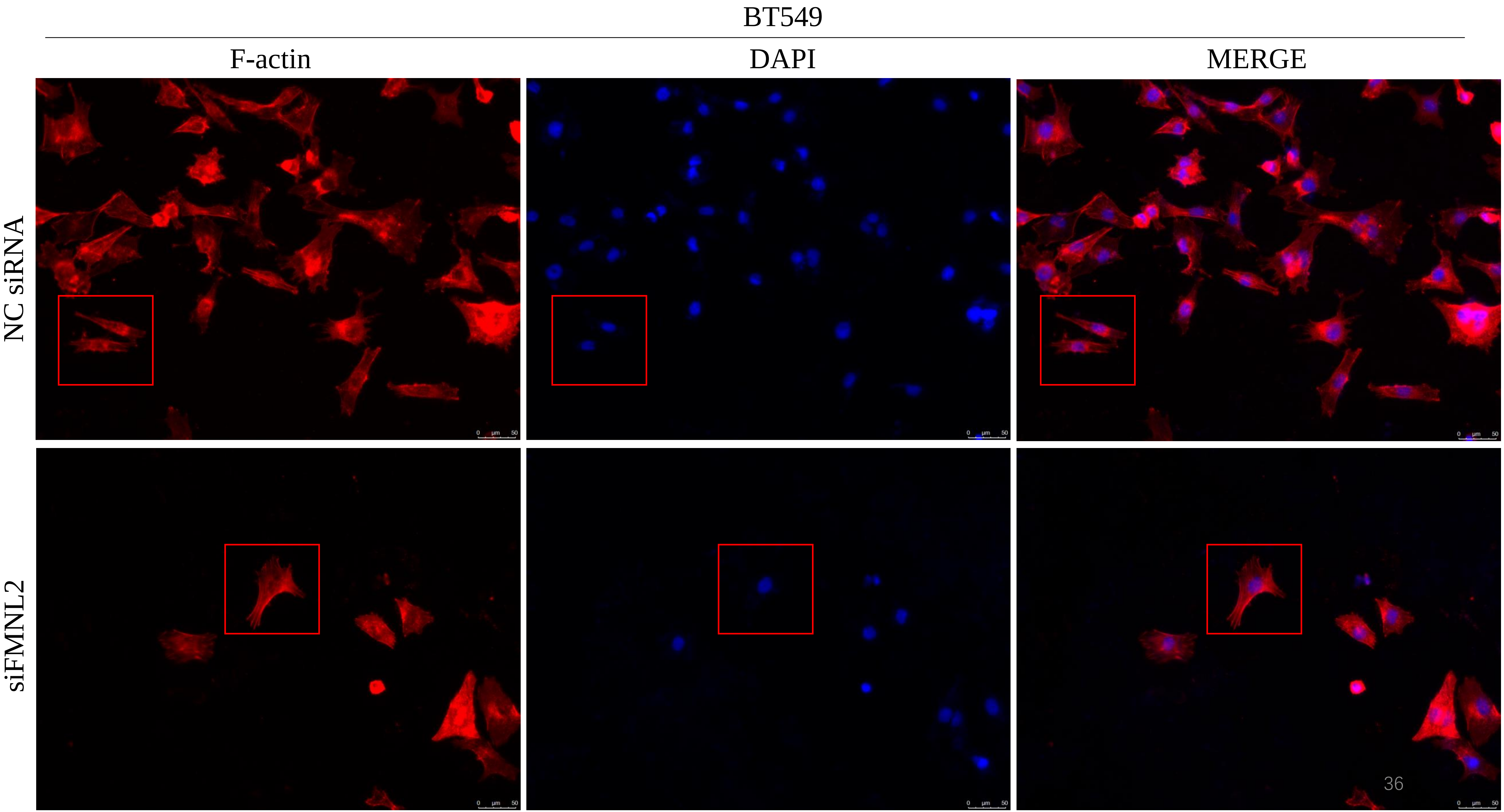

Fig. 3A

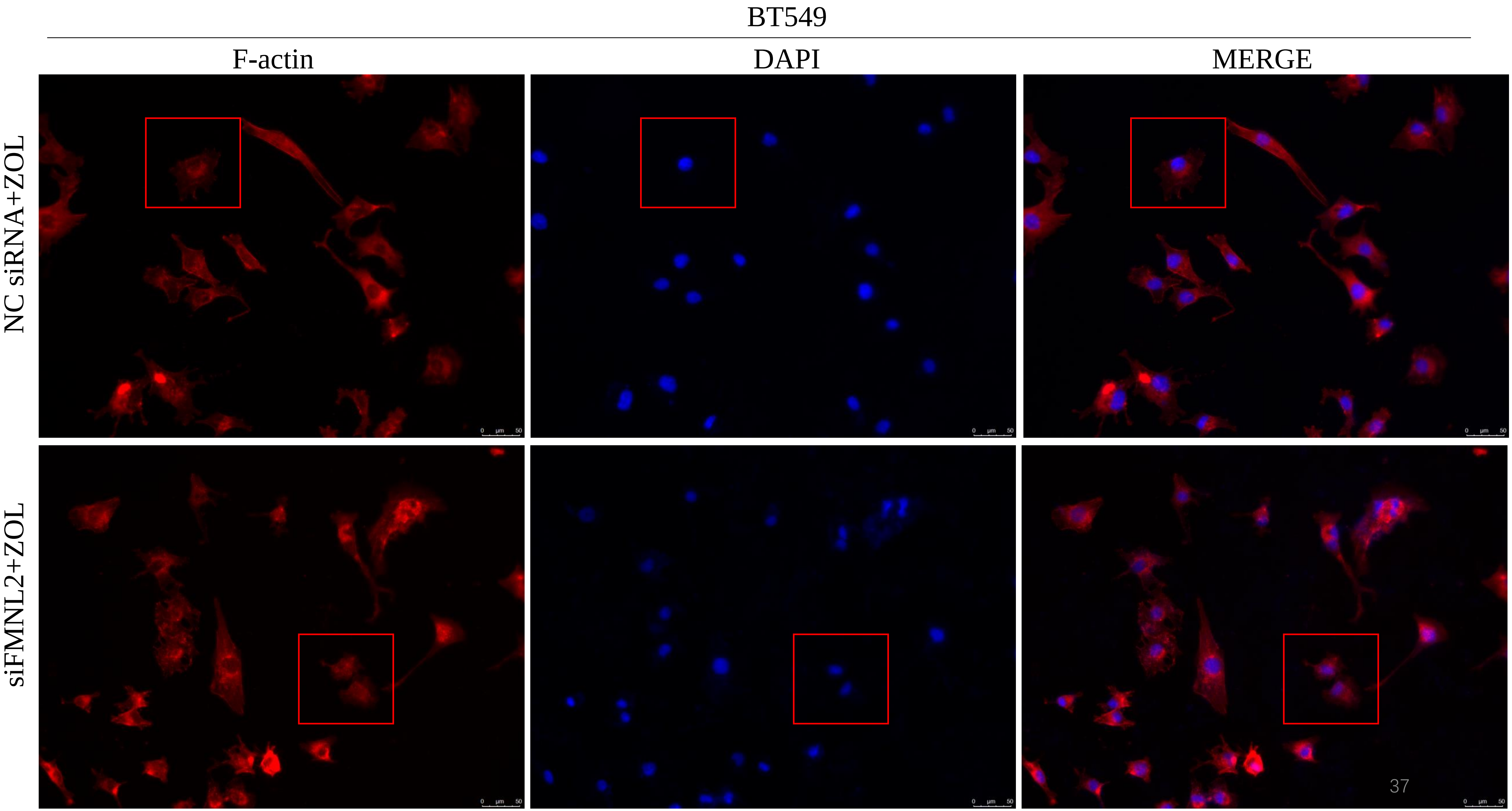

Fig. 3A

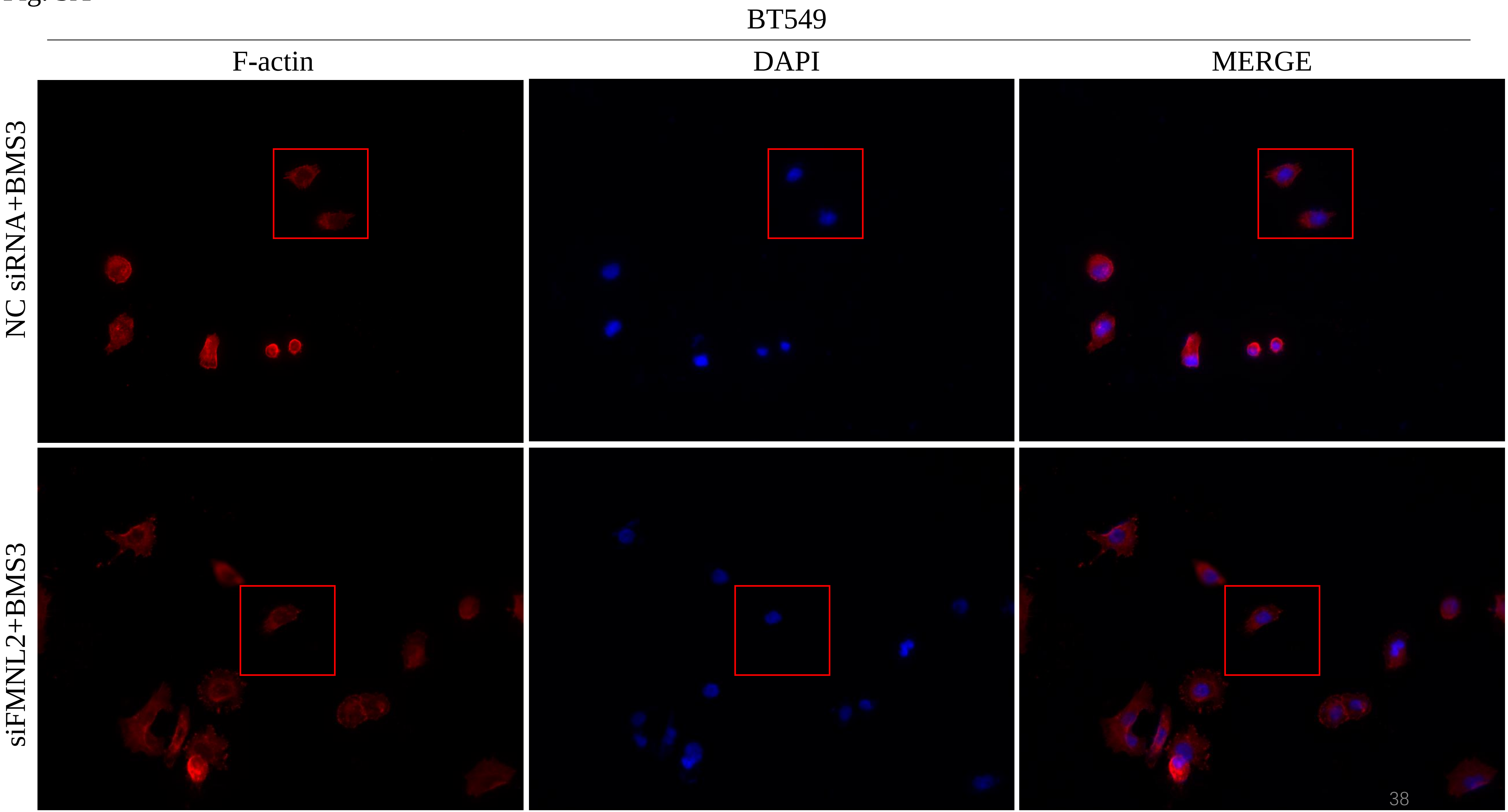

Fig. 3B

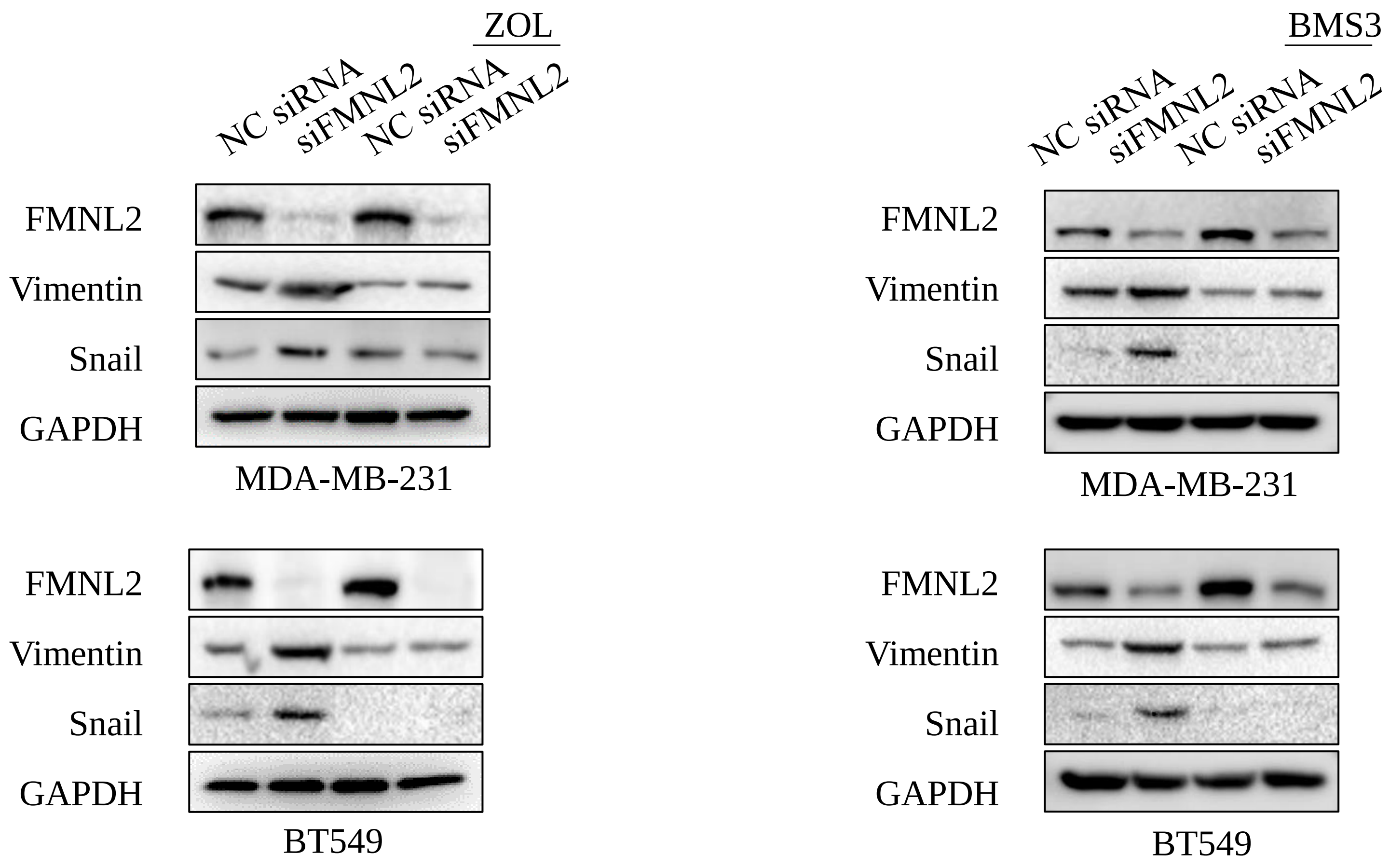
ZOL
NC siRNA
siFMNL2
NC siRNA
siFMNL2
FMNL2
Vimentin
Snail
GAPDH
MDA-MB-231
FMNL2
Vimentin
Snail
GAPDH
BT549
BMS3
NC siRNA
siFMNL2
NC siRNA
siFMNL2
FMNL2
Vimentin
Snail
GAPDH
MDA-MB-231
FMNL2
Vimentin
Snail
GAPDH
BT549

Fig. 3B

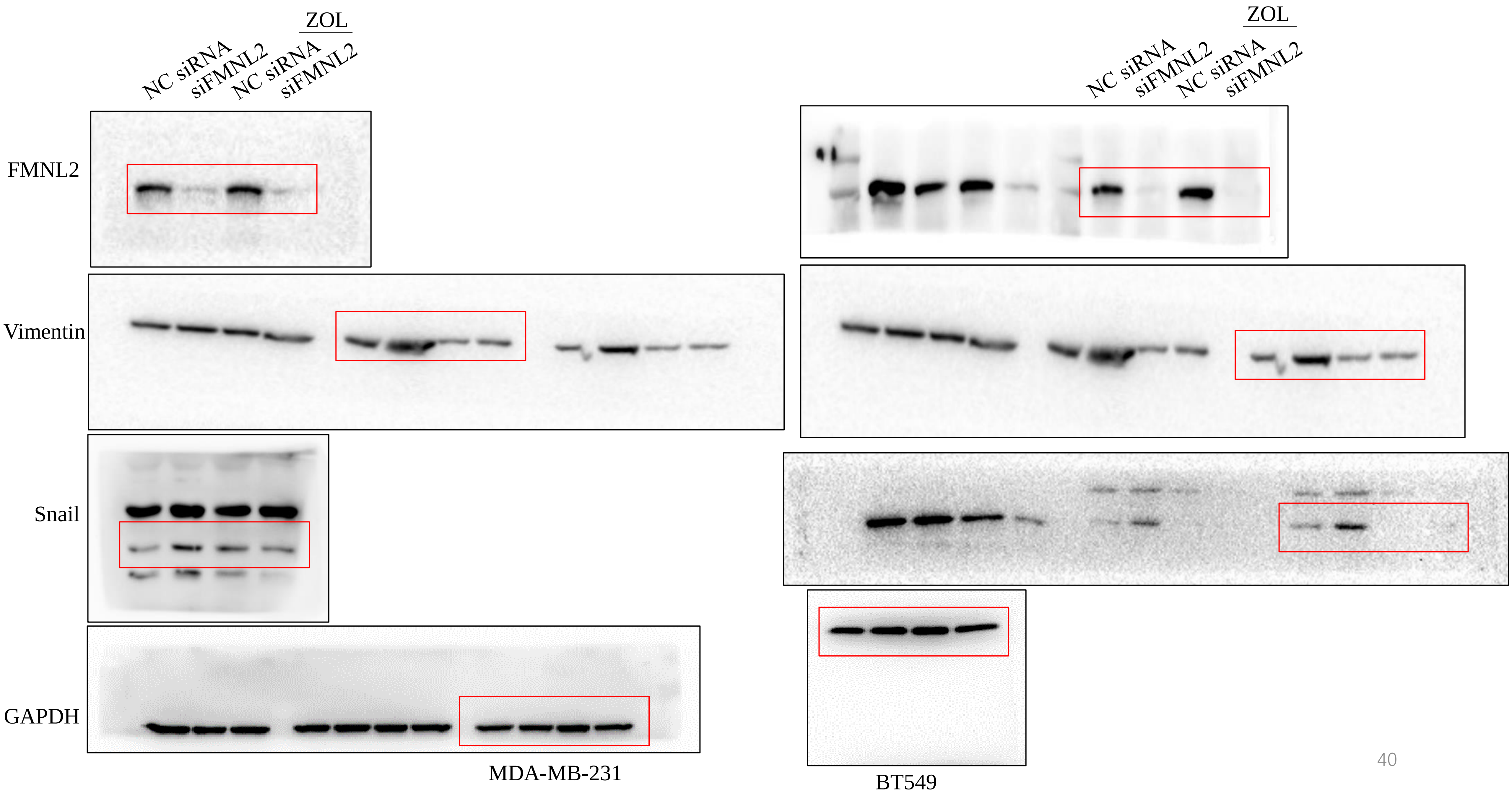
ZOL
NC siRNA
siFMNL2
NC siRNA
siFMNL2
FMNL2
Vimentin
Snail
GAPDH
MDA-MB-231
ZOL
NC siRNA
siFMNL2
NC siRNA
siFMNL2
BT549

Fig. 3B

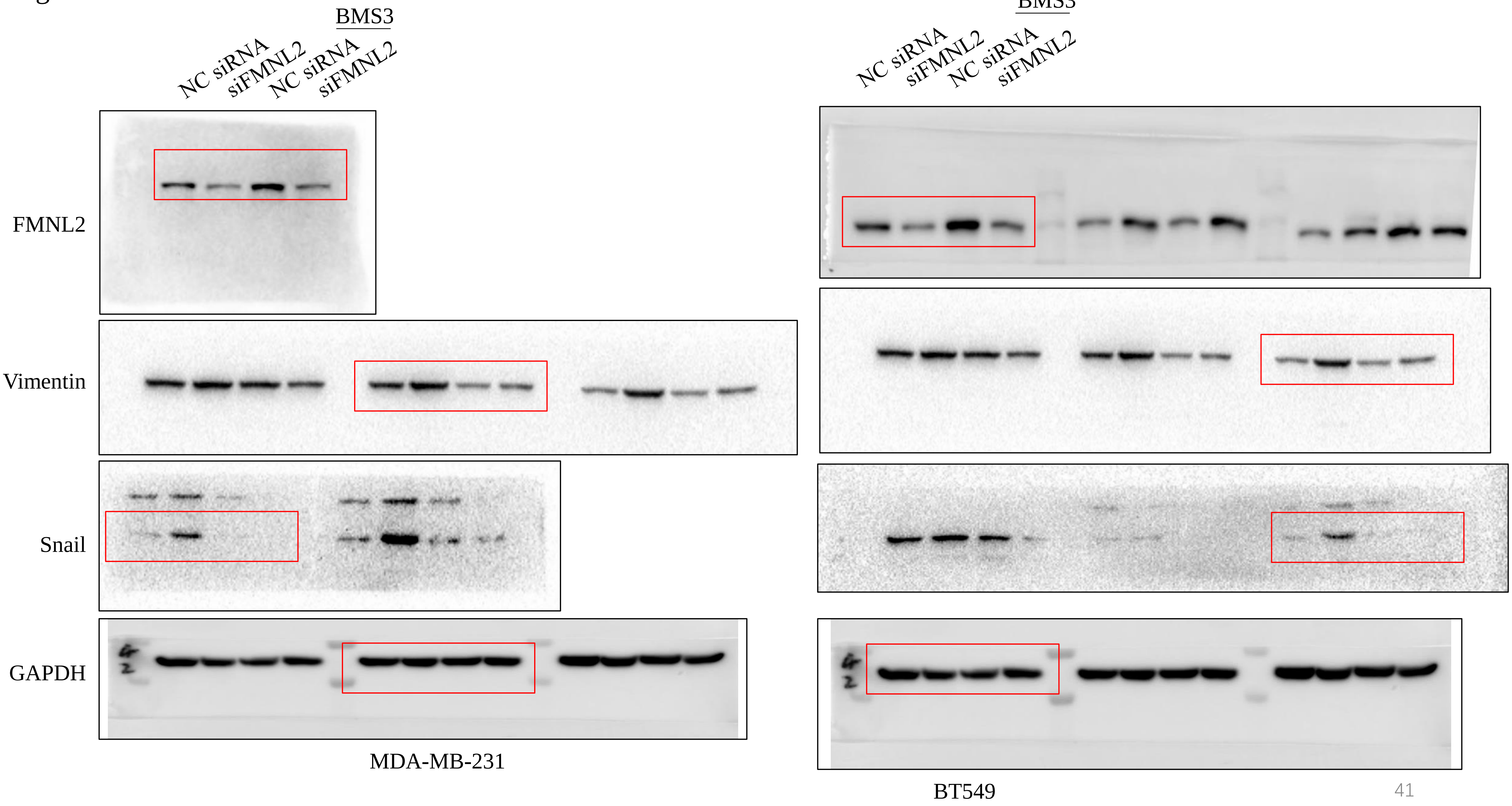
BMS3
NC siRNA
siFMNL2
NC siRNA
siFMNL2
FMNL2
Vimentin
Snail
GAPDH
MDA-MB-231
BMS3
NC siRNA
siFMNL2
NC siRNA
siFMNL2
BT549

Fig. 3C

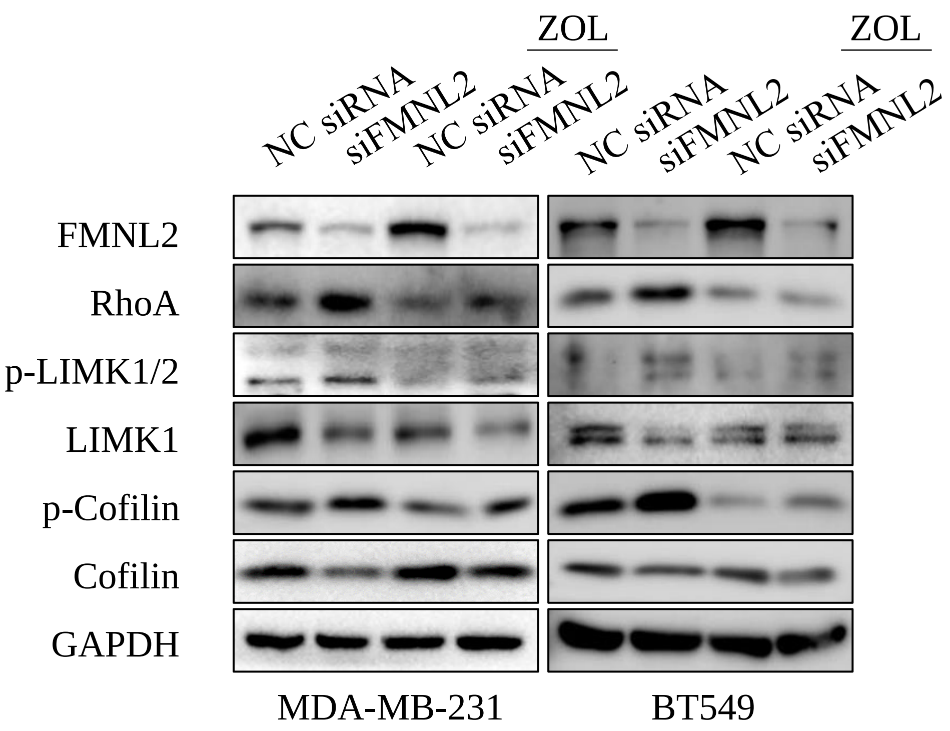
ZOL
ZOL
NC siRNA
siFMNL2
NC siRNA
siFMNL2
NC siRNA
siFMNL2
NC siRNA
siFMNL2
FMNL2
RhoA
p-LIMK1/2
LIMK1
p-Cofilin
Cofilin
GAPDH
MDA-MB-231
BT549

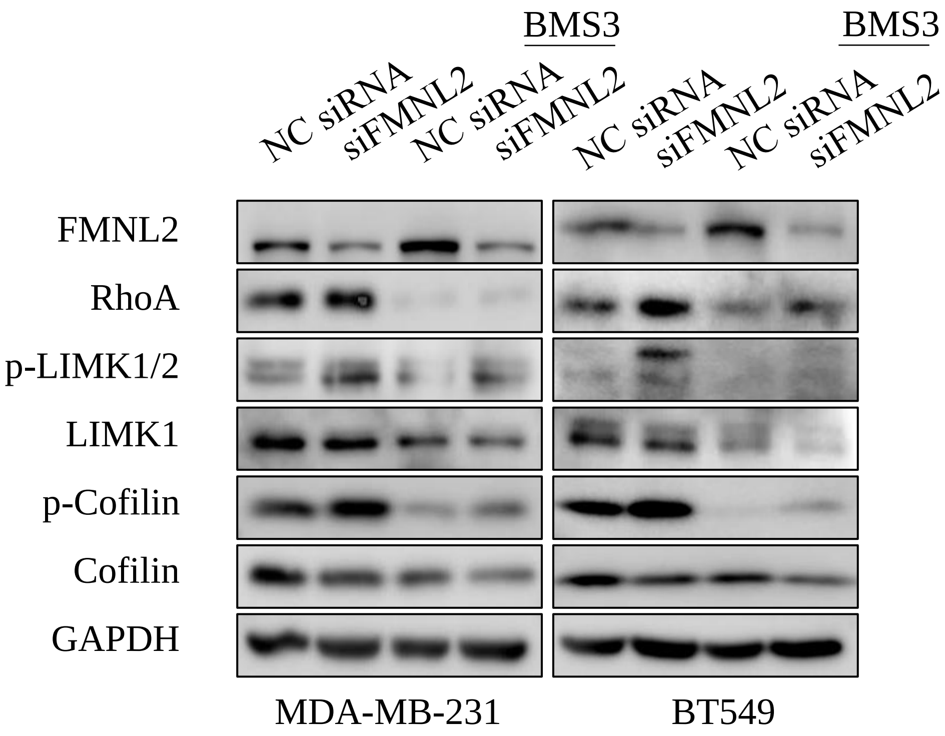
BMS3
BMS3
NC siRNA
siFMNL2
NC siRNA
siFMNL2
NC siRNA
siFMNL2
NC siRNA
siFMNL2
FMNL2
RhoA
p-LIMK1/2
LIMK1
p-Cofilin
Cofilin
GAPDH
MDA-MB-231
BT549

Fig. 3C

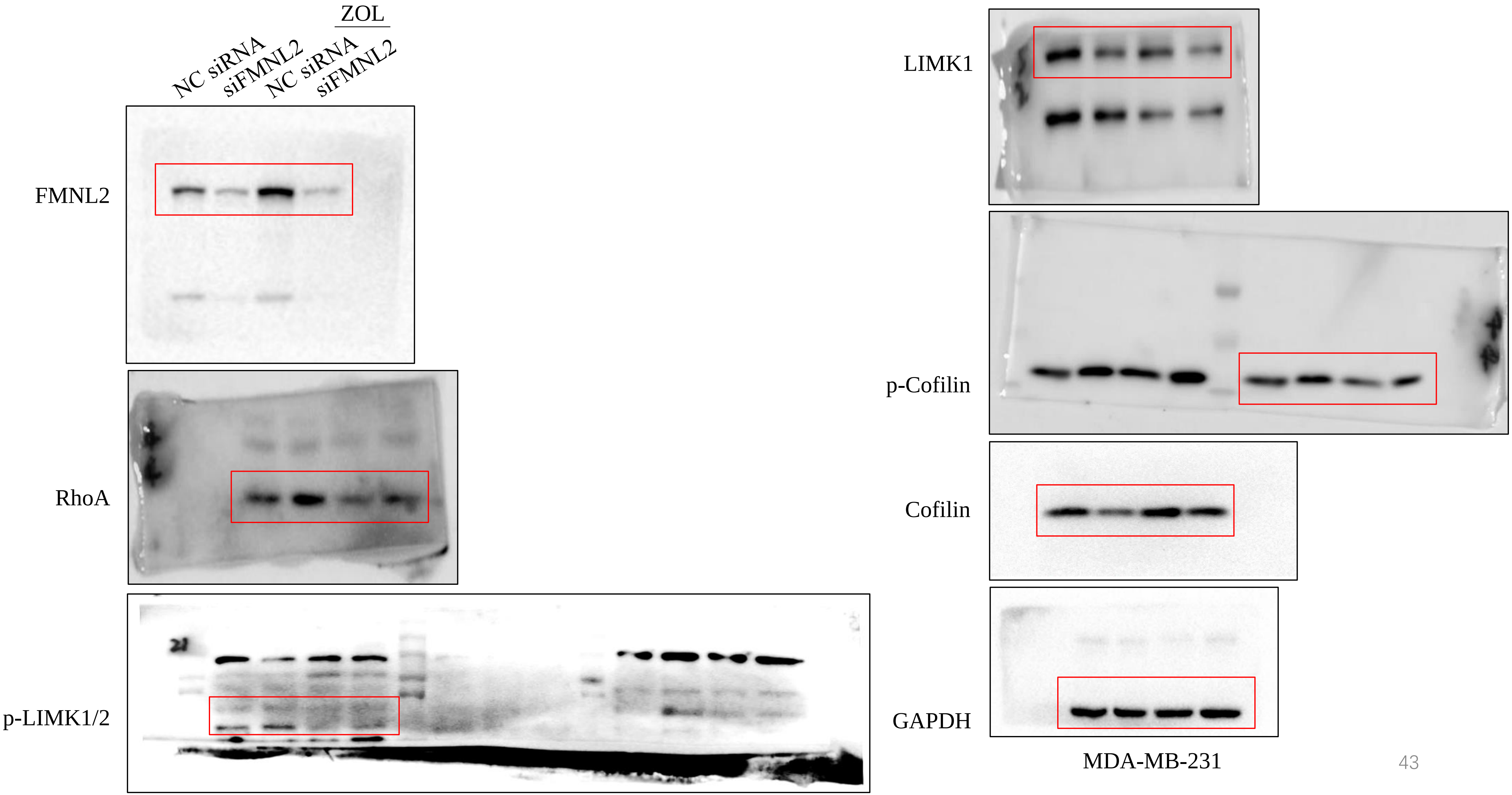
ZOL
NC siRNA
siFMNL2
NC siRNA
siFMNL2
FMNL2
RhoA
p-LIMK1/2
LIMK1
p-Cofilin
Cofilin
GAPDH
MDA-MB-231

Fig. 3C

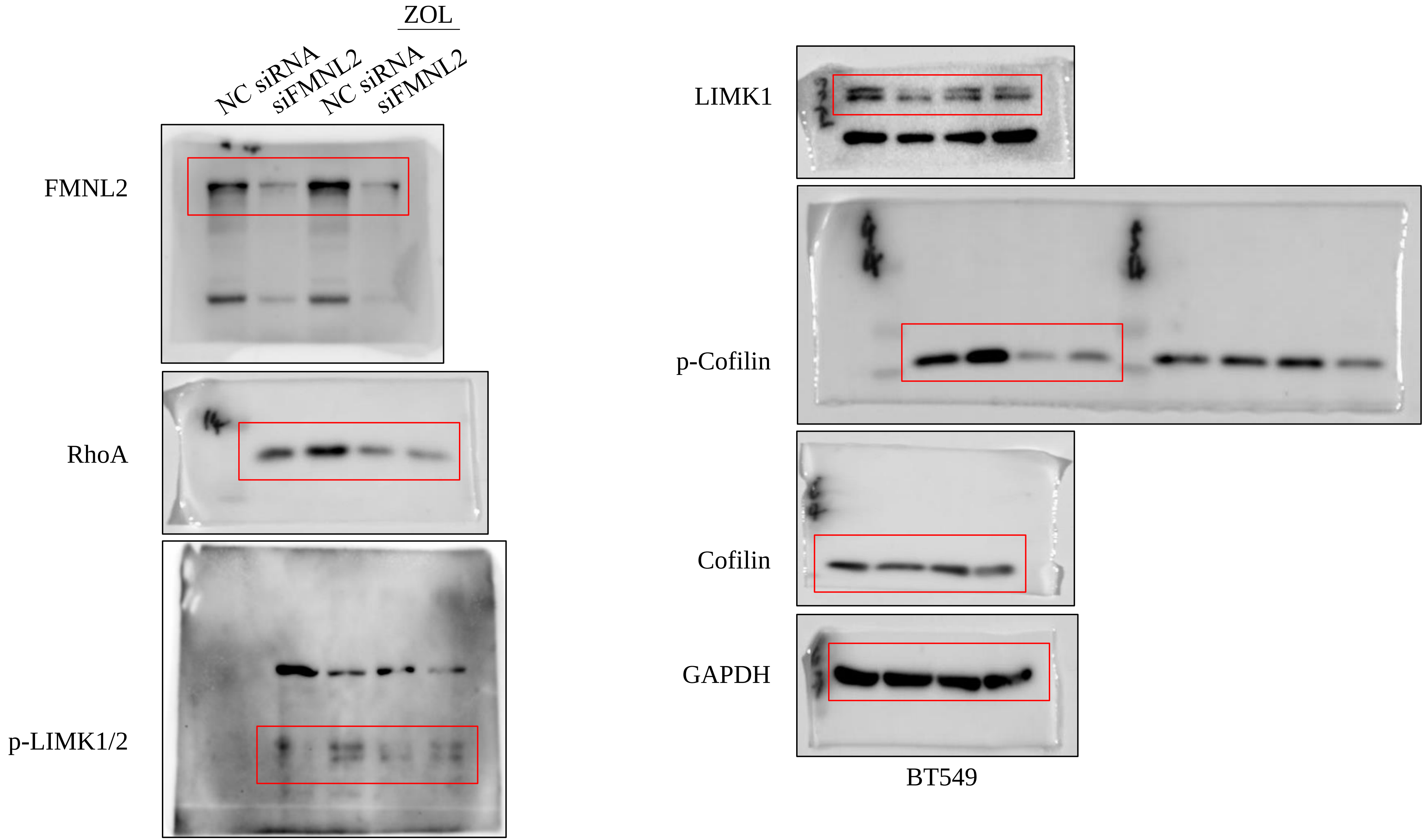
ZOL
NC siRNA
siFMNL2
NC siRNA
siFMNL2
FMNL2
RhoA
p-LIMK1/2
LIMK1
p-Cofilin
Cofilin
GAPDH
BT549

Fig. 3C

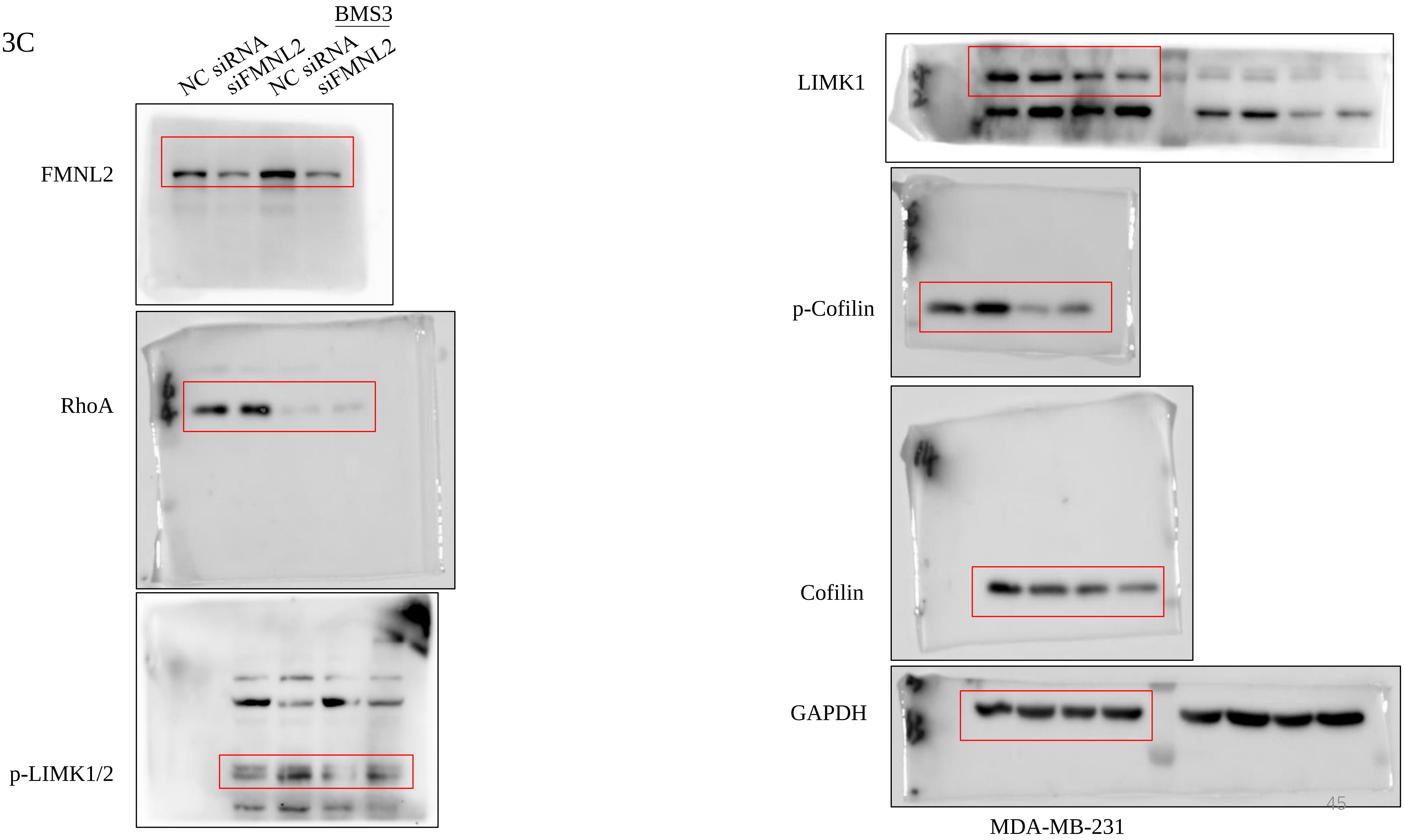
BMS3
NC siRNA
siFMNL2
NC siRNA
siFMNL2
FMNL2
RhoA
p-LIMK1/2
LIMK1
p-Cofilin
Cofilin
GAPDH
MDA-MB-231

Fig. 3C

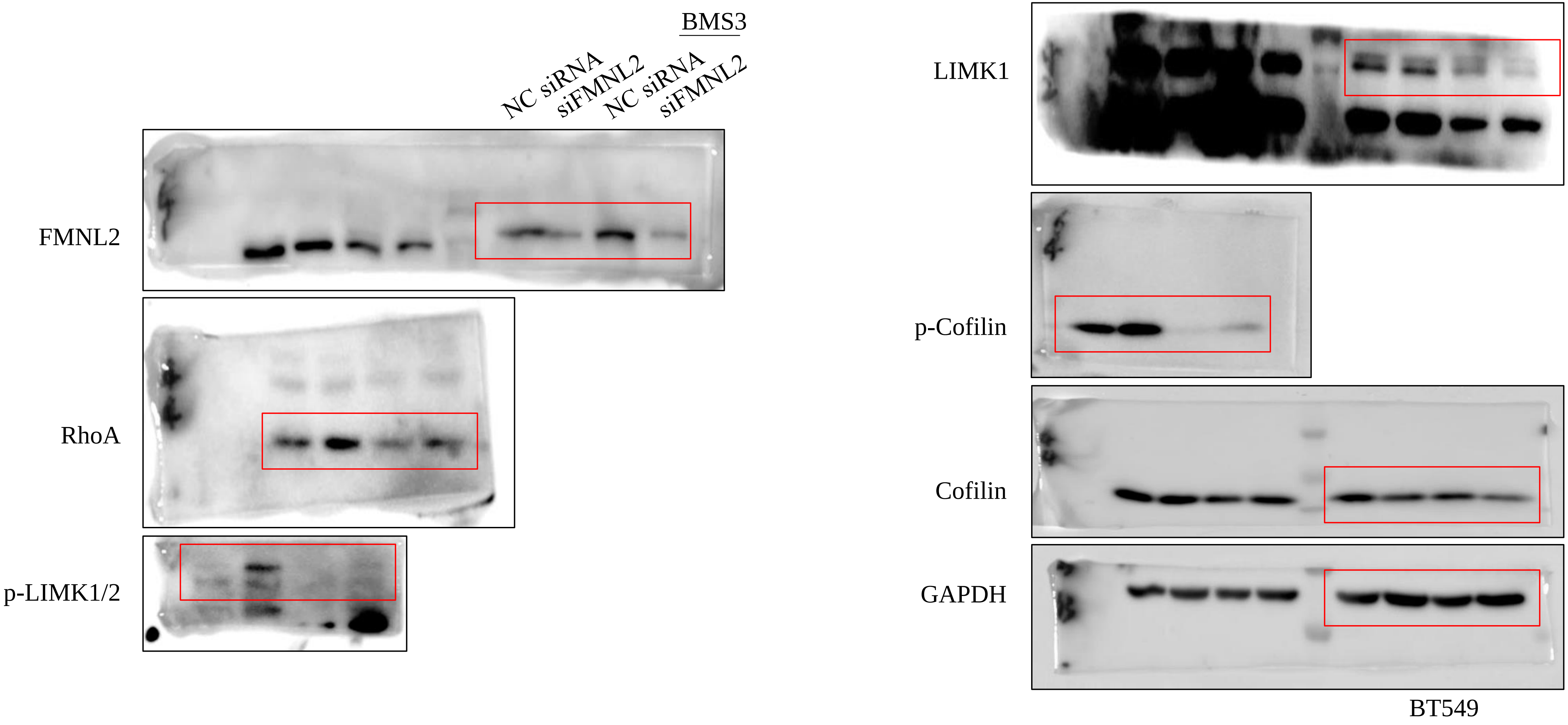
BMS3
NC siRNA
siFMNL2
NC siRNA
siFMNL2
FMNL2
RhoA
p-LIMK1/2
LIMK1
p-Cofilin
Cofilin
GAPDH
BT549

Fig. 3D

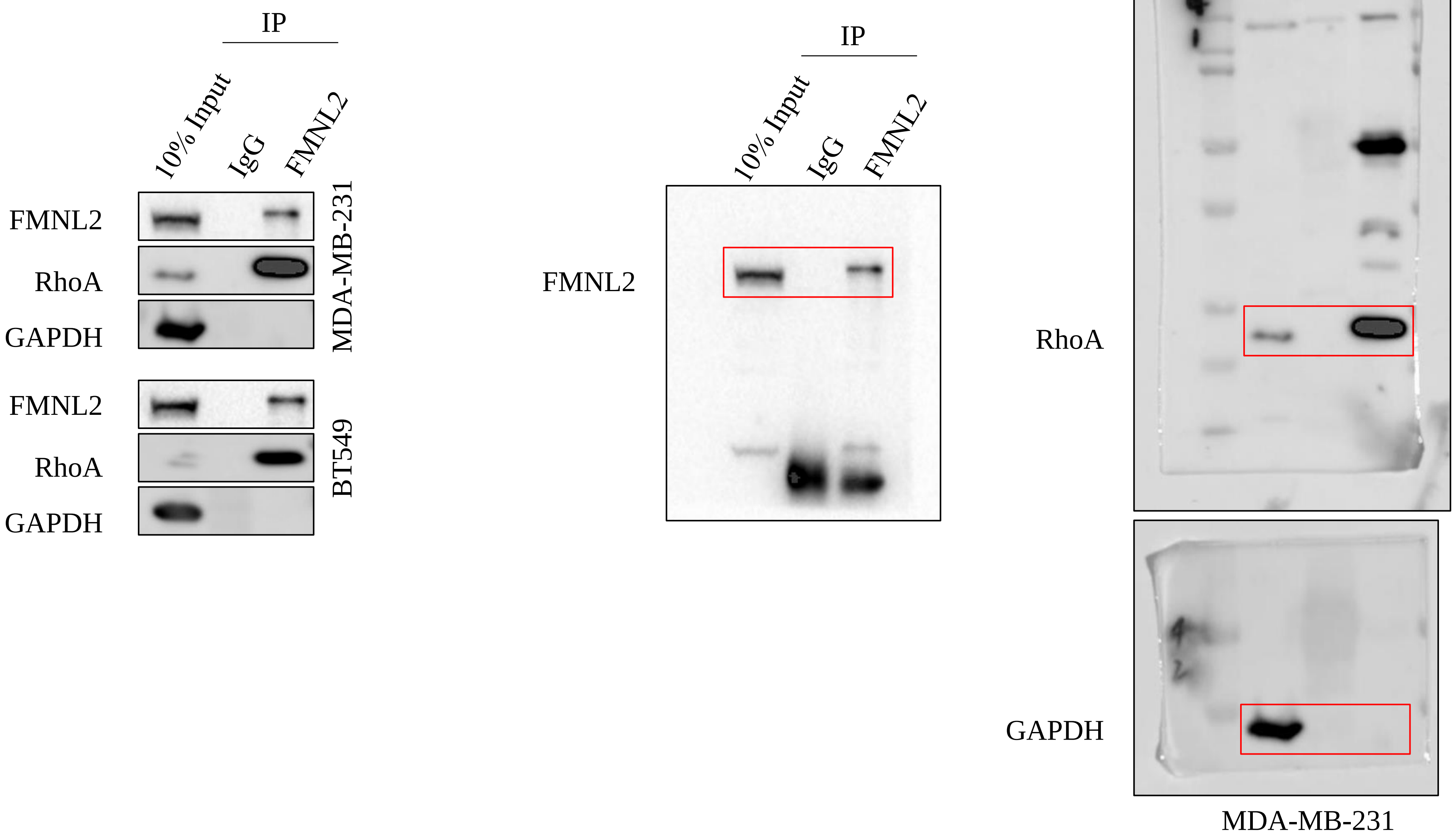
IP
10% Input
IgG
FMNL2
FMNL2
RhoA
GAPDH
MDA-MB-231
FMNL2
RhoA
GAPDH
BT549
IP
10% Input
IgG
FMNL2
FMNL2
RhoA
GAPDH
MDA-MB-231

Fig. 3D

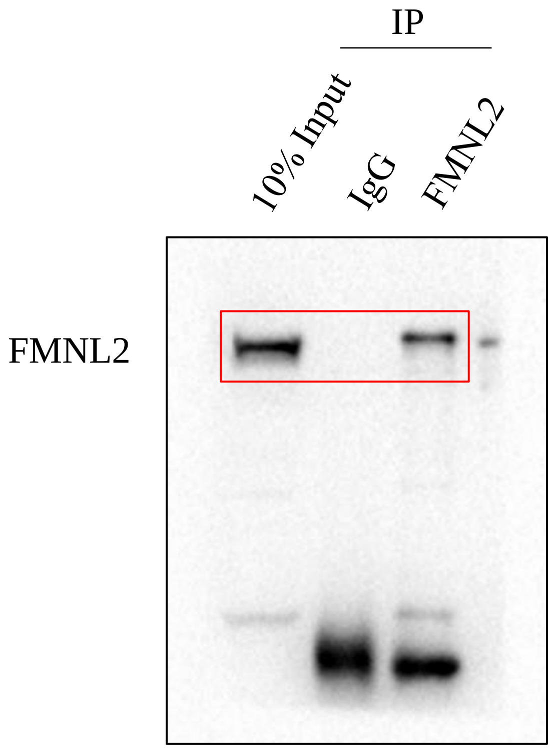

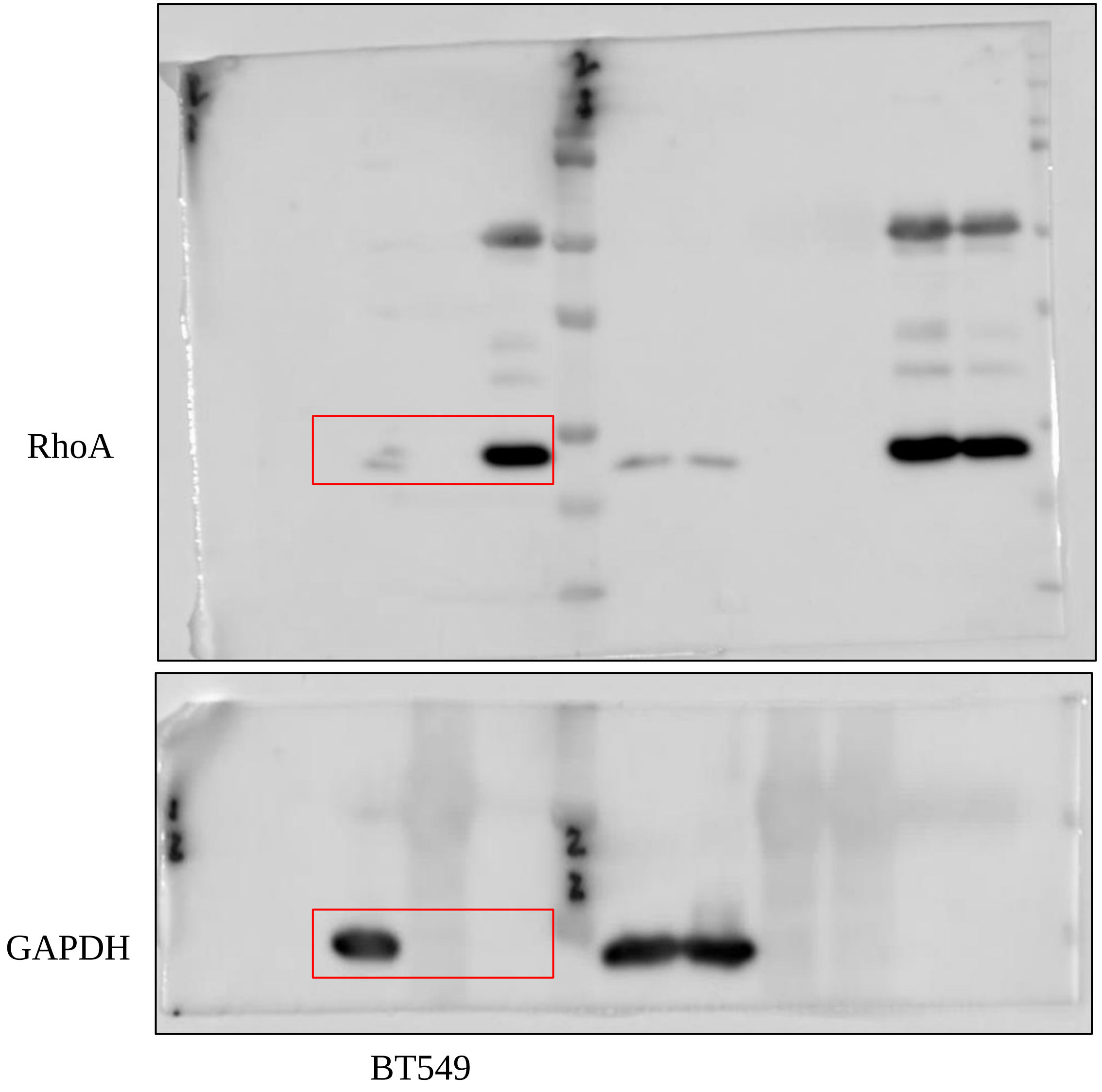

Fig. 4A

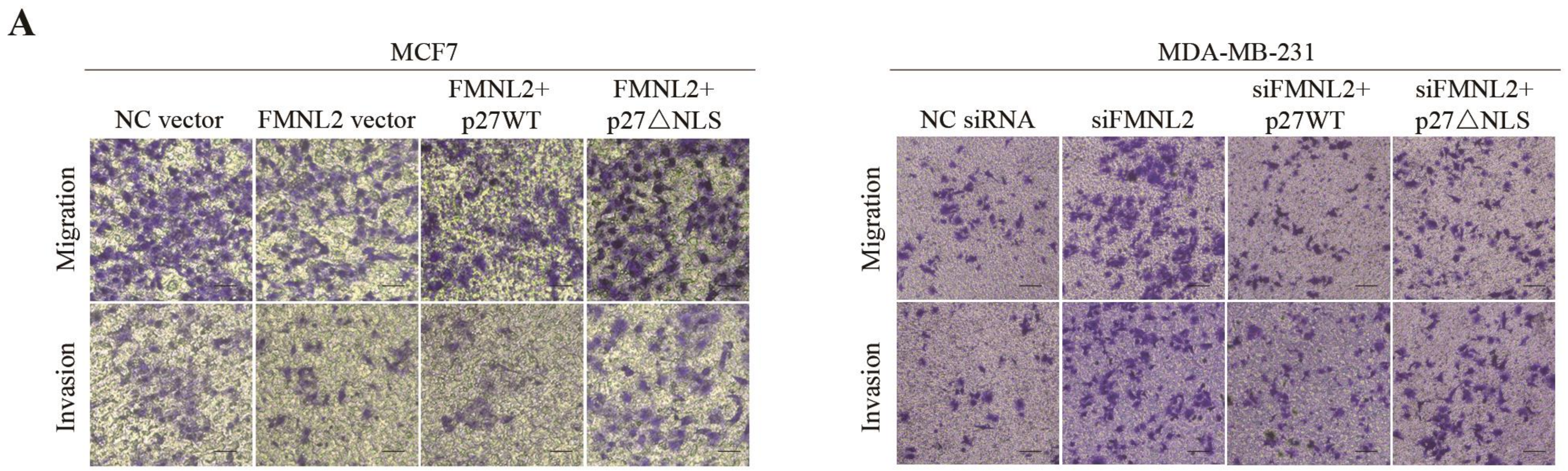
A
MCF7
NC vector
FMNL2 vector
FMNL2+
p27WT
FMNL2+
p27△NLS
Migration
Invasion
MDA-MB-231
NC siRNA
siFMNL2
siFMNL2+
p27WT
siFMNL2+
p27△NLS
Migration
Invasion

Fig. 4A

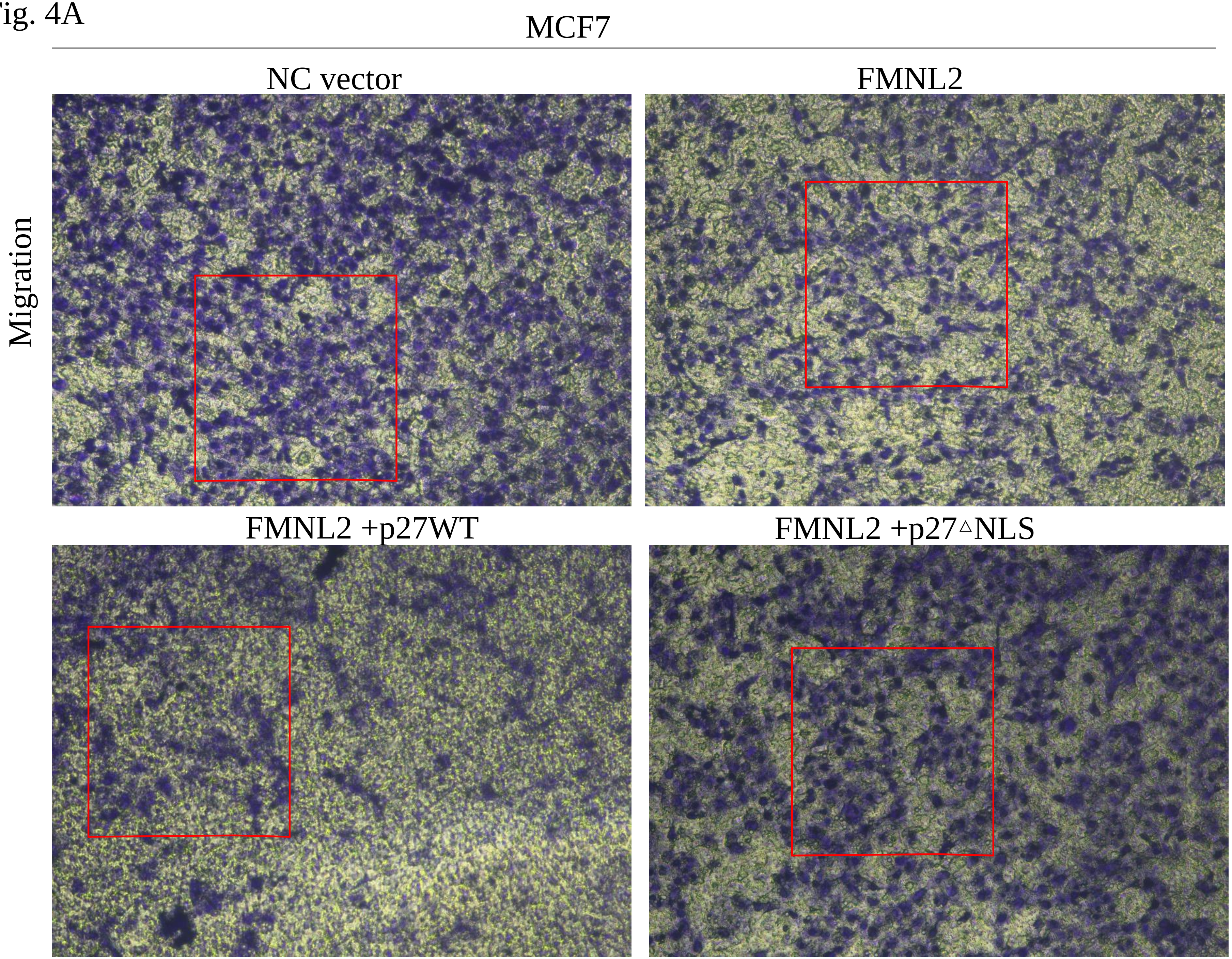

Fig. 4A

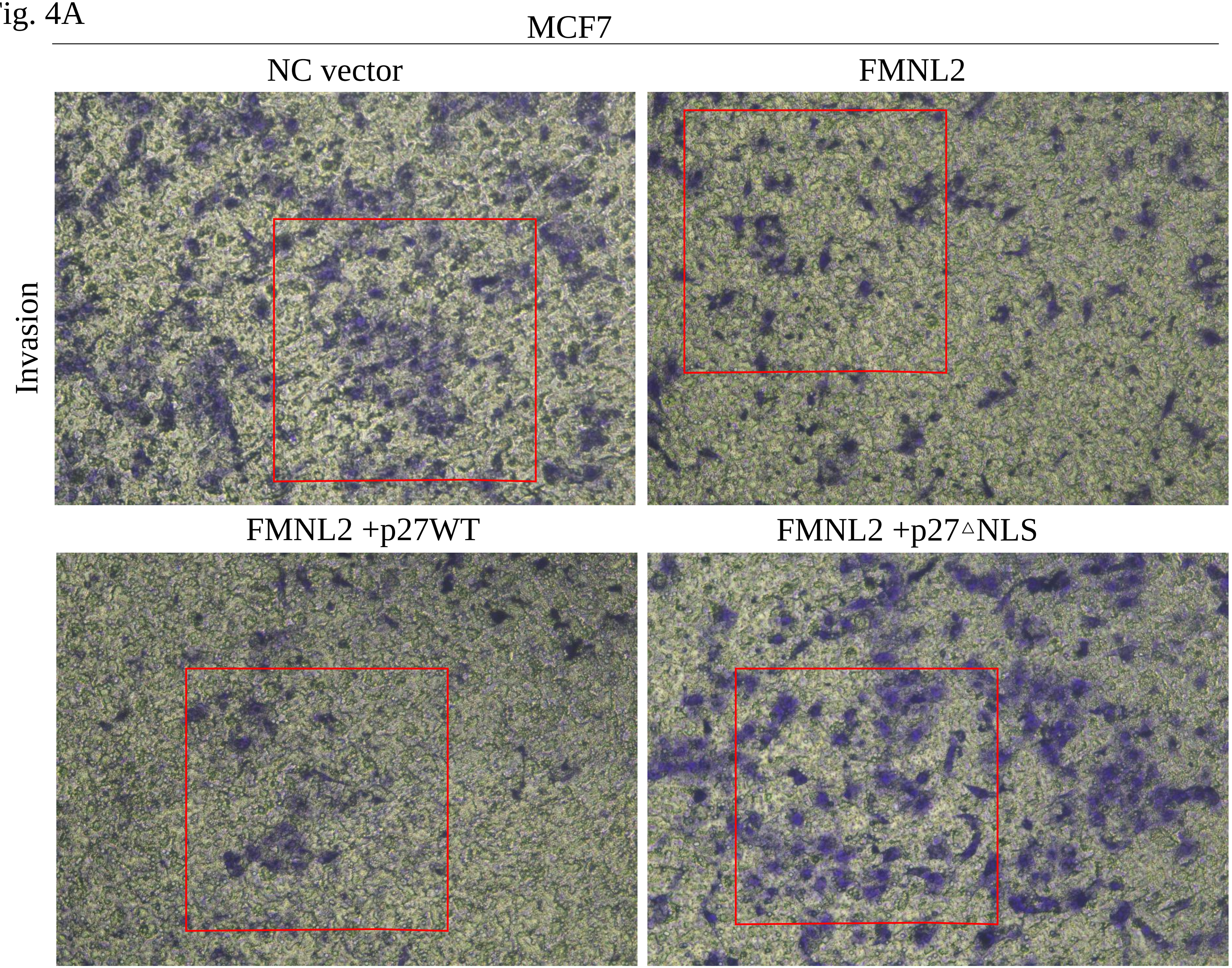

Fig. 4A

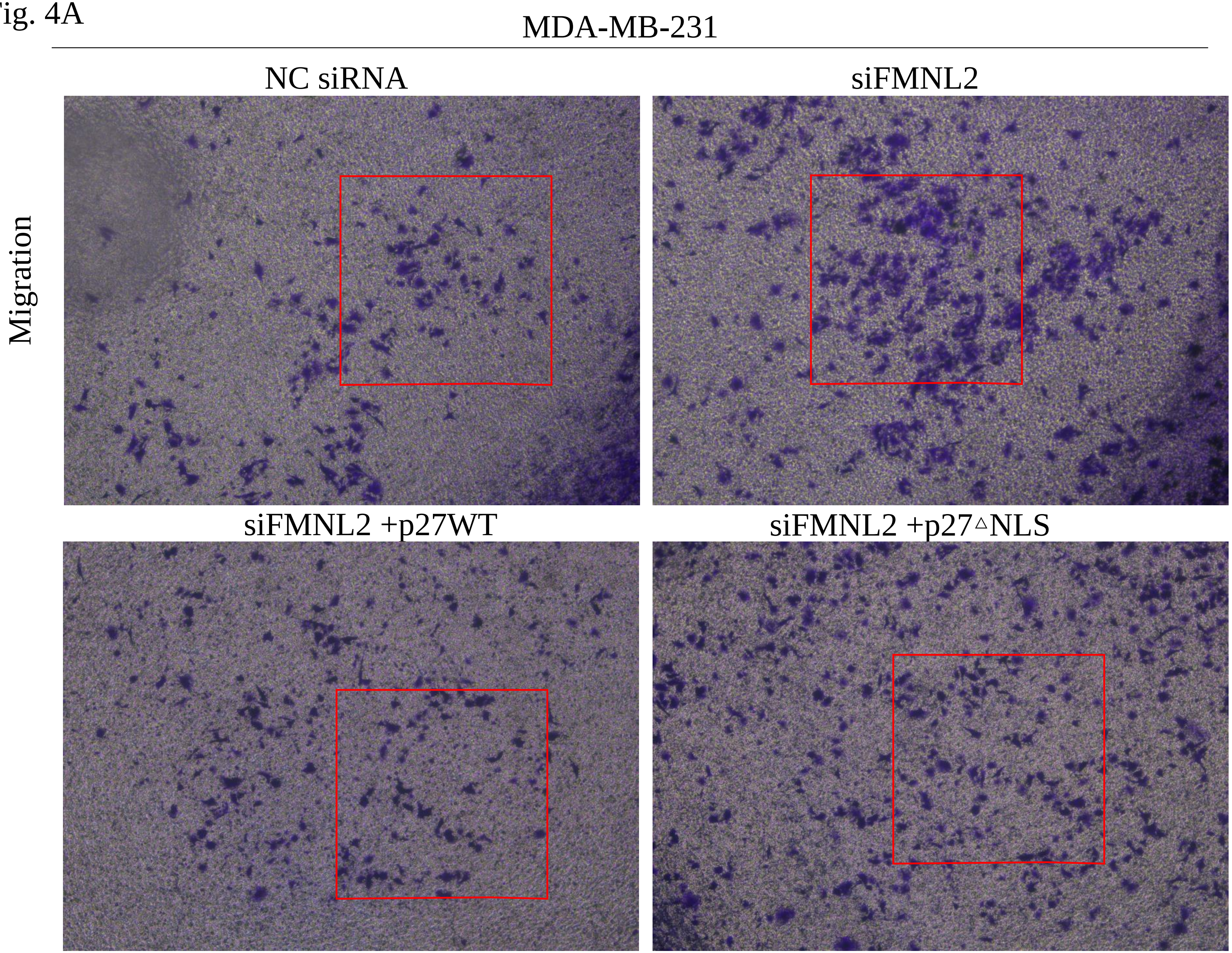
MDA-MB-231
NC siRNA
siFMNL2
Migration
siFMNL2 +p27WT
siFMNL2 +p27△NLS

Fig. 4A

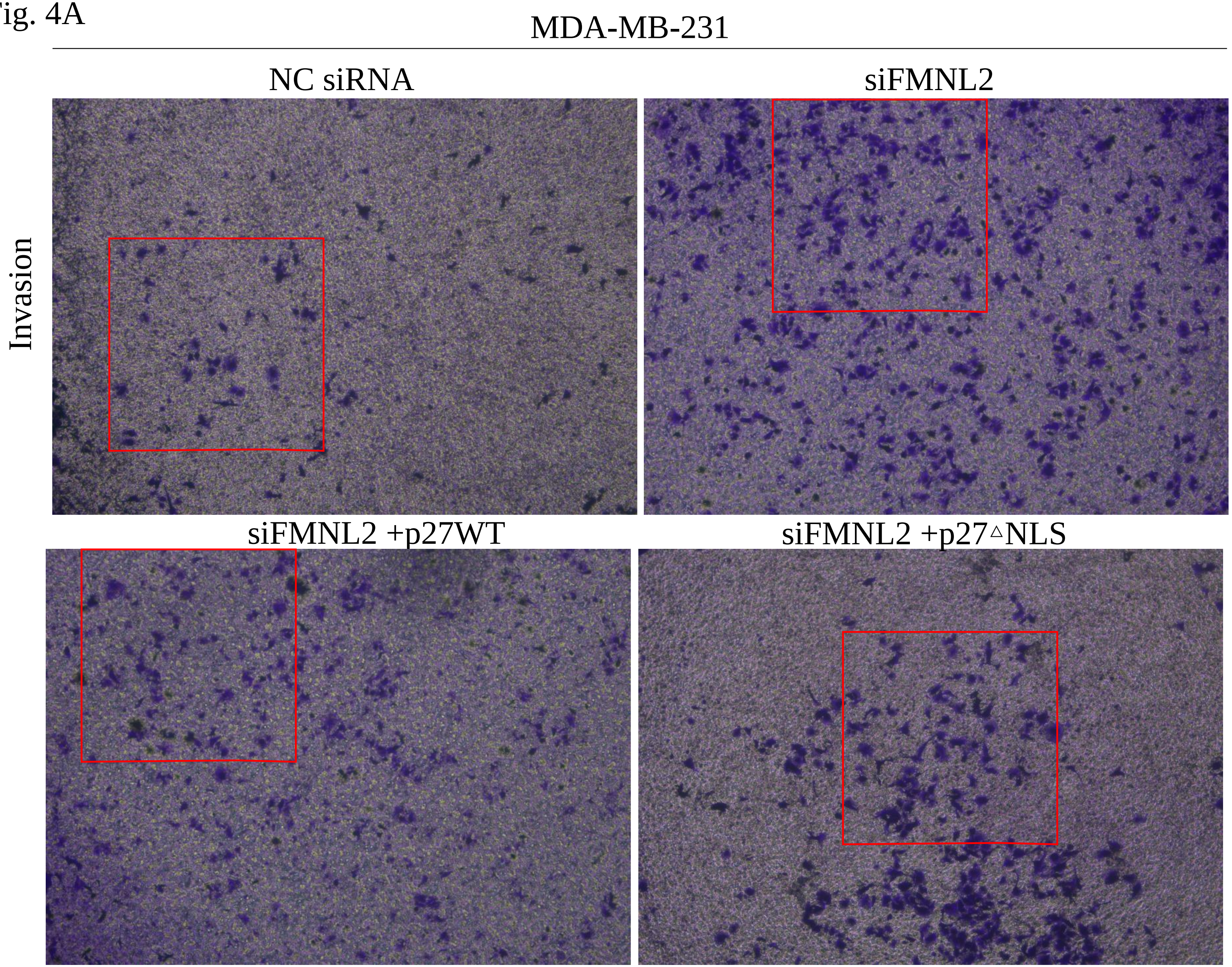
MDA-MB-231
NC siRNA
siFMNL2
Invasion
siFMNL2 +p27WT
siFMNL2 +p27△NLS

Fig. 4C

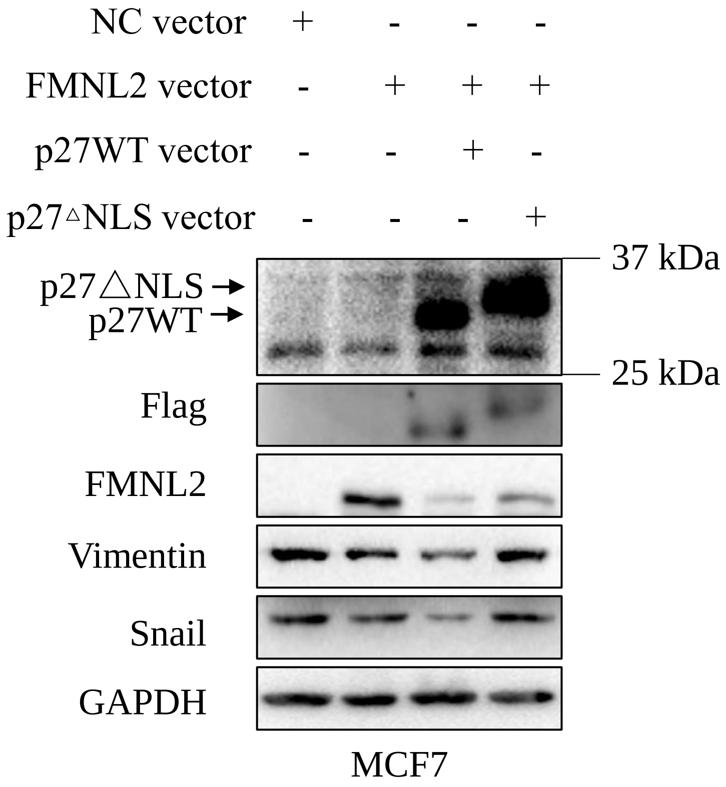
NC vector + - - -
FMNL2 vector - + + +
p27WT vector - - + -
p27△NLS vector - - - +
p27△NLS
p27WT
37 kDa
25 kDa
Flag
FMNL2
Vimentin
Snail
GAPDH
MCF7

Fig. 4C

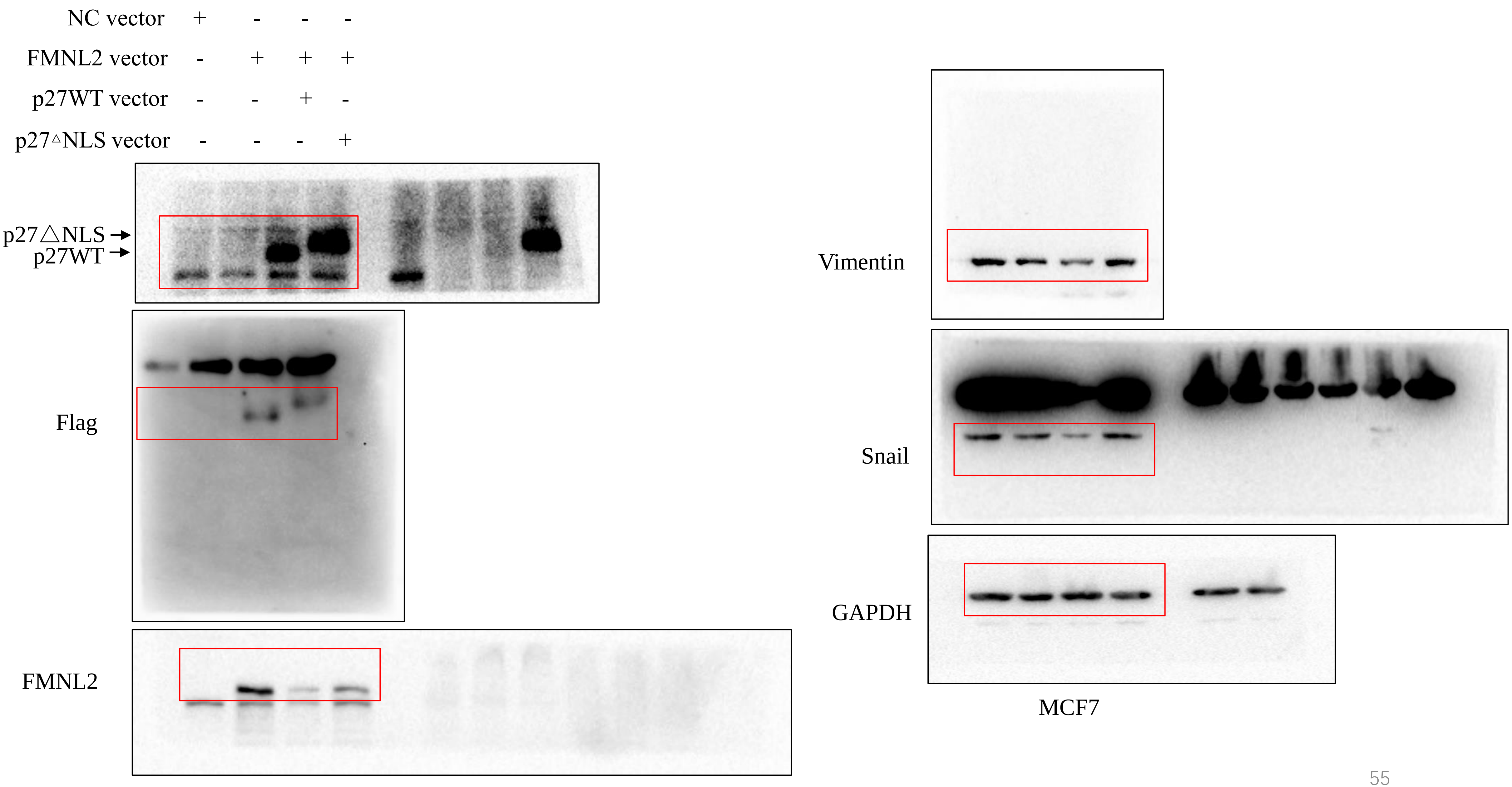
NC vector + - - -
FMNL2 vector - + + +
p27WT vector - - + -
p27△NLS vector - - - +
p27△NLS
p27WT
Flag
FMNL2
Vimentin
Snail
GAPDH
MCF7

Fig. 4C

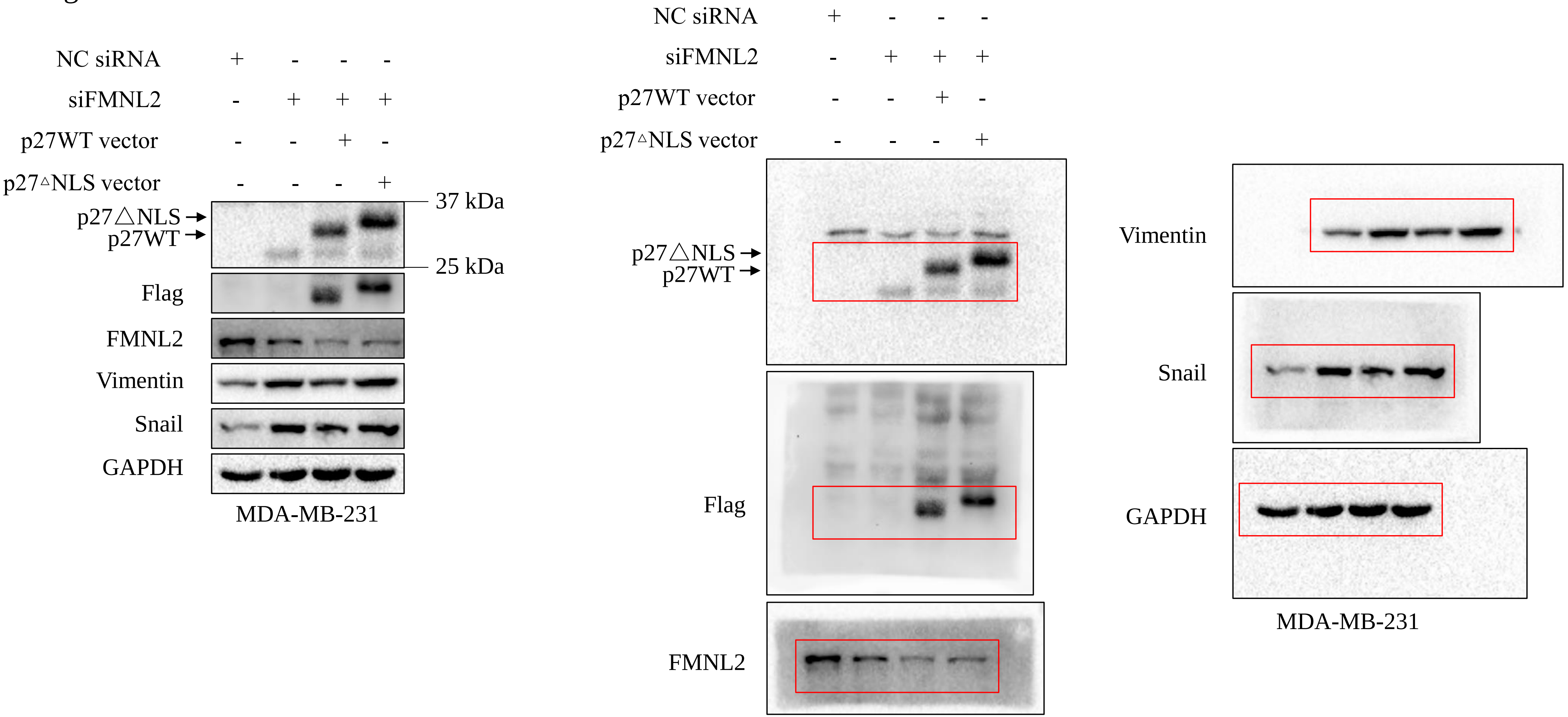
NC siRNA + - - -
siFMNL2 - + + +
p27WT vector - - + -
p27△NLS vector - - - +
p27△NLS
p27WT
37 kDa
25 kDa
Flag
FMNL2
Vimentin
Snail
GAPDH
MDA-MB-231
NC siRNA + - - -
siFMNL2 - + + +
p27WT vector - - + -
p27△NLS vector - - - +
p27△NLS
p27WT
Flag
FMNL2
Vimentin
Snail
GAPDH
MDA-MB-231

Fig. 4D

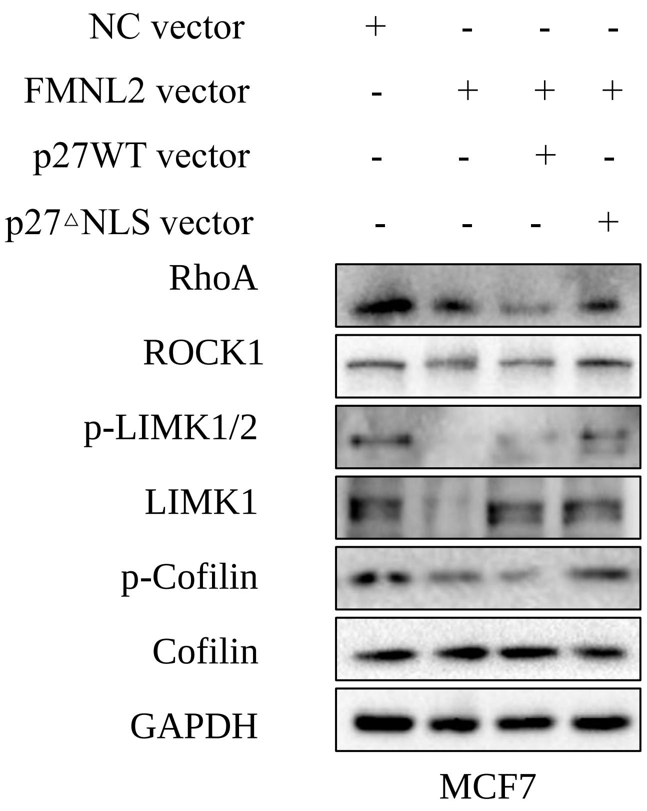
NC vector + - - -
FMNL2 vector - + + +
p27WT vector - - + -
p27△NLS vector - - - +
RhoA
ROCK1
p-LIMK1/2
LIMK1
p-Cofilin
Cofilin
GAPDH
MCF7

Fig. 4D

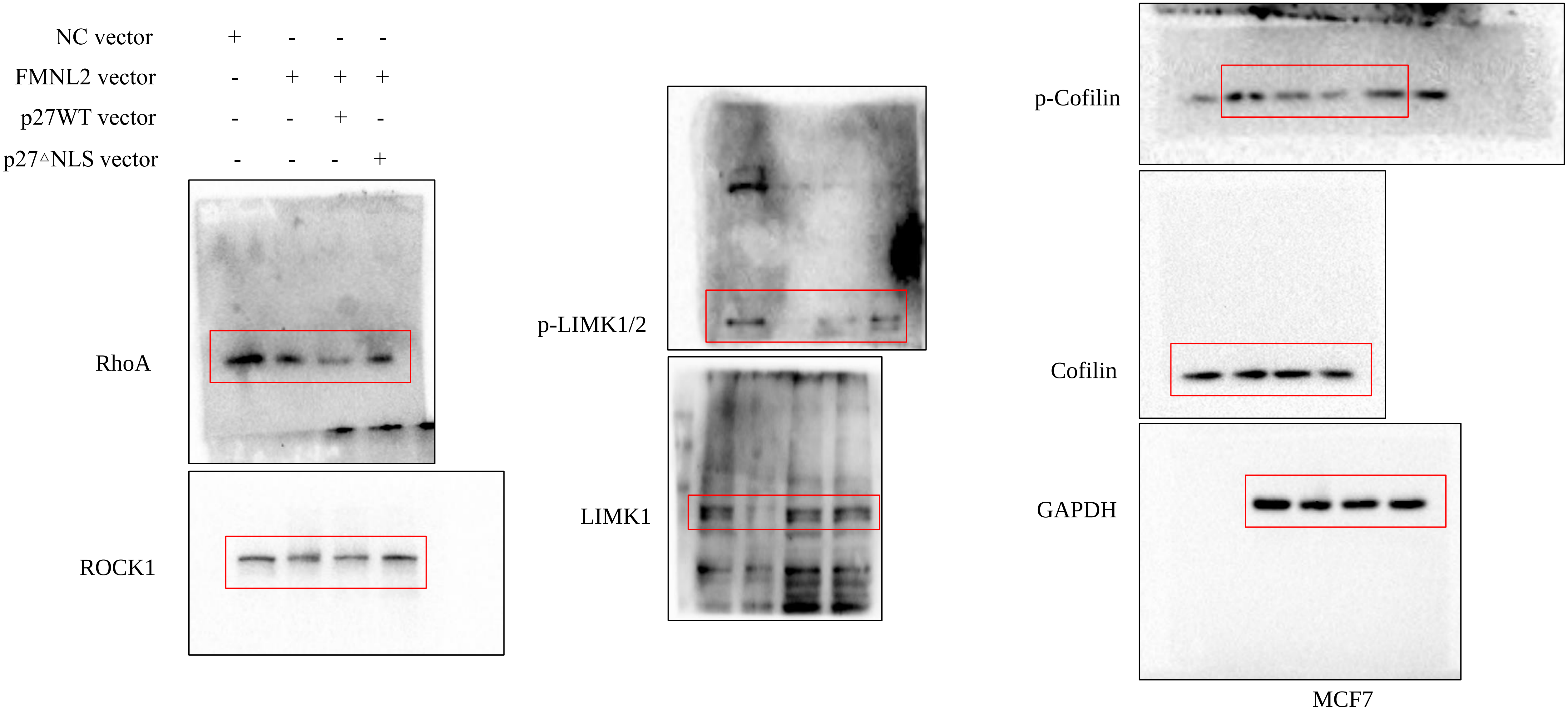
NC vector + - - -
FMNL2 vector - + + +
p27WT vector - - + -
p27△NLS vector - - - +
RhoA
ROCK1
p-LIMK1/2
LIMK1
p-Cofilin
Cofilin
GAPDH
MCF7

Fig. 4D

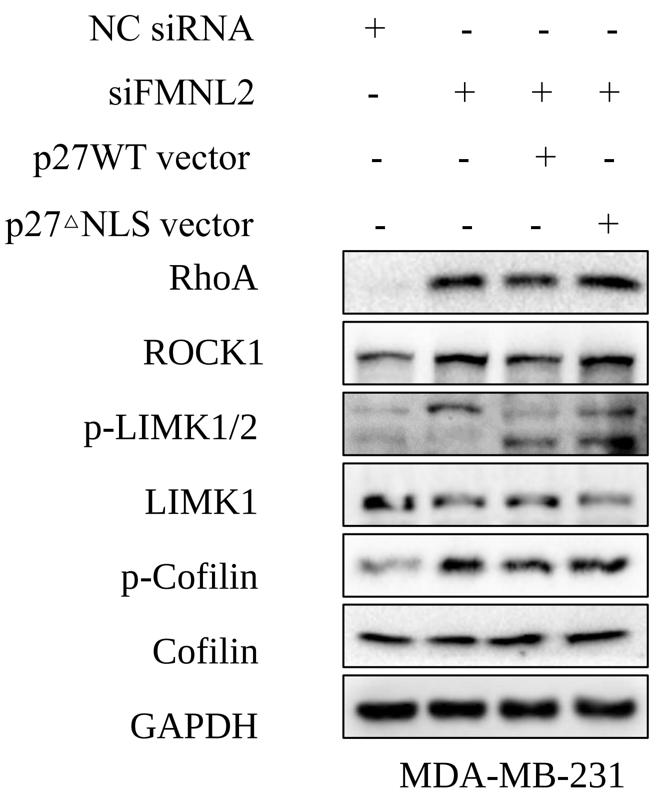
NC siRNA + - - -
siFMNL2 - + + +
p27WT vector - - + -
p27△NLS vector - - - +
RhoA
ROCK1
p-LIMK1/2
LIMK1
p-Cofilin
Cofilin
GAPDH
MDA-MB-231

Fig. 4D

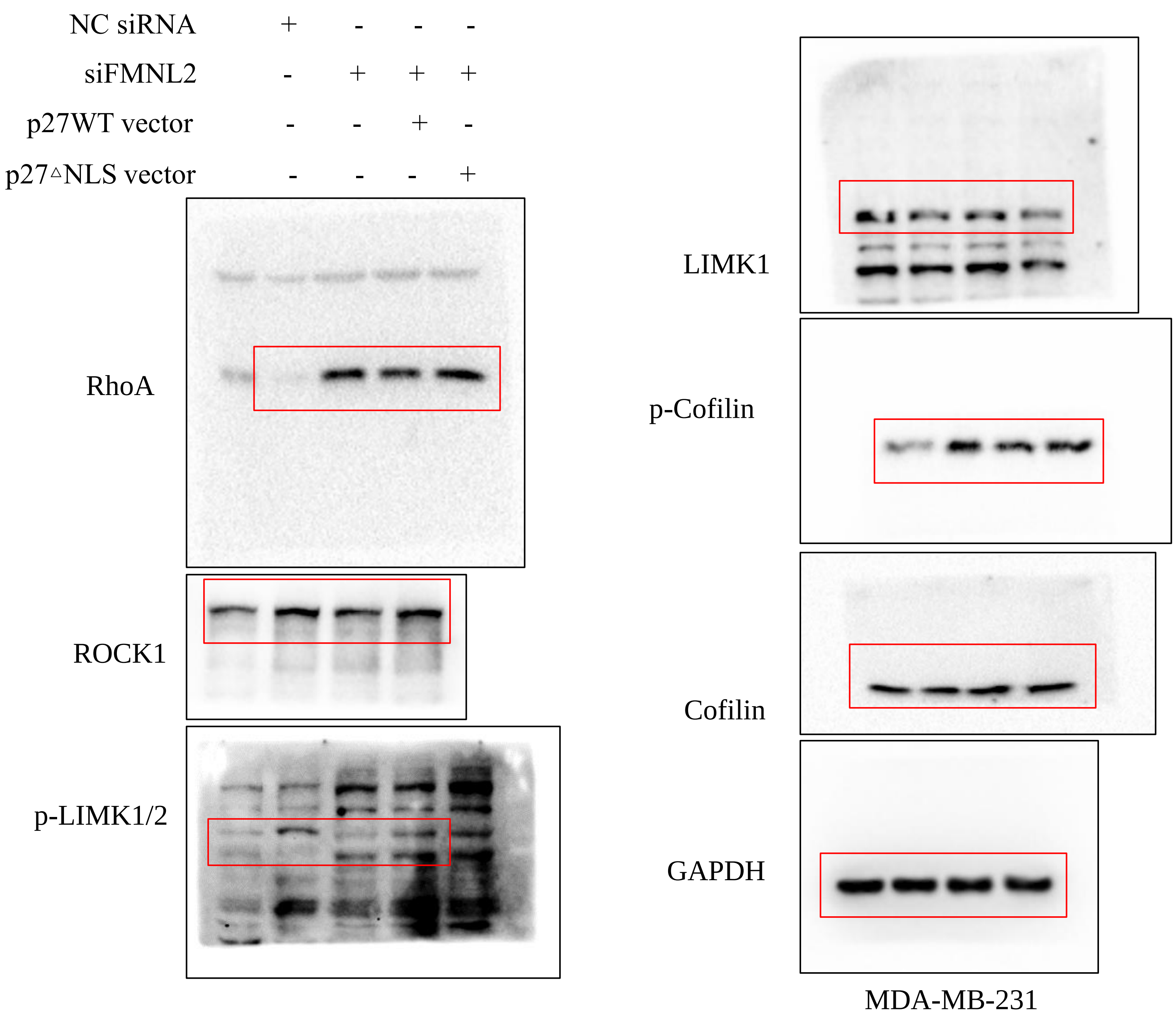

NC siRNA + - - -
siFMNL2 - + + +
p27WT vector - - + -
p27△NLS vector - - - +
RhoA
ROCK1
p-LIMK1/2
LIMK1
p-Cofilin
Cofilin
GAPDH
MDA-MB-231

Fig. 5D

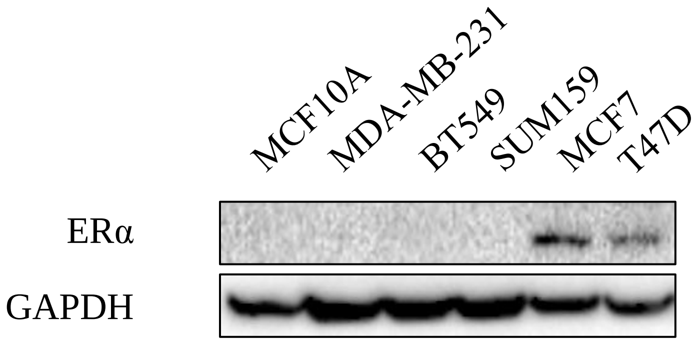
MCF10A
MDA-MB-231
BT549
SUM159
MCF7
T47D
ERα
GAPDH

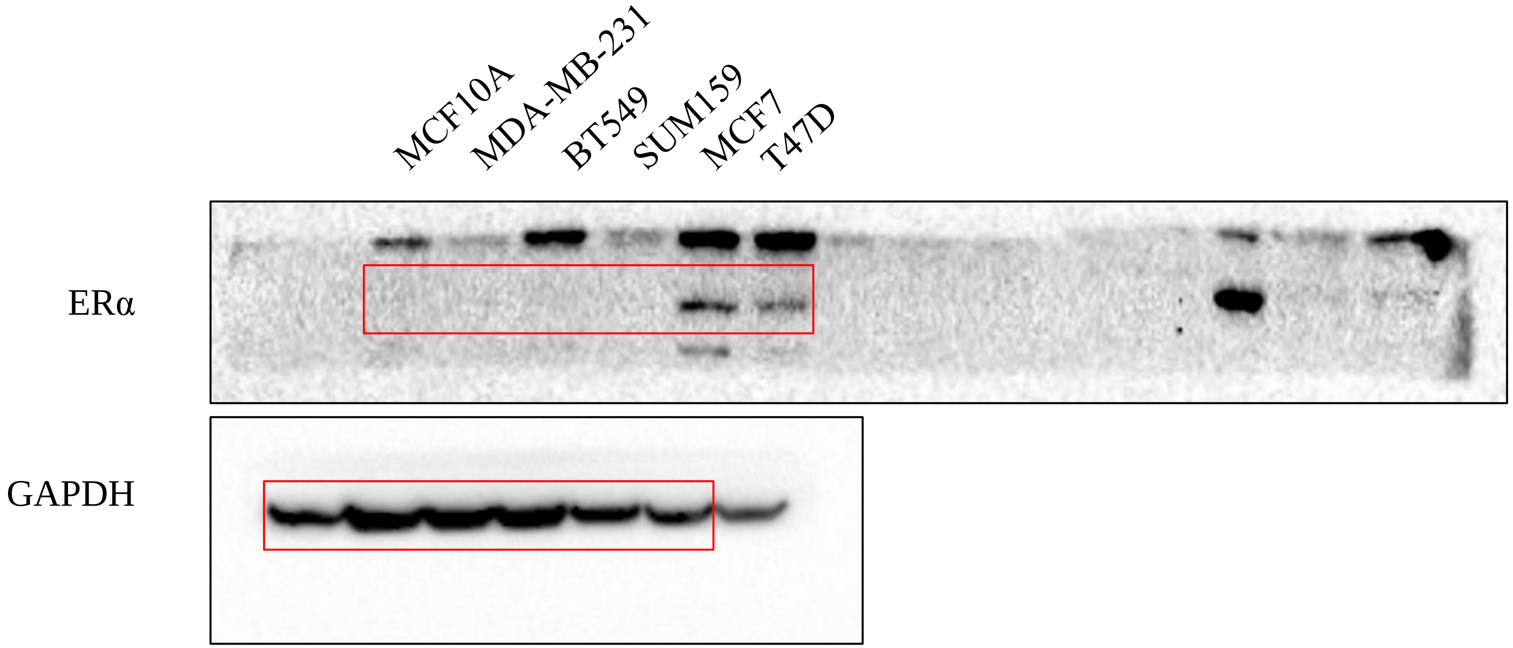
MCF10A
MDA-MB-231
BT549
SUM159
MCF7
T47D
ERα
GAPDH

Fig. 5F

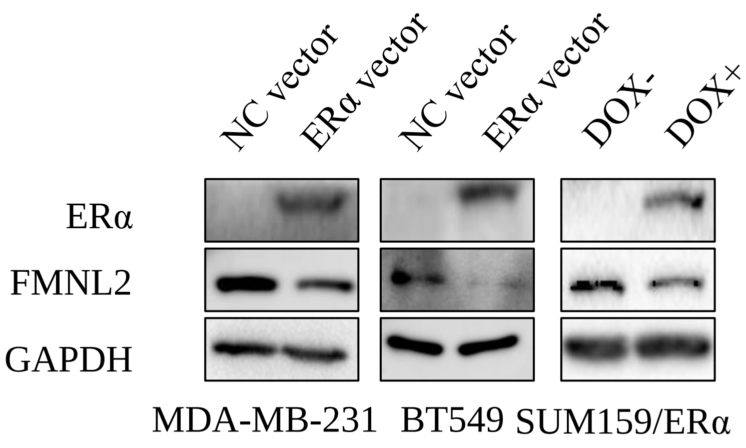
NC vector
ERα vector
NC vector
ERα vector
DOX-
DOX+
ERα
FMNL2
GAPDH
MDA-MB-231
BT549
SUM159/ERα

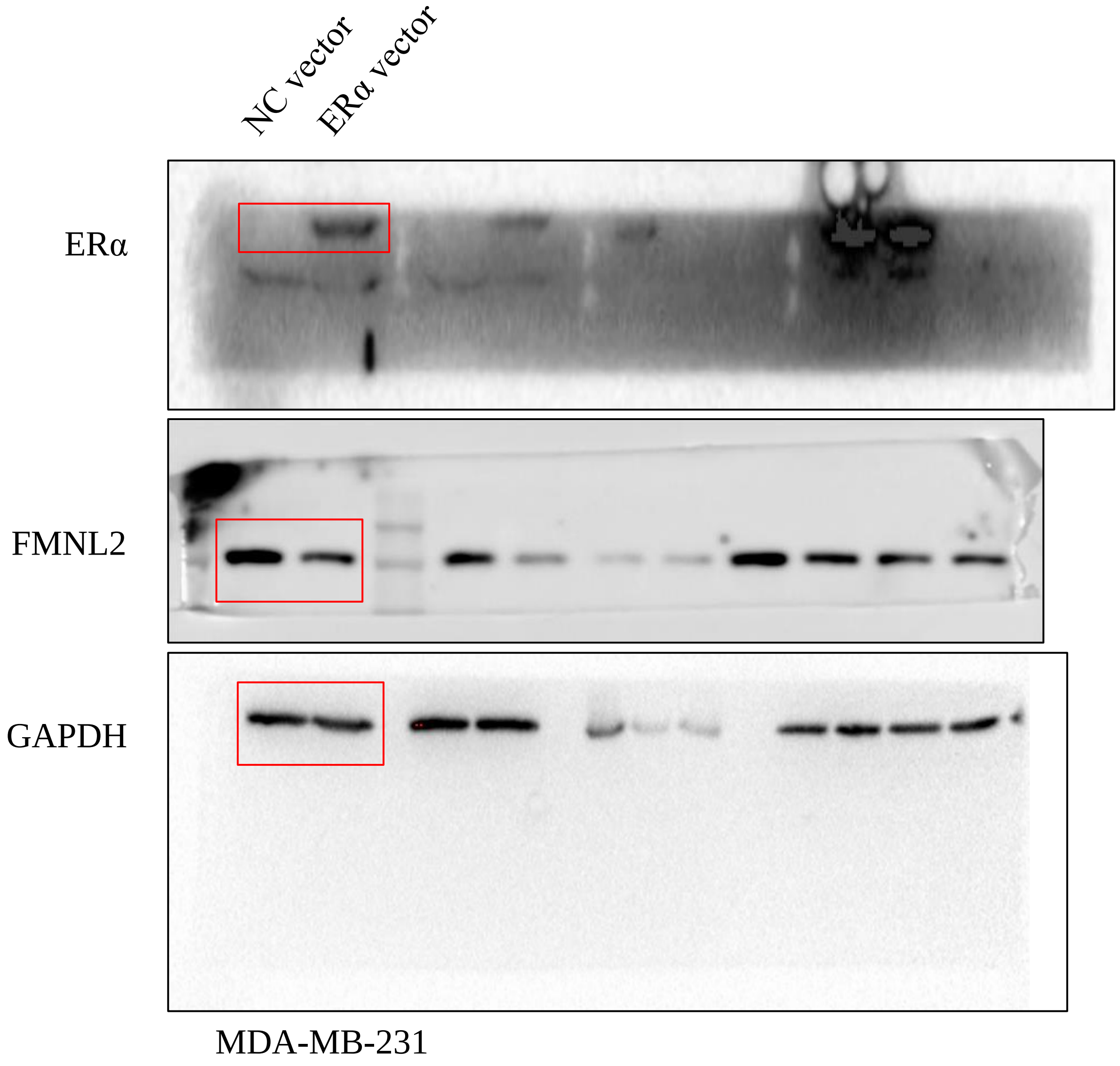
NC vector
ERα vector
ERα
FMNL2
GAPDH
MDA-MB-231

Fig. 5F

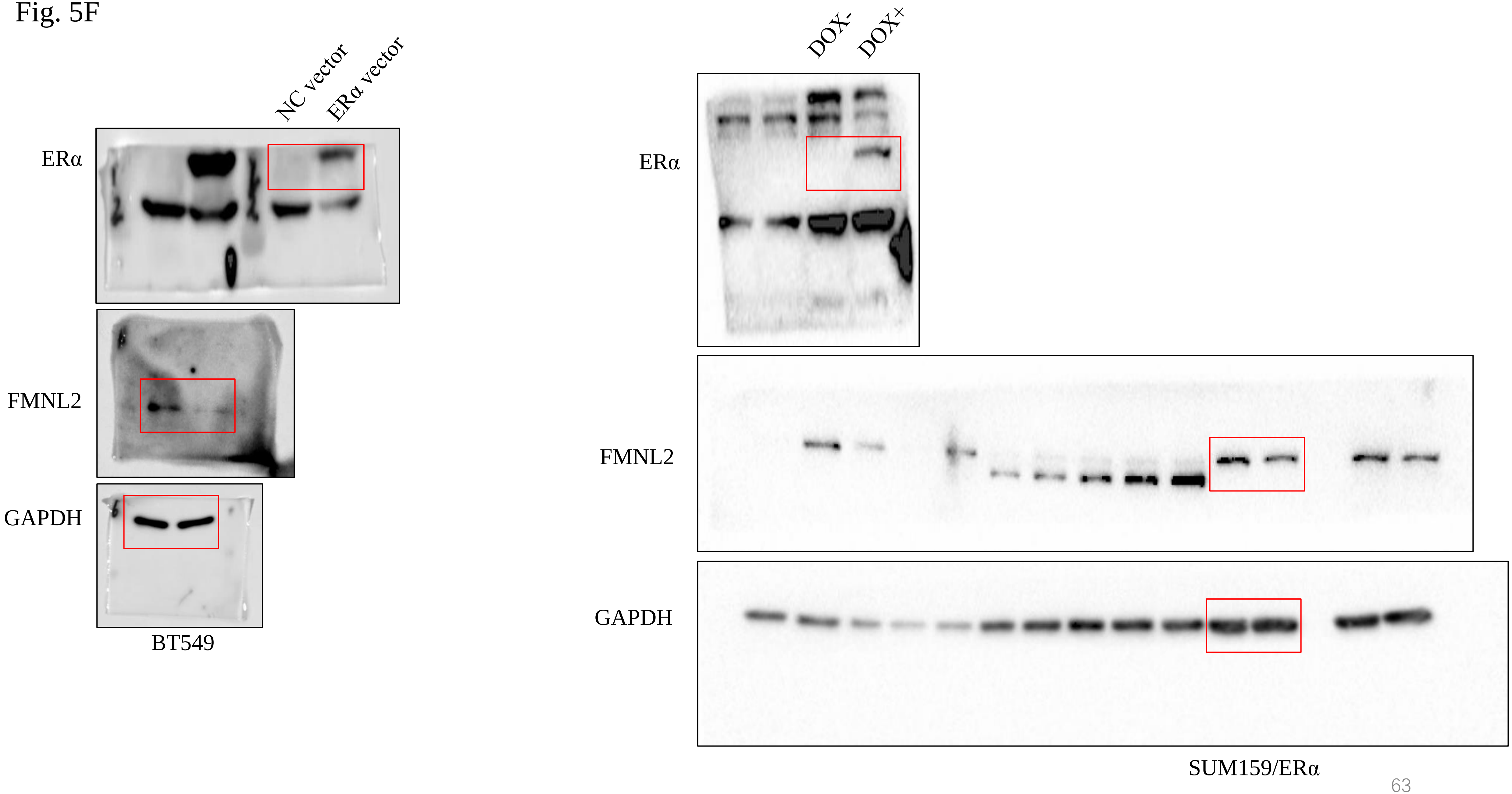
NC vector
ERα vector
ERα
FMNL2
GAPDH
BT549
DOX-
DOX+
ERα
FMNL2
GAPDH
SUM159/ERα

Fig. 5G

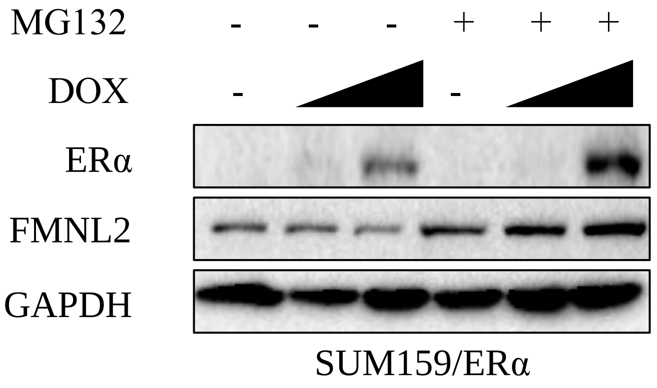
MG132
- - - + + +
DOX
-
-
ERα
FMNL2
GAPDH
SUM159/ERα

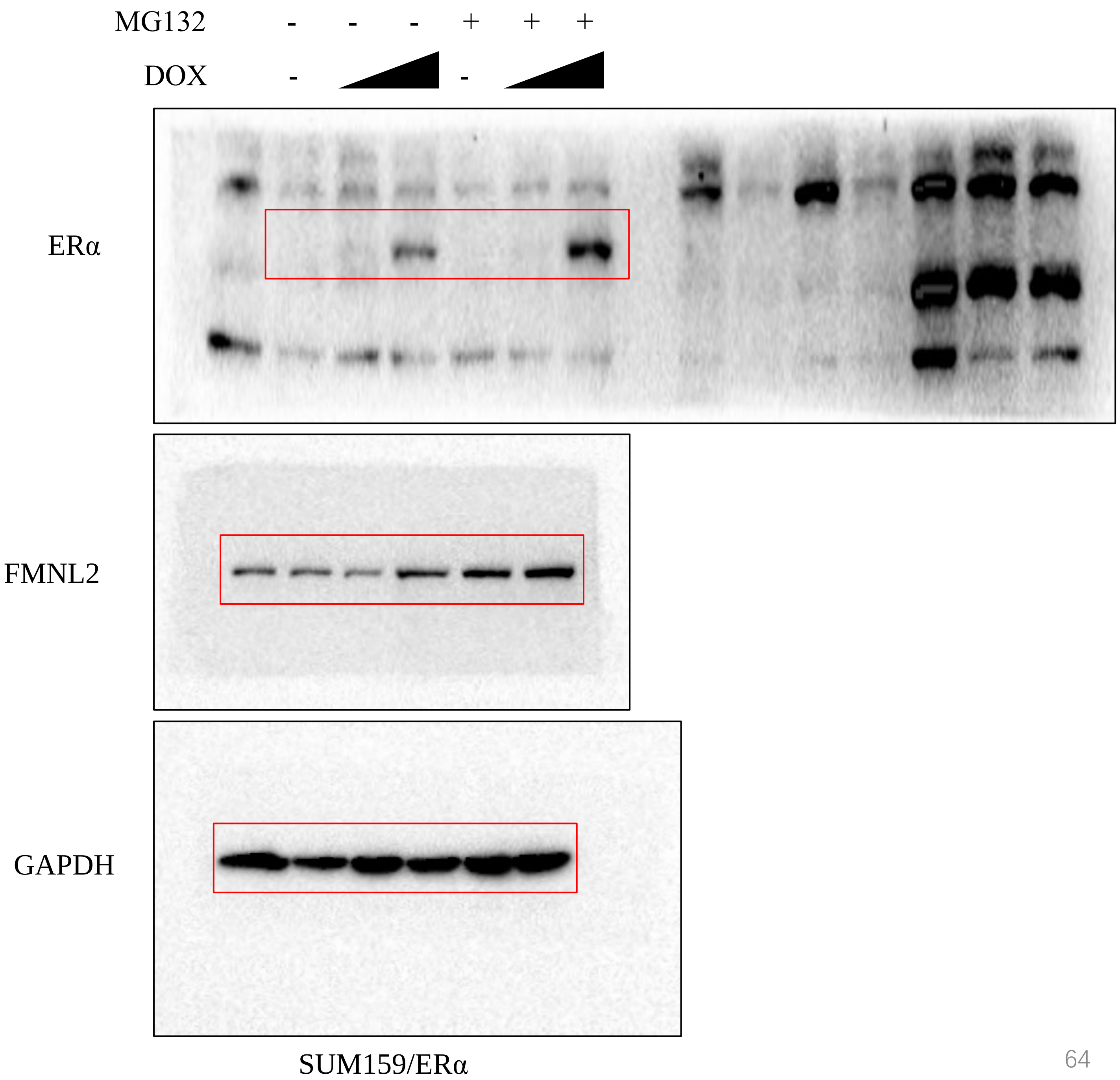
MG132
- - - + + +
DOX
-
-
ERα
FMNL2
GAPDH
SUM159/ERα

Fig. S1D

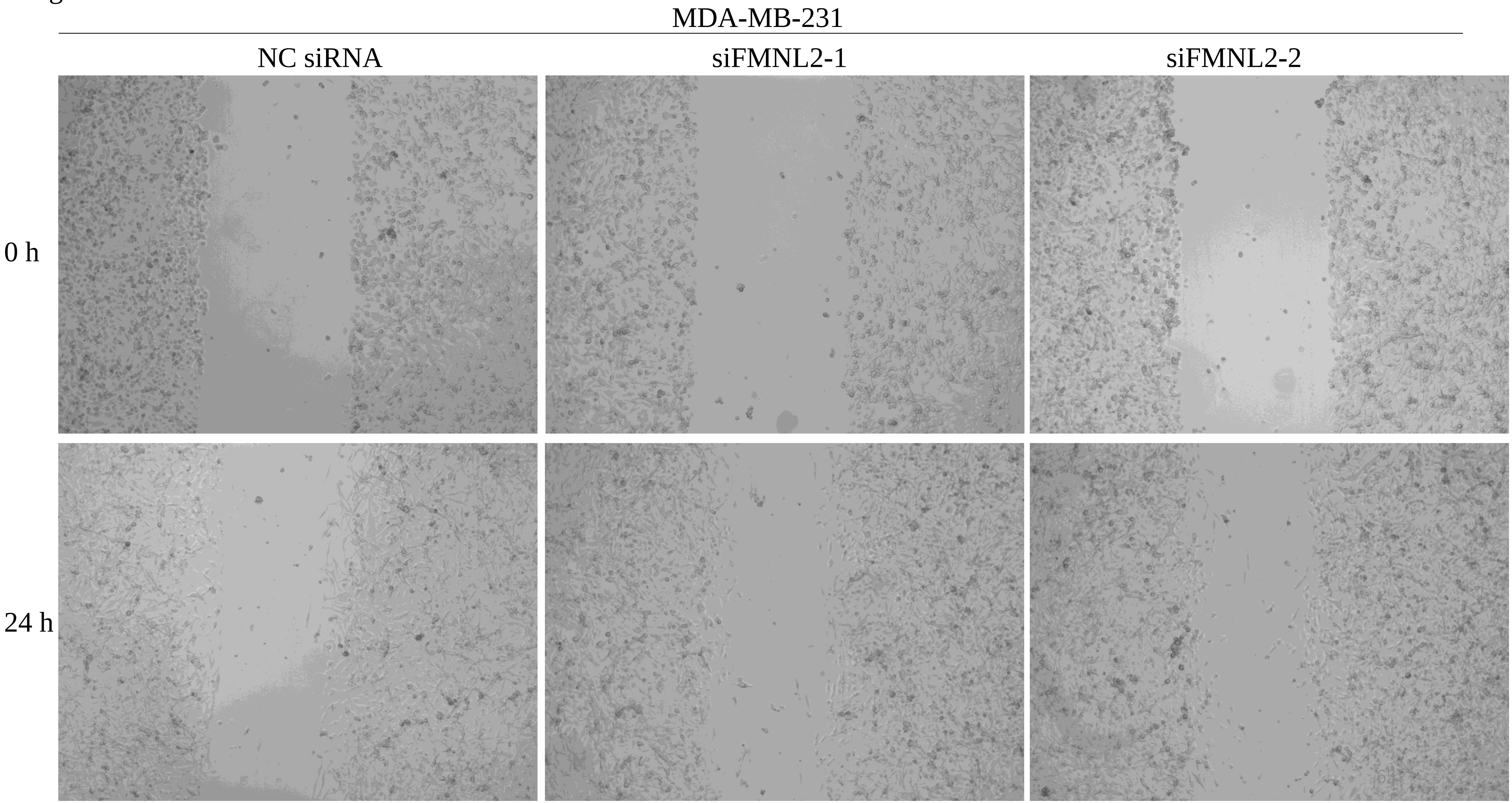

Fig. S1D

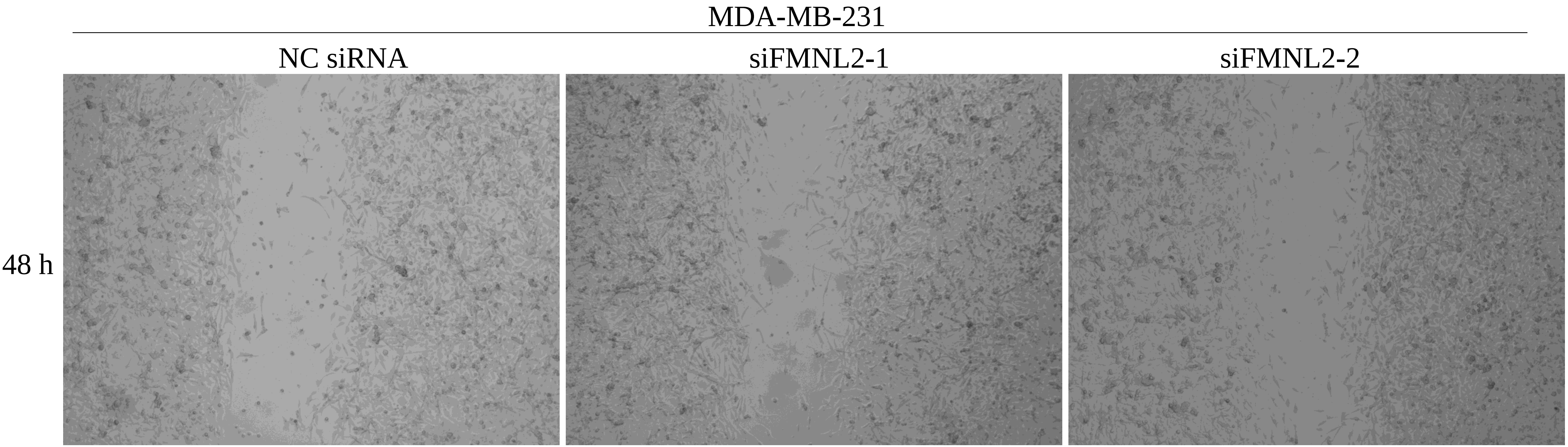

Fig. S2A

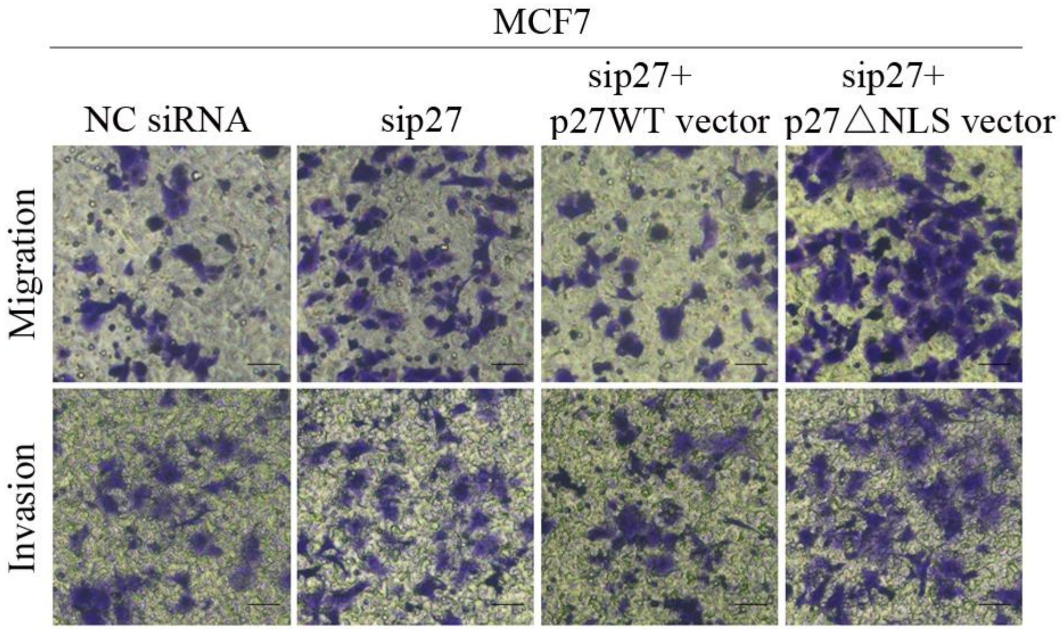
MCF7
NC siRNA
sip27
sip27+
p27WT vector
sip27+
p27△NLS vector
Migration
Invasion

Fig. S2A

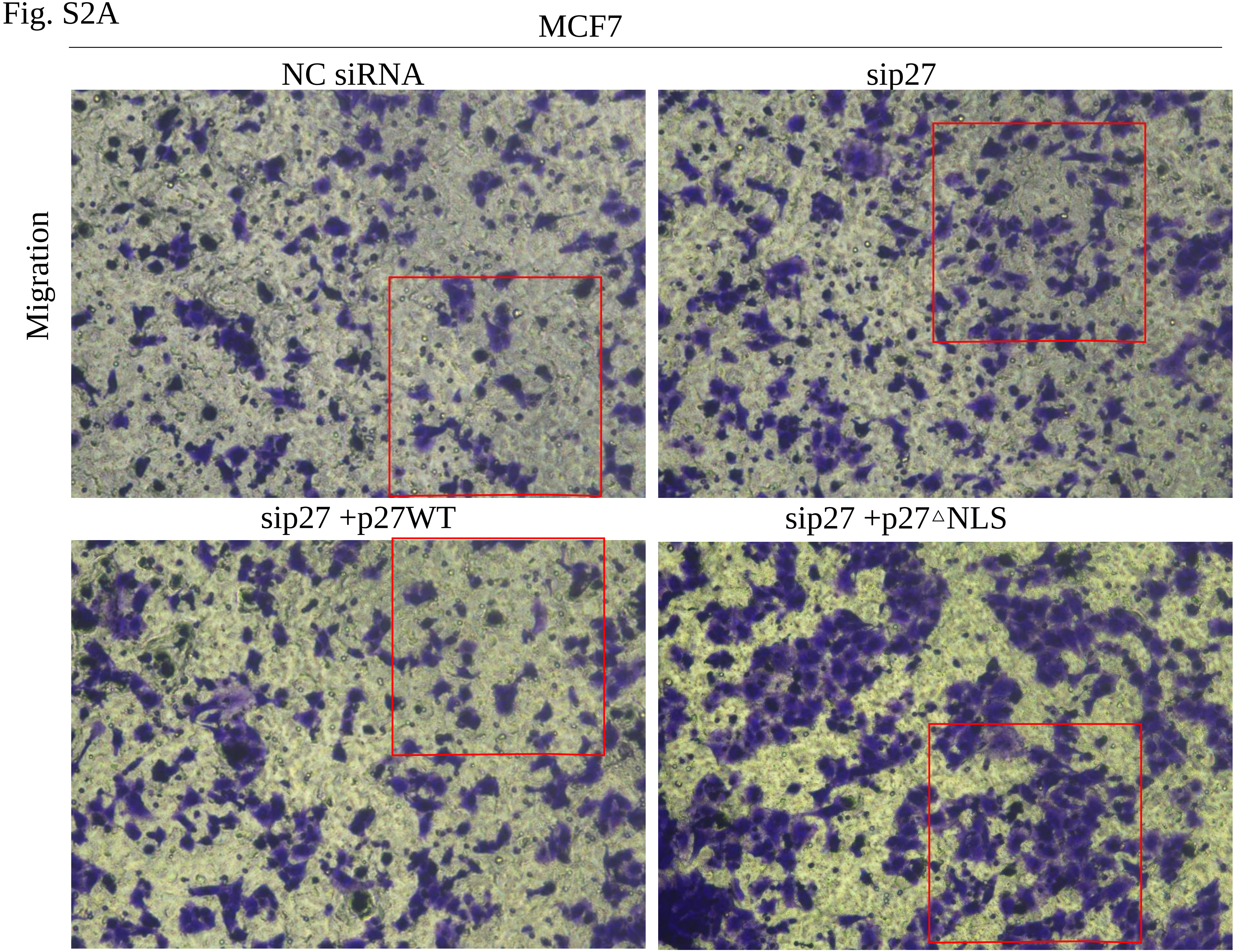

Fig. S2A

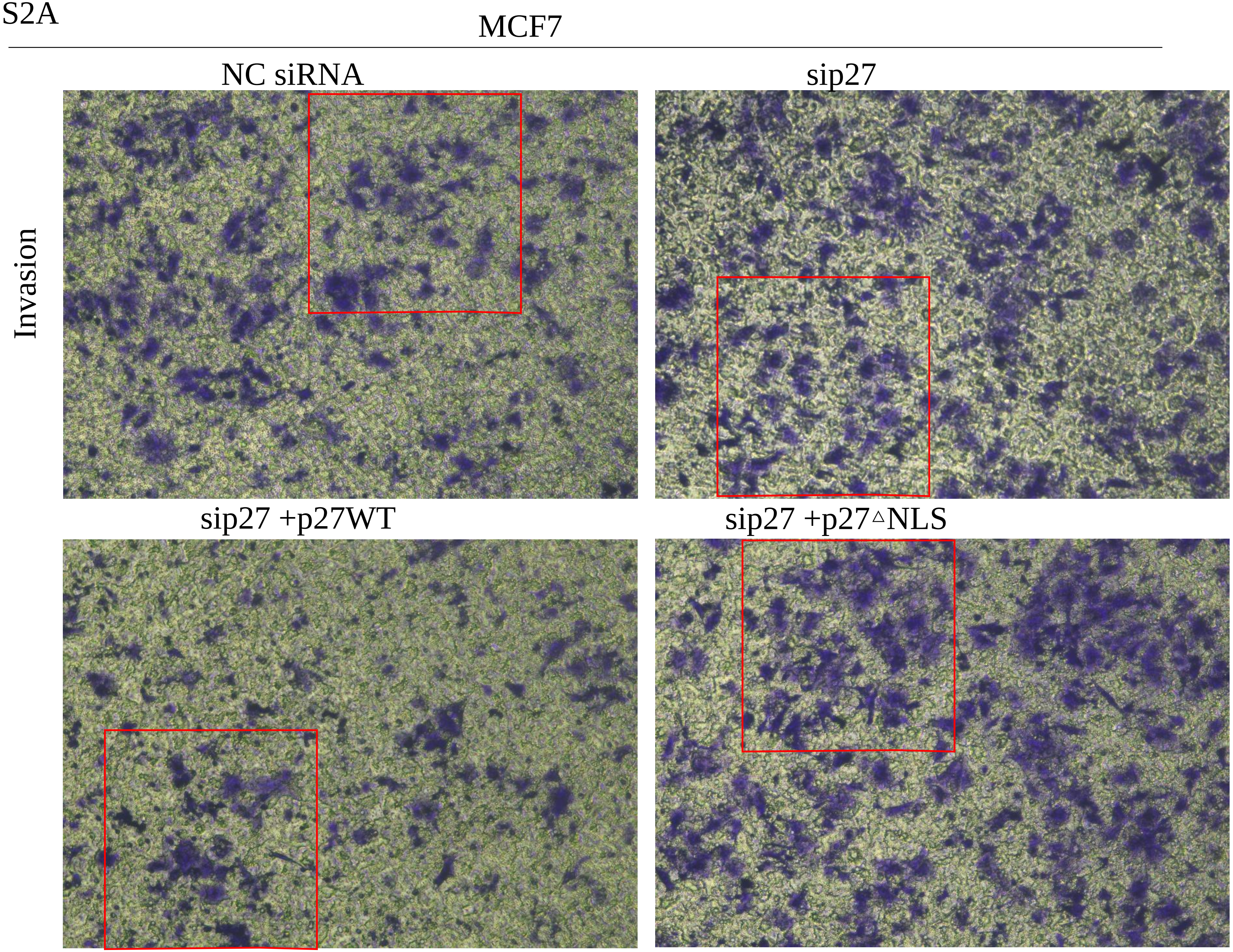

Fig. S2C

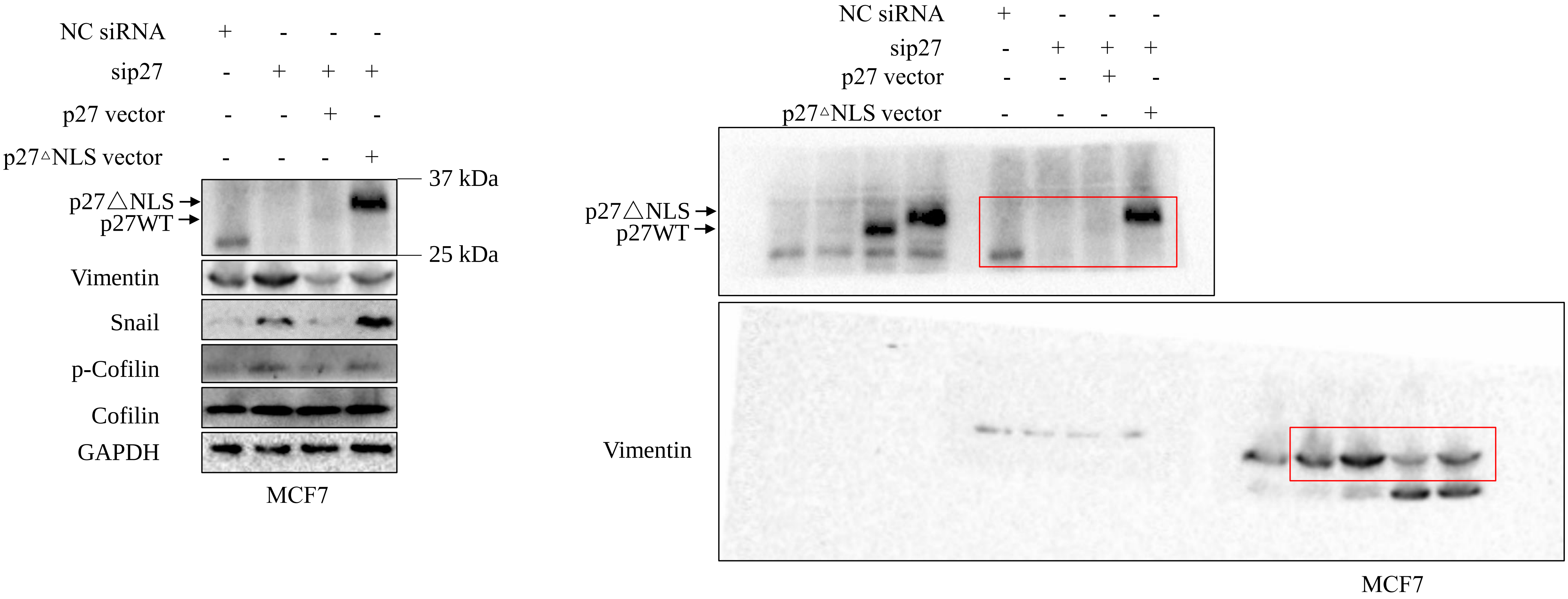
NC siRNA + - - -
sip27 - + + +
p27 vector - - + -
p27△NLS vector - - - +
p27△NLS
p27WT
37 kDa
25 kDa
Vimentin
Snail
p-Cofilin
Cofilin
GAPDH
MCF7
NC siRNA + - - -
sip27 - + + +
p27 vector - - + -
p27△NLS vector - - - +
p27△NLS
p27WT
Vimentin
MCF7

Fig. S2C

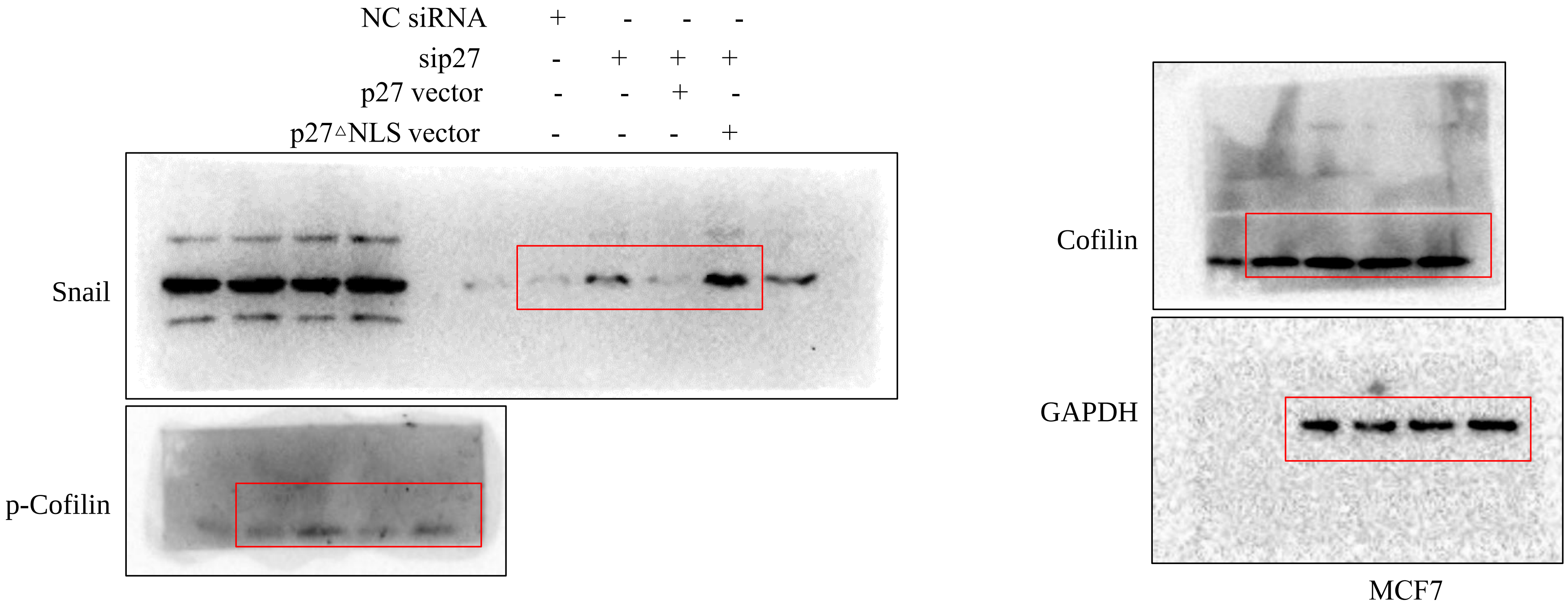
NC siRNA + - - -
sip27 - + + +
p27 vector - - + -
p27△NLS vector - - - +
Snail
p-Cofilin
Cofilin
GAPDH
MCF7

Fig. S2D

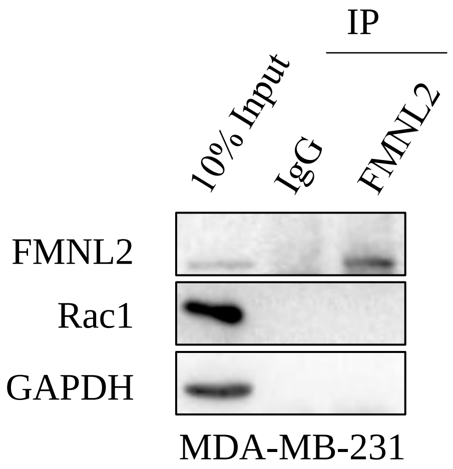
IP
10% Input
IgG
FMNL2
FMNL2
Rac1
GAPDH
MDA-MB-231

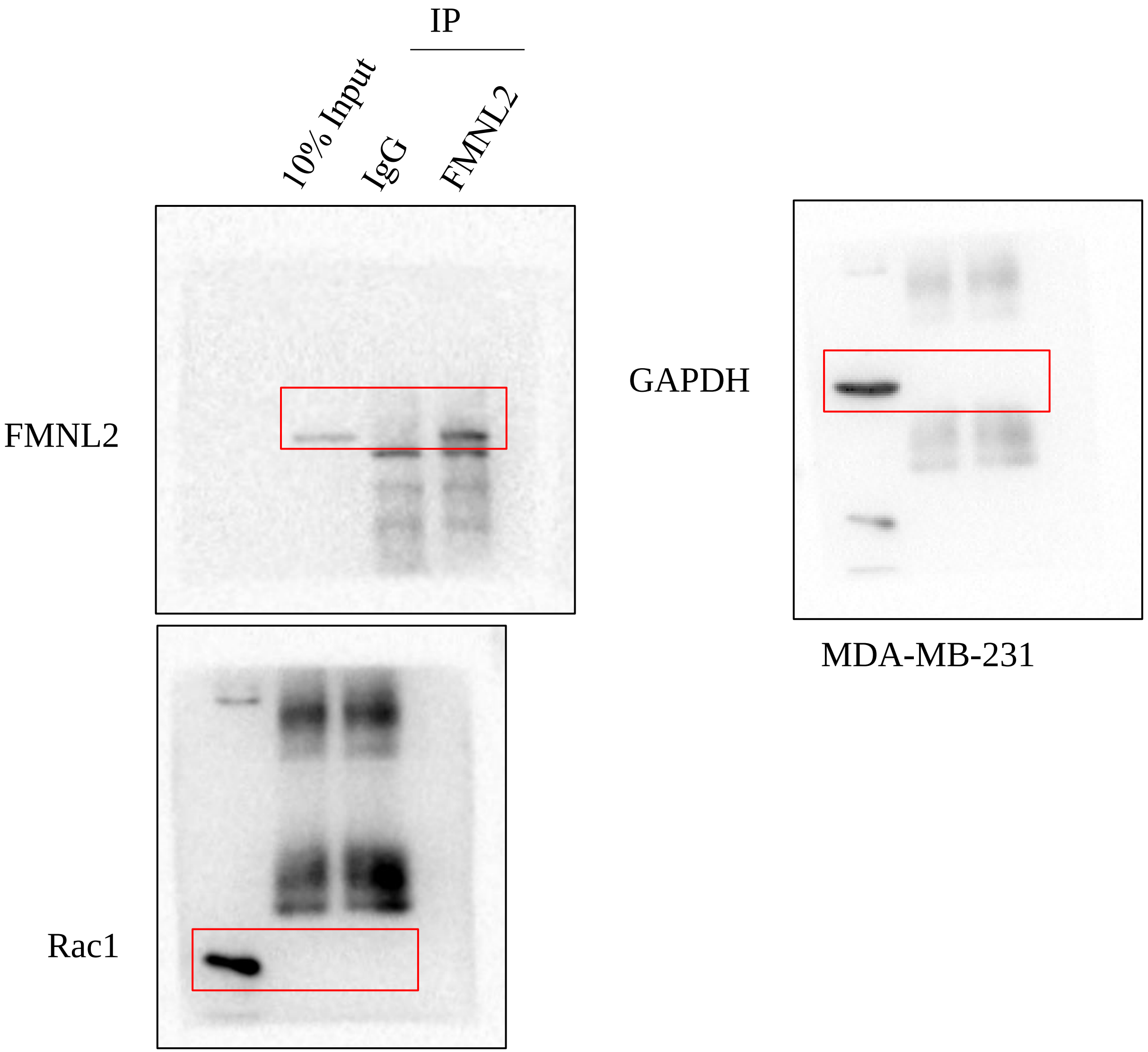
IP
10% Input
IgG
FMNL2
FMNL2
Rac1
GAPDH
MDA-MB-231

Fig. S2D

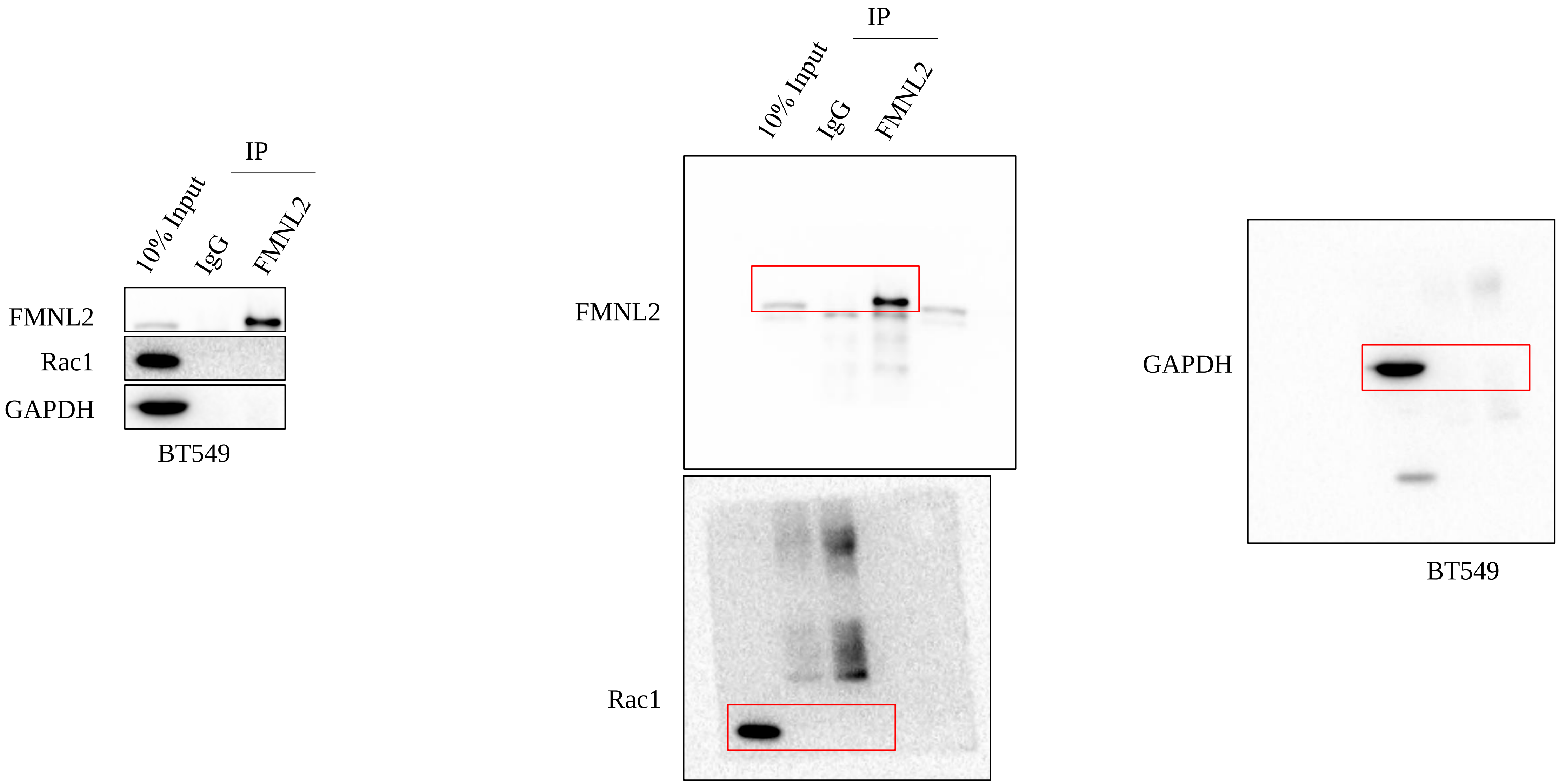
IP
10% Input
IgG
FMNL2
FMNL2
Rac1
GAPDH
BT549
IP
10% Input
IgG
FMNL2
FMNL2
Rac1
GAPDH
BT549

Fig. S3B

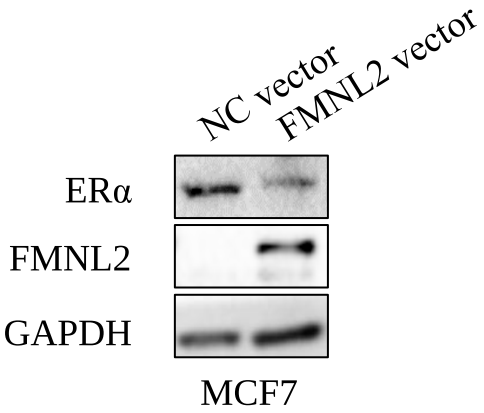
NC vector
FMNL2 vector
ERα
FMNL2
GAPDH
MCF7

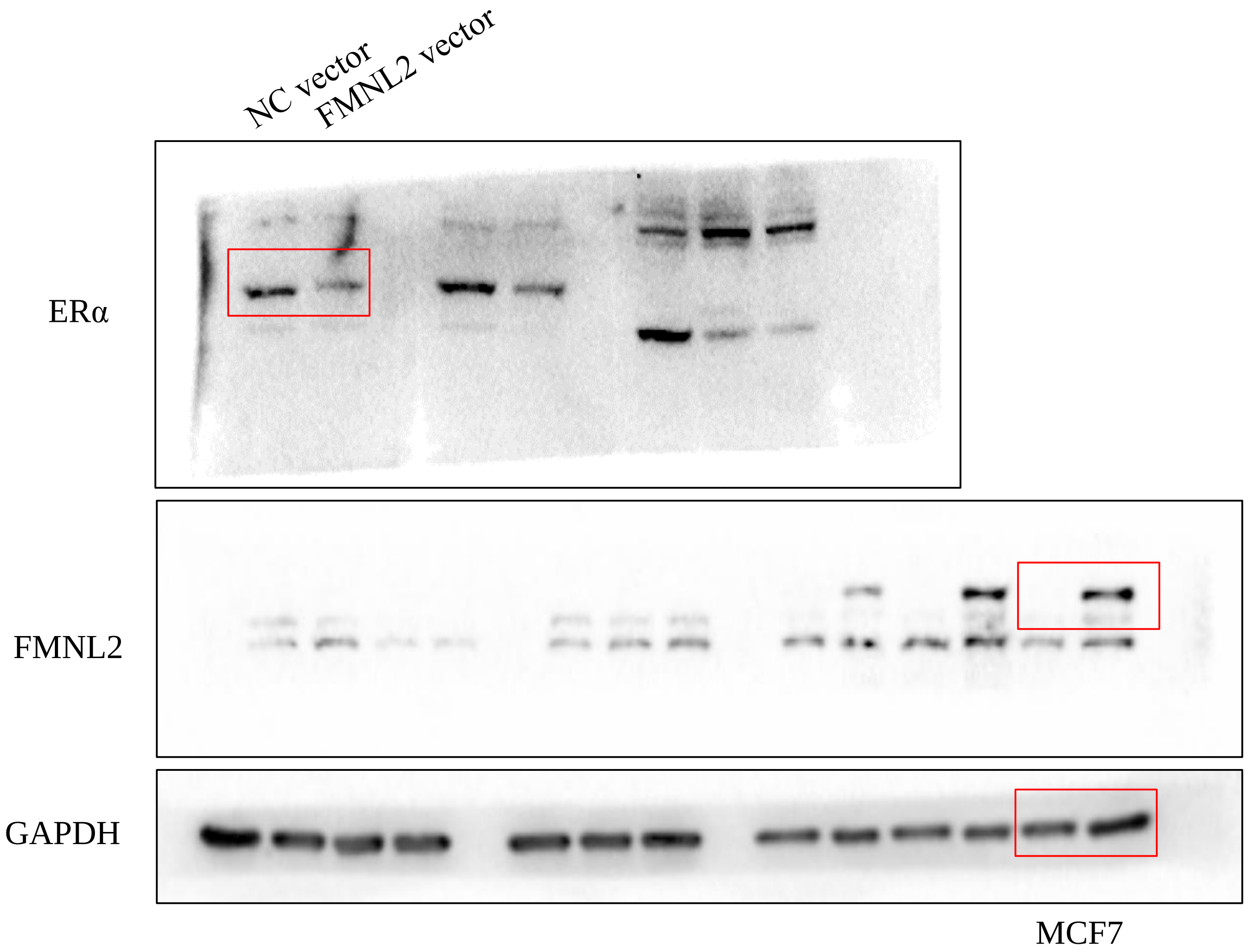
NC vector
FMNL2 vector
ERα
FMNL2
GAPDH
MCF7

Fig. S3C

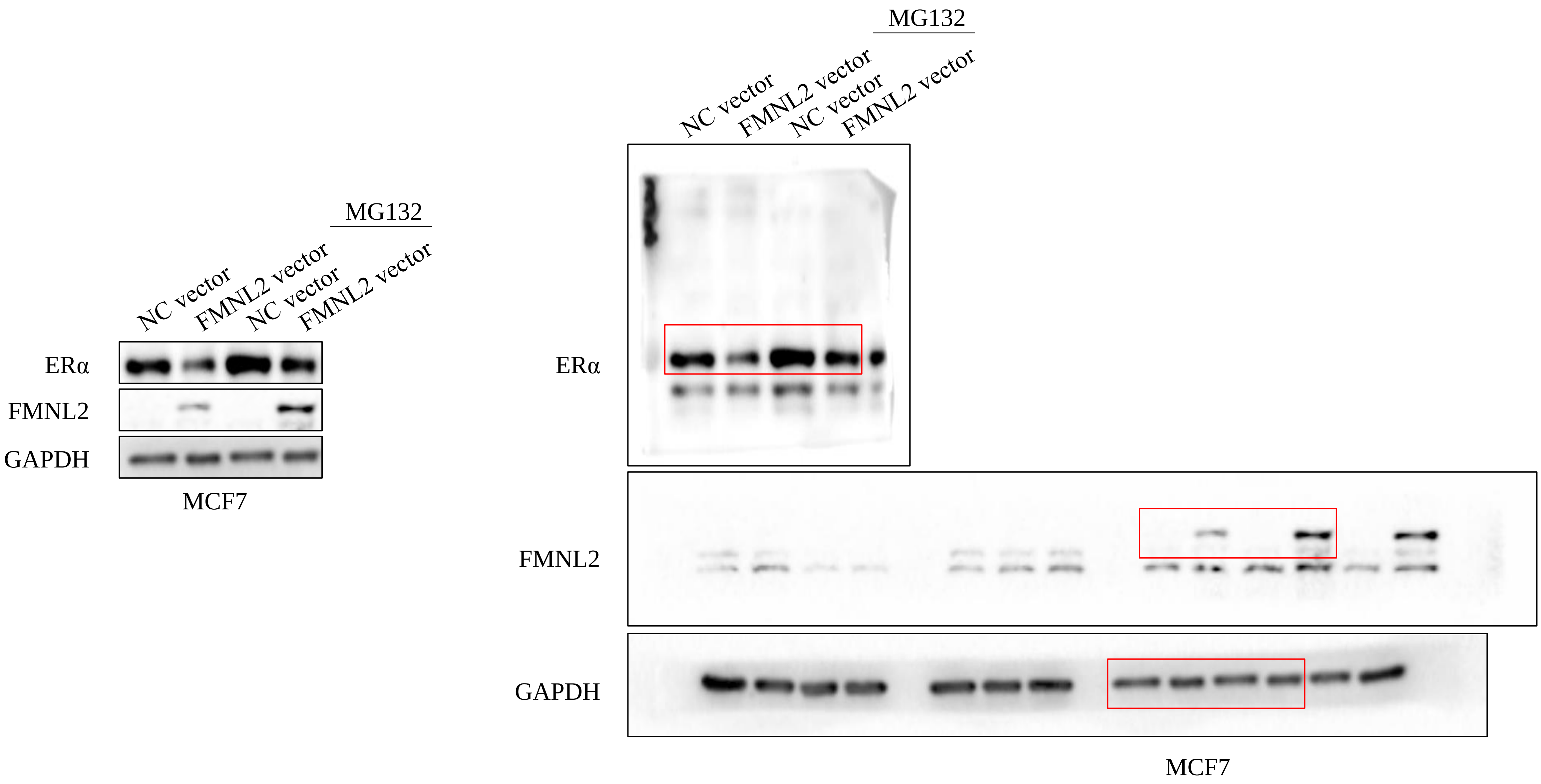
MG132
NC vector
FMNL2 vector
NC vector
FMNL2 vector
ERα
FMNL2
GAPDH
MCF7
MG132
NC vector
FMNL2 vector
NC vector
FMNL2 vector
ERα
FMNL2
GAPDH
MCF7
