# Supplementary material for: FMNL2 suppresses cell migration and invasion of breast cancer: a reduction of cytoplasmic p27 via RhoA/LIMK/Cofilin pathway
Source: Cell Death Discov. 2022 Apr 4;8:155. doi: 10.1038/s41420-022-00964-z (PMC8980084; doi:10.1038/s41420-022-00964-z)
Supplement: Supplementary file 2 — Supplementary information [file 41420_2022_964_MOESM2_ESM.docx]

**Supplementary information**

**Supplementary Figure legends**

**Fig. S1 Effects of FMNL2 silencing on cell motility and migration in breast cancer cells.** **A** After transfection for 48 h, the silencing efficiency of FMNL2 was measured using qRT-PCR in MDA-MB-231 and BT549 cells. **B**, **C** Three independent fields were selected and the images were captured every 15 min for 24 h at 37°C under 20× magnification using live cell imaging assay. Twenty-one cells were chosen from each group for tracking and recording of the movement route of tumor cells. The movement velocity and the accumulated distance were analyzed in MDA-MB-231 cells. **D**, **E** A pipette tip was used to make straight scratch, simulating a wound. The wounded monolayer was incubated with FBS-free medium after washing. Representative images and quantified data of wound healing assay were shown in MDA-MB-231 cells after 24 h and 48 h of simulating a wound. **P*<0.05 *versus* NC siRNA group.

**Fig. S2 Effects of p27 on cell migration and invasion and the interaction between FMNL2 and Rac1 in breast cancer cells.** After transfection for 48 h, cultured cells were processed for indicated assays. **A, B** Representative images and quantified data of transwell assay were shown. Scale bar, 50 μm. **C** The levels of invasion-related molecules were examined by western blotting. **D** Co-IP analysis was used to determine the relationship between FMNL2 and Rac1. **P*<0.05 *versus* NC siRNA group, ^#^*P*<0.05 *versus* sip27 group.

**Fig. S3** **Effects of FMNL2 overexpression on the levels of ERα in MCF7 cells.** After transfection for 48 h, cultured cells were processed for indicated assays. **A**, **B** The mRNA and protein levels of ERα were examined in FMNL2-overexpressing MCF7 cells using qRT-PCR and western blotting. **C** Cultured cells were incubated with MG132 (5 μM) for 12 h as indicated, then the levels of FMNL2 and ERα were examined using western blotting. **P*<0.05 *versus* NC vector group.

**Supplementary Table legends**

**Table. S1** Target sequences of FMNL2 siRNA, p27 siRNA and negative control siRNA.

**Table. S2** Primers used in the qRT-PCR analysis.
