# Supplementary figures and images for: FMNL2 suppresses cell migration and invasion of breast cancer: a reduction of cytoplasmic p27 via RhoA/LIMK/Cofilin pathway
Source: Cell Death Discov. 2022 Apr 4;8:155. doi: 10.1038/s41420-022-00964-z (PMC8980084; doi:10.1038/s41420-022-00964-z)

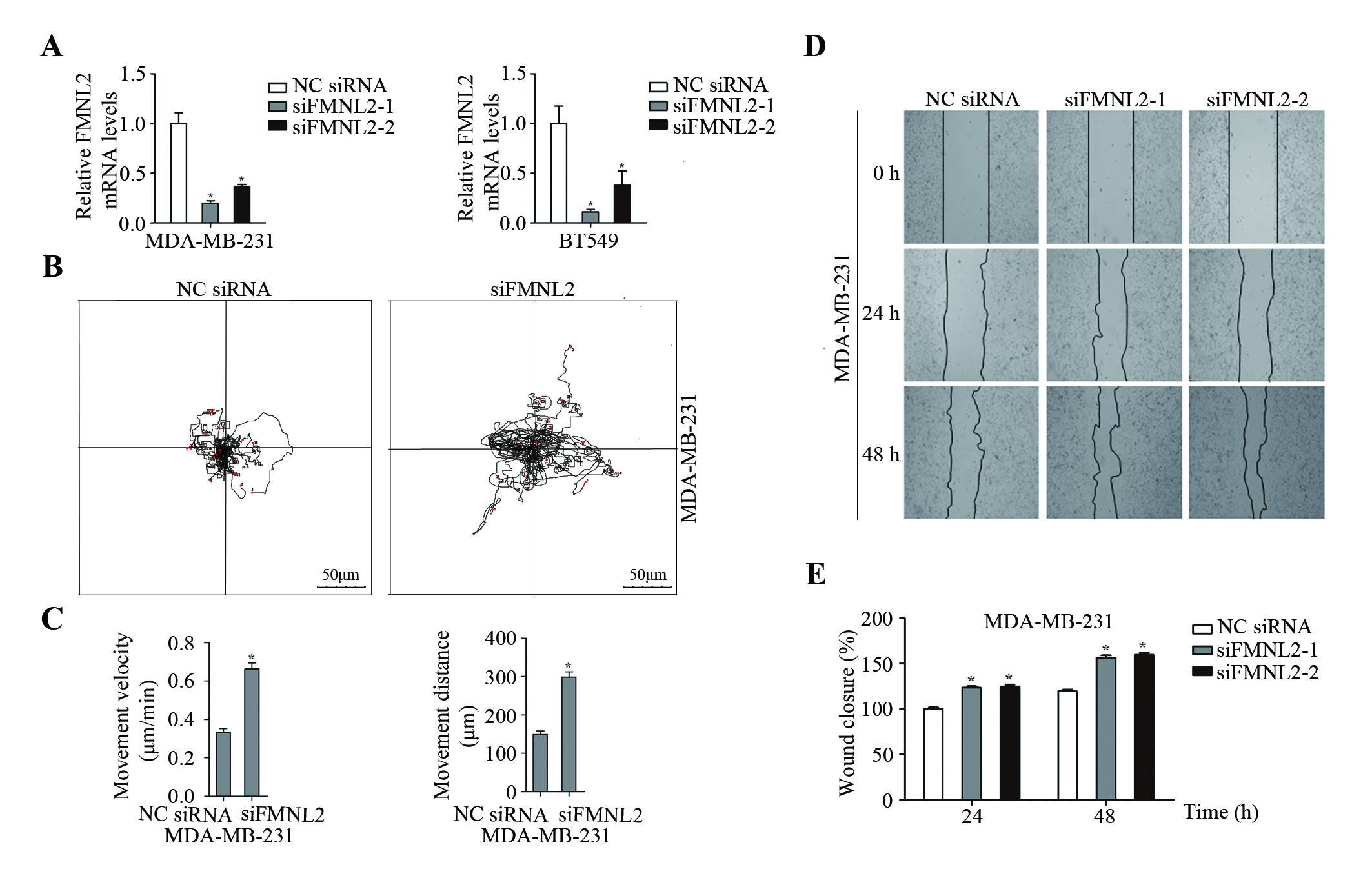

Supplement: Supplementary file 3 — Figure S1 [file 41420_2022_964_MOESM3_ESM.tif]

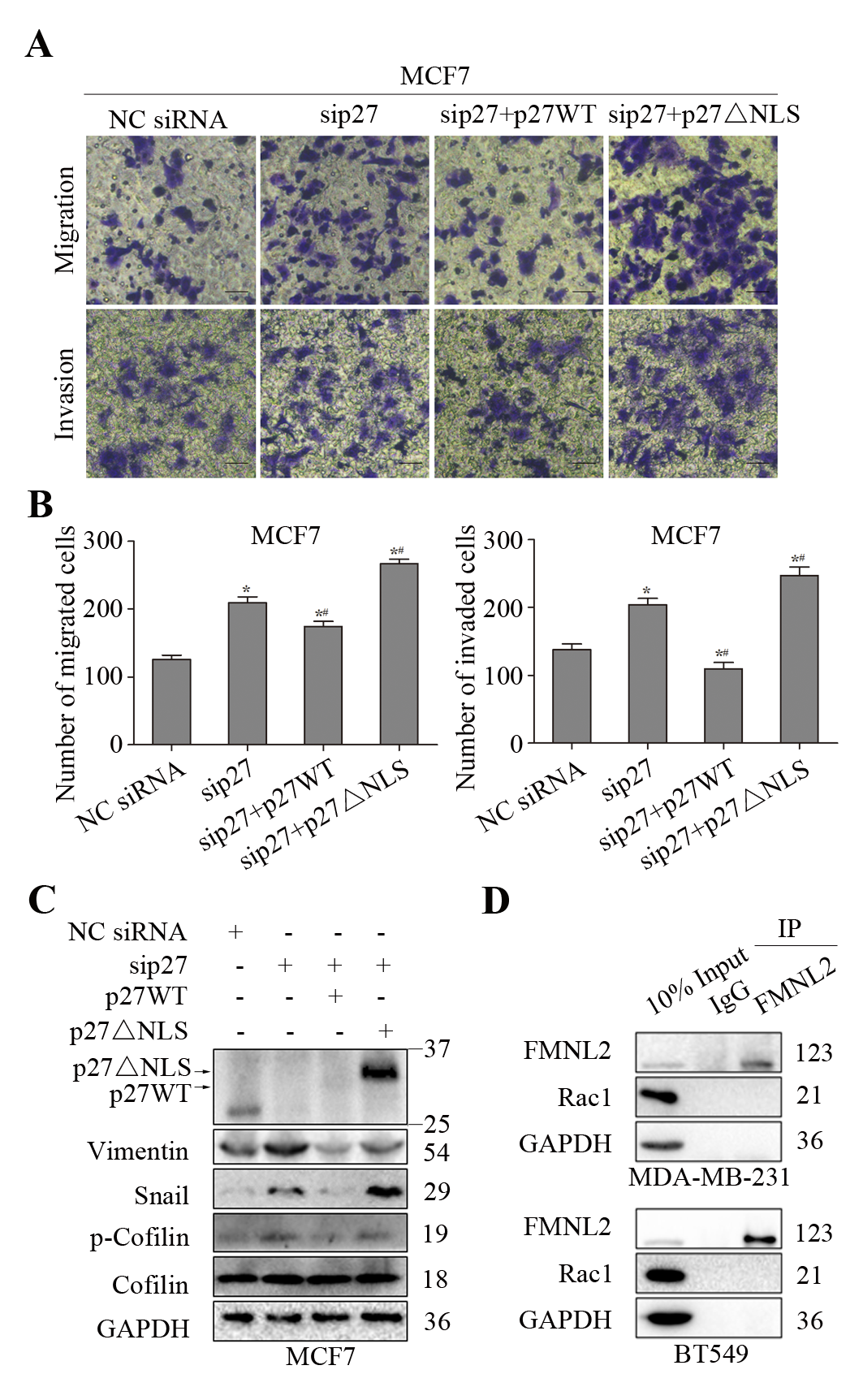

Supplement: Supplementary file 4 — Figure S2 [file 41420_2022_964_MOESM4_ESM.tif]

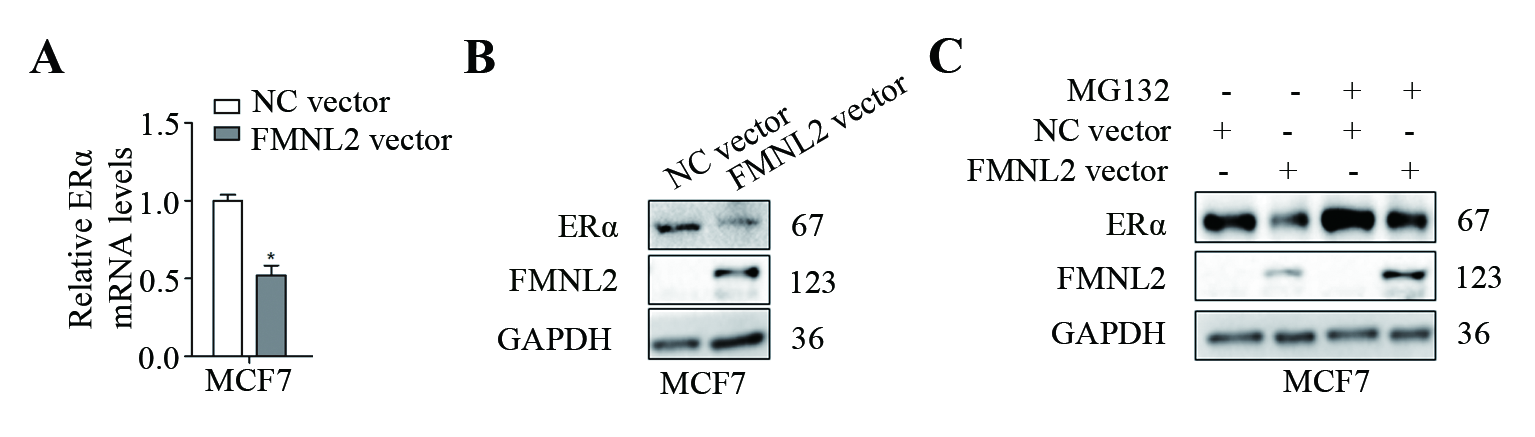

Supplement: Supplementary file 5 — Figure S3 [file 41420_2022_964_MOESM5_ESM.tif]
