# Supplementary material for: FMNL2 suppresses cell migration and invasion of breast cancer: a reduction of cytoplasmic p27 via RhoA/LIMK/Cofilin pathway
Source: Cell Death Discov. 2022 Apr 4;8:155. doi: 10.1038/s41420-022-00964-z (PMC8980084; doi:10.1038/s41420-022-00964-z)
Supplement: Supplementary file 6 — Table S1 [file 41420_2022_964_MOESM6_ESM.docx]

**Table. S1** Target sequences of FMNL2 siRNA, p27 siRNA and negative control siRNA.

| siRNA | sequences (5’-3’) |
| --- | --- |
| siFMNL2-1 | sense: 5’-GCGUGUUCAAGAAUCUACATT-3’ |
|  | antisense: 5’-UGUAGAUUCUUGAACACGCTT-3’ |
| siFMNL2-2 | sense: 5’-GCCCUUGUCUUAGAACUGUTT-3’ |
|  | antisense: 5’-ACAGUUCUAAGACAAGGGCTT-3’ |
| sip27 | sense: 5’-GCAACCGACGAUUCUUCUATT-3’ |
|  | antisense: 5’-UAGAAGAAUCGUCGGUUGCTT-3’ |
| NC siRNA | sense: 5’-UUCUCCGAACGUGUCACGUTT-3’ |
|  | antisense: 5’-ACGUGACACGUUCGGAGAATT-3’ |
