# Supplementary material for: FMNL2 suppresses cell migration and invasion of breast cancer: a reduction of cytoplasmic p27 via RhoA/LIMK/Cofilin pathway
Source: Cell Death Discov. 2022 Apr 4;8:155. doi: 10.1038/s41420-022-00964-z (PMC8980084; doi:10.1038/s41420-022-00964-z)
Supplement: Supplementary file 7 — Table S2 [file 41420_2022_964_MOESM7_ESM.docx]

**Table. S2** Primers used in the qRT-PCR analysis.

| Gene | Primer sequences |
| --- | --- |
| FMNL2 | Forward: 5’-TAATCAGCATTAGCATTTCTGAGG-3’ |
|  | Reverse: 5’-AGGAGAGTAAGGCCAGGTTCC-3’ |
| Vimentin | Forward: 5’-TGACATTGAGATTGCCACCTACAG-3’ |
|  | Reverse: 5’-TCAACCGTCTTAATCAGAAGTGTCC-3’ |
| Snail | Forward: 5’- TCTGGTTCTGTGTCCTCTGC-3’ |
|  | Reverse: 5’- TTCCCAGTGAGTCTGTCAGC-3’ |
| RhoA | Forward: 5’-CAGAAAAGTGGACCCCAGAA-3’ |
|  | Reverse: 5’-GCAGCTGCTCTCGTAGCCATTTC-3’ |
| ROCK1 | Forward: 5’-AACATGCTGCTGGATAAATCTGG-3’ |
|  | Reverse: 5’-TGTATCACATCGTACCATGCCT-3’ |
| Cdc42 | Forward: 5’-ATTATGACAGACTACGACCGCT-3’ |
|  | Reverse: 5’-AGTGGTGAGTTATCTCAGGCA-3’ |
| Sep7 | Forward: 5’-AGGGCAGCTGACTAAGAGCCC-3’ |
|  | Reverse: 5’-TCATTT GCTCATGGCGCCGCT-3’ |
| GAPDH | Forward: 5’-AGAAGGCTGGGGCTCATTTG-3’ |
|  | Reverse: 5’-AGGGGCCATCCACAGTCTTC-3’ |
